# Age-Specific Gene Expression Profiles of Rhesus Monkey Ovaries Detected by Microarray Analysis

**DOI:** 10.1155/2015/625192

**Published:** 2015-09-02

**Authors:** Hengxi Wei, Xiangjie Liu, Jihong Yuan, Li Li, Dongdong Zhang, Xinzheng Guo, Lin Liu, Shouquan Zhang

**Affiliations:** ^1^Guangdong Provincial Key Lab of Agro-Animal Genomics and Molecular Breeding, National Engineering Research Center for Breeding Swine Industry, College of Animal Science, South China Agricultural University, Guangzhou, Guangdong 510642, China; ^2^State Key Laboratory of Medicinal Chemical Biology, Department of Cell Biology and Genetics, College of Life Sciences, Nankai University, Tianjin 300071, China

## Abstract

The biological function of human ovaries declines with age. To identify the potential molecular changes in ovarian aging, we performed genome-wide gene expression analysis by microarray of ovaries from young, middle-aged, and old rhesus monkeys. Microarray data was validated by quantitative real-time PCR. Results showed that a total of 503 (60 upregulated, 443 downregulated) and 84 (downregulated) genes were differentially expressed in old ovaries compared to young and middle-aged groups, respectively. No difference in gene expression was found between middle-aged and young groups. Differentially expressed genes were mainly enriched in cell and organelle, cellular and physiological process, binding, and catalytic activity. These genes were primarily associated with KEGG pathways of cell cycle, DNA replication and repair, oocyte meiosis and maturation, MAPK, TGF-beta, and p53 signaling pathway. Genes upregulated were involved in aging, defense response, oxidation reduction, and negative regulation of cellular process; genes downregulated have functions in reproduction, cell cycle, DNA and RNA process, macromolecular complex assembly, and positive regulation of macromolecule metabolic process. These findings show that monkey ovary undergoes substantial change in global transcription with age. Gene expression profiles are useful in understanding the mechanisms underlying ovarian aging and age-associated infertility in primates.

## 1. Introduction

In the past few decades, ovarian aging has been considered one of the most detrimental factors contributing to pregnancy failure, and the age-related decline in female fecundity has distinct implications in view of the current trend of postponing childbearing [1, 2]. Premature ovarian failure (POF), also known as premature menopause, affects 1%-2% of women younger than 40 years of age and 0.1% of women younger than 30 years of age [3] and is another common cause of female infertility [4].

Rhesus monkeys (*Macaca mulatta*) are the most frequently used nonhuman primate model, because they live in close association with each other and have many similarities with humans in anatomy, physiology, and genetics [5]. Many parameters including circadian rhythm, seasonality, and hormonal effects on the brain in the rhesus macaque have been studied using species-specific gene microarrays [6]. Experimental analysis of the aging process (or senescence) has been challenging [7]. As the value of the aging model of the rhesus monkey increases with completion of the sequencing of the rhesus genome [8], it is necessary to make more efficient and extensive use of this model to understand the processes underlying aging and disease in humans [9]. Several theories of aging have been proposed, including free radical [10], glycation [11], and caloric restriction [12, 13] theories. Mechanisms of aging that involve mitochondria [14], DNA damage and repair [15], DNA methylation [16], telomeres [17], and cellular senescence and apoptosis [18] have also been proposed. The primary reason for the decline with age in female fertility is the gradual loss of oocytes and follicles, which were formed during embryonic development [19]. Chromosomal abnormalities, mitochondrial DNA mutations, telomere shortening, and even aging itself have been suggested to be related to the decreased oocyte quality associated with aging, and it was concluded that ovarian aging may be only a specific reflection of general aging [20]. The new hypothesis of protein glycation and that of oxidative stress and mitochondrial dysfunction, which are closely related to calcium regulation, are considered to be the main cellular and molecular mechanisms underlying aging of the ovarian follicle [21].

Previous studies identified genes that are differentially expressed in an age-dependent manner using microarray analysis of mouse oocytes, ovary, and ovarian surface epithelial cells as well as human oocytes [22–27]. In rhesus monkey, microarray analysis has been performed to identify the mechanisms involved in the brain's white matter aging and corpus luteum regression [28, 29]. However, there is still a paucity of knowledge about primate ovarian failure, because the ovary is a functional unit, and most age-related changes in gene expression are species-specific [30, 31]. In order to investigate the molecular and biological mechanisms of ovarian aging and to identify genes that may play a role in oocyte/ovarian aging or POF, we performed genome-wide microarray analysis of ovaries from young, middle-aged, and old rhesus monkeys. The genes affected by aging may serve as important targets for delaying ovarian aging or for the clinical treatment of POF.

## 2. Materials and Methods

### 2.1. Animals

All animal procedures were performed according to guidelines developed by the China Council on Animal Care, and protocols were approved by the Animal Care and Use Committee of Guangdong Province, China. The approval ID is SCXK (Guangdong) 2004-0011 and the permit number is SYXK (Guangdong) 2007-0081. The monkeys were housed in ordinary animal facilities in a temperature-controlled (25°C) and light-regulated (12 h light/12 h dark) room and fed commercial nonhuman primate diets twice daily, supplemented with fresh fruits and water ad libitum. Female rhesus monkeys were all reproductive and healthy and randomly chosen from three different aged groups according to experimental design and supplied by Guangxi Grandforest Scientific Primate Company, Ltd.

The experimental design is briefly as follows: the old group (18 to 19 years) had lost their reproductive capacity; the middle-aged group (7 to 8 years) had good reproductive capacity; and the young group (3 to 4 years) had normal estrous cycles but had never bred. The ovary collection time of young and middle-aged monkeys was approximately at the proestrus stage according to their reproductive records. A minimal number of 3 monkeys of each group were used in this study to minimize ethical concerns. All individual ovaries from the three groups were examined histologically and by microarray analysis to investigate the ovary status, the gene expression profiles, and the differentially expressed genes between groups.

### 2.2. Ovary Collection

All monkeys were housed under the same conditions, and the ovary collection was conducted in October. Monkeys were deeply anaesthetized with 4 mL of 3% sodium pentobarbital per kilogram of body weight by intravenous injection and sacrificed by femoral artery exsanguination and, simultaneously, transcardially perfused with 4 L of Krebs-Henseleit buffer (prepared under RNase-free conditions and containing 6.41 mM Na_2_HPO_4_, 1.67 mM NaH_2_CO_3_, 137 mM NaCl, 2.68 mM KCl, 5.55 mM glucose, 0.34 mM CaCl_2_, and 2.14 mM MgCl_2_, pH 7.4) at 4°C [29]. Surgery was carried out according to standard operating procedures. Some organs were collected by other institutions, and only one ovary of each monkey was allocated to our laboratory. The ovaries were cut in half vertically at their widest point, and one half was immediately frozen in liquid nitrogen and sent to CapitalBio Corporation (Beijing, China) in dry ice for microarray analysis; the other half was transported to our laboratory in dry ice for histological and qRT-PCR analysis.

### 2.3. Histological Analysis and Follicle Counting

The half-ovaries from young, middle-aged, and old monkeys were again cut in half vertically at their widest points. One part of the tissue was randomly selected and stored in liquid nitrogen for further qRT-PCR validation of microarray results. The other part was immersed in 4% paraformaldehyde for 24 h and then dehydrated in increasing concentrations of ethanol and in xylene. The tissues were embedded in paraffin, and sections of 5 *μ*m were cut and aligned on glass microscope slides. After deparaffinization in xylene, the sections were rehydrated through decreasing concentrations of ethanol in water and stained with hematoxylin and eosin Y. The tissue sections were dehydrated again, coverslips were applied with neutral gum, and sections were viewed and photographed with an Olympus BX53F microscope. To avoid counting a structure twice, one out of every 20 serial sections was analyzed for the number of follicles in different developmental stages using slightly modified standard methods [32, 33], and all the whole sections were analyzed. Primordial and primary follicles were identified by the presence of an oocyte surrounded by a single layer of flat or cuboidal cells. Secondary follicles were characterized as having more than one layer of granulosa cells with no visible antrum. Antral follicles possessed areas of follicular fluid (antrum) or a single large antral space. These follicles with normal morphology were scored as healthy follicles. And atretic follicles were characterized with shanked nuclei and degenerate oocytes and loosened layer of granulosa cells. And the relic atretic follicles were defined as atretic follicles without clear oocyte nuclei, which were not counted as atretic follicles in our statistics. A one-way ANOVA analysis was used to assess the statistical significance of follicles among different groups.

### 2.4. RNA Isolation and Microarray Analysis

RNA was isolated from each half-ovary sent to CapitalBio for microarray detection and validation of the microarray data by quantitative real-time polymerase chain reaction (qRT-PCR). Total RNA was extracted using TRIzol reagent (Invitrogen, Carlsbad, CA, USA) and further purified using the Qiagen RNeasy Mini Kit (Germantown, MD, USA) according to the manufacturer's instructions. RNA quality was assessed by formaldehyde agarose gel electrophoresis, and the RNA was quantitated spectrophotometrically.

The* M. mulatta* Genome Array (Affymetrix) containing 47,000 transcripts was obtained from CapitalBio Corporation (Beijing, China). RNA derived from each of the nine monkeys was run on an individual microarray, and microarray experiments were performed as described previously [34]. After hybridization, the arrays were scanned with LuxScan 10 K-A scanner (CapitalBio) and the data from the obtained images were extracted using LuxScan 3.0 software (CapitalBio). A space and intensity-dependent normalization based on a LOWESS program was employed [35]. For each test and control sample, two hybridization processes were performed by using a reversal of the fluorescent dye strategy. Only genes with consistent differential expression (both above 1.5-fold change) in both microarray assays were selected as differentially expressed genes. The description of this microarray study follows the Minimum Information About a Microarray Experiment (MIAME) guidelines [36], and the data was submitted to Gene Expression Omnibus (GEO) with accession number of GSE44533.

### 2.5. Validation of Microarray Results by qRT-PCR

Twenty-five differentially expressed genes were randomly selected and validated with the same RNA preparations that were used to generate microarray data, and 8 out of the 25 genes were again validated with the new RNA samples from the same ovary tissues by qRT-PCR. Beta-actin was used as an internal standard. The gene-specific qRT-PCR primers were designed according to the coding sequences (Table 1). Briefly, total RNA from each of the nine monkeys was digested with DNase I (TaKaRa, Dalian, China). First-strand cDNAs were synthesized with oligo(dT) primers using a PrimeScript II 1st Strand cDNA Synthesis Kit (code D6210A, TaKaRa). Quantitative RT-PCR was performed using the SsoFast EvaGreen Supermix (Bio-Rad, Hercules, CA) and CFX96 Quantitative Real-Time PCR Detection System (Bio-Rad). Each 20 *μ*L qRT-PCR mixture included 10 *μ*L SsoFast EvaGreen Supermix, 1 *μ*L cDNA, 0.2 *μ*M primers, and 8.6 *μ*L double-distilled water. PCR was carried out under conditions of initial denaturation at 95°C for 30 sec, followed by 40 cycles of denaturation at 95°C for 5 sec, annealing at 60°C for 30 sec, and extension at 72°C for 30 sec. A melting curve was plotted from 65°C to 95°C to check the specificity of the amplified product. Each of the amplifications was carried out in duplicate, and the mean values were calculated using the ΔΔ*C*_*t*_ method. The results (fold change) were determined and expressed as 2^ΔΔ*C*_*t*_^ according to the following formula:

(1) $$\begin{array}{c} \Delta \Delta C_{t}=\left(C_{tij}-C_{t\beta \text{-actin}j}\right)-\left(C_{ti1}-C_{t\beta \text{-actin1}}\right), \end{array}$$

where *C*_*tij*_ and *C*_*tβ*-actin*j*_ are the *C*_*t*_ values for gene *i* and *β*-actin, respectively, in sample *j*. *C*_*ti*1_ and *C*_*tβ*-actin1_ are the *C*_*t*_ values in sample 1, expressed as the standard [37]. Student's *t*-test or one-way ANOVA analysis was used to assess the statistical significance of differential expression levels of each gene among the three groups of monkeys using GraphPad Prism 5.0 software.

### 2.6. GO Terms and KEGG Pathway Analysis

Gene Ontology (GO) terms and Kyoto Encyclopedia of Genes and Genomes (KEGG) pathway were analyzed using the free, web-based Molecular Annotation System 3.0 (MAS 3.0, http://bioinfo.capitalbio.com/mas3/). Gene products were analyzed according to the GO ontologies molecular function, biological process, and cellular component. The *P* values less than 0.01 were considered significance. All differentially expressed genes were input, and the results obtained with the categories of GO terms and the KEGG pathways were presented in the form of a Microsoft Excel 2007 spreadsheet.

Due to the lack of comprehensive gene annotation information for* M. mulatta*, we assumed that the orthologous genes conserved between human and* M. mulatta* were functionally conserved. The relationships between human and* M. mulatta* genes were based on Ensembl release 74 (http://www.ensembl.org/) and retrieved using Bio-Mart (http://www.biomart.org/). We used human-*M. mulatta* orthologs to identify the differently expressed genes. The functional annotation of these human genes in the functional category and KEGG pathway was performed using DAVID Bioinformatics Resources [38]. Probabilities were evaluated by Bonferroni correction, and values less than 0.001 were considered significant.

## 3. Results and Discussion

### 3.1. Aged Monkey Ovaries Show Morphological Changes and Differentially Expressed Genes

**Figure 1:**
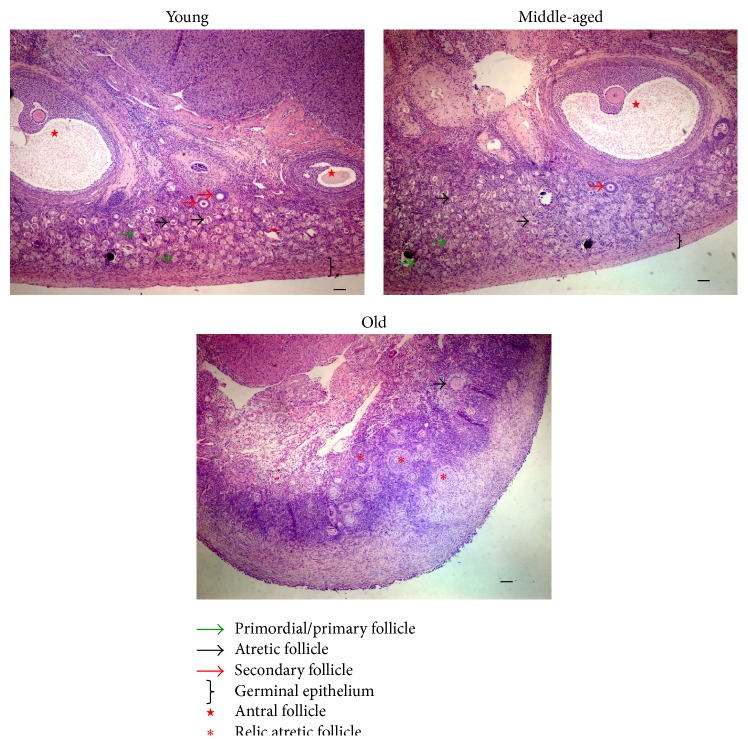
Morphology of monkey ovaries of different ages. Hematoxylin and eosin Y-stained sections of ovaries from monkeys of indicated ages. Key to structures indicated by symbols appears below images. Bar = 100 *μ*m.

Our results showed great change in ovarian morphology of different aged monkeys. In young and middle-aged monkey ovaries, follicles at various developmental stages, including many primordial and primary, several secondary, and mature follicles, were observed. The numbers of primordial and primary and secondary follicles significantly decreased with age, and the number of antral follicles increased significantly from young to middle-aged ovary, as we previously reported in [39], whereas only a few primary or atretic follicles and no antral follicles were seen in the ovaries of old monkeys (Figure 1). Herein, we found that the total follicles and the number of healthy follicles significantly decreased with aging, and the number of atretic follicles increased significantly in middle-aged groups and then decreased in the old groups when compared to young animals (Table 2). The morphology of the ovarian surface epithelium also changed with age. In young ovaries, the germinal epithelium was smooth, thick, and clearly distinguishable from the cortex. In the middle-aged ovary, the germinal epithelium was thin, and, in the old ovary, epithelial fibrosis had taken place, making the epithelium indistinguishable from the cortex (Figure 1).

**Figure 2:**
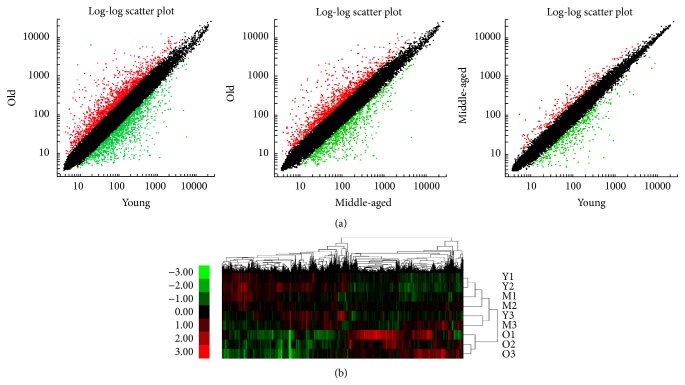
Gene expression profiles of monkey ovaries of different ages. (a) Log-log scatter plot analysis between groups of different ages. It shows good quality of microarray data; the red and green dots indicate upregulated and downregulated genes, respectively. (b) Hierarchical cluster of differentially expressed genes in old (O), middle-aged (M), and young (Y) groups. The expression level of each gene is standardized to a mean value of 0 and standard deviation (SD) of 1. The mean value is represented by black, gene expression above the mean level is represented by red, and expression below the mean is represented by green. The intensity of the pseudocolor reflects the number of SDs from the mean, as indicated in key at left. 1–3: animals 1–3 in each group.

**Figure 3:**
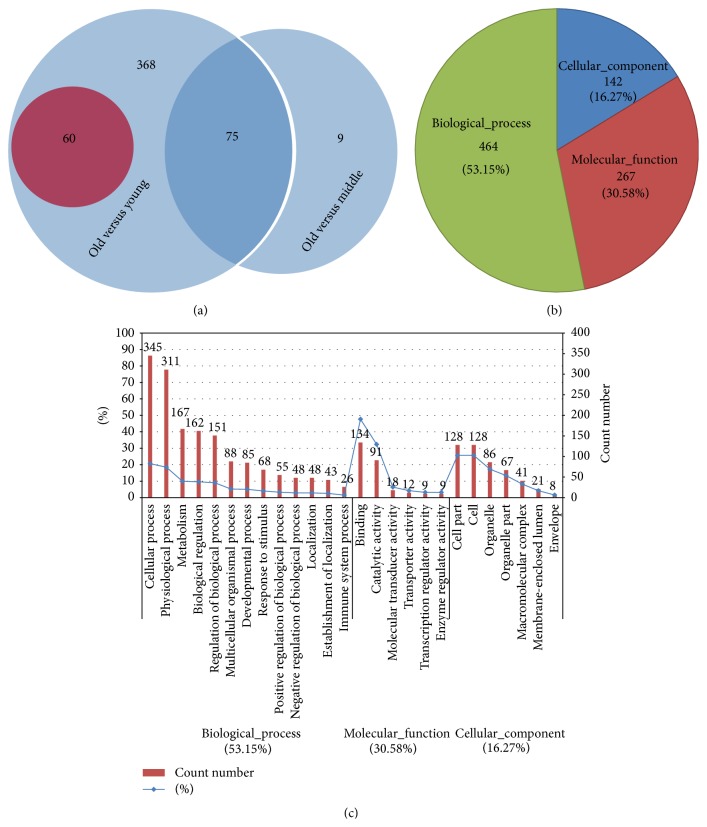
The age-related ovaries' differentially expressed genes and their GO term analysis. (a) Venn diagram of differentially expressed genes in different aged monkey ovaries. The circle with red and blue background indicates upregulated and downregulated genes, respectively. The number of differentially expressed genes was marked on the corresponding area. (b) Pie diagram of GO mapping of the total 512 differentially expressed genes found in the present study. The gene count numbers and their ratio were marked in the diagram. (c) The GO term analysis of the total 512 differentially expressed genes. The number over each column is the gene count number; the blue line shows the percent of each column item in the corresponding categories of biological process, molecular function, and cellular component.

Nine microarrays representing ovaries collected from three monkeys in each of the age groups were analyzed. Approximately 47,000 probe sets detected 35,000 genes (see GSE44533, in GEO database). The log-log scatter plot analysis showed good quality of the microarray assays (Figure 2(a)). The remarkable influence of age was demonstrated by hierarchical cluster analysis (Figure 2(b)). After SAM analysis of the microarray data, 503 genes were differentially expressed (fold change, +1.5 or −1.5) between the old and the young groups; of these, 60 were upregulated in the ovaries of old monkeys (Figure 3(a)). Only 84 genes were differentially expressed between the old and middle-aged groups, all of which were downregulated in the ovaries of old monkeys. These two sets of 503 and 84 differentially expressed genes shared 75 common genes (Figure 3(a)). No difference in gene expression was found between the middle-aged and young groups. Of the total of 512 differentially expressed genes, the functions of 264 genes, of which 35 were upregulated and 229 were downregulated, are still unknown (highlighted in Table S1 in Supplementary Material available online at http://dx.doi.org/10.1155/2015/625192).

The current work firstly presents the global gene expression profile of ovarian aging in rhesus monkey. Although previous research has focused on ovarian aging in mice [26, 40, 41], the aging process is quite different between mice and humans [30], emphasizing the need for human or nonhuman primate tissue to elucidate the mechanism of aging of human ovaries. The gene expression profiles of metaphase II oocytes derived from women of different ages suggest that cell cycle, oxidative stress and DNA repair, meiosis and spindle function, and ubiquitination might be affected by age [22]; however, the processes underlying ovarian aging remain obscure. Although our study is limited, as whole ovaries were sampled which contain multiple cell types [27, 42], hundreds of differentially expressed genes were found related to age, and such global gene expression profiles of monkey ovaries of different ages constitute a useful resource. In the future, investigations of age-related differential gene expression in individual cell types are warranted. Although not focused on aging, a novel resource of nonhuman primate oocytes and preimplantation embryos has already been established which might facilitate gene expression pattern analysis in single cell types [31, 43].

### 3.2. Validation of Microarray Data by qRT-PCR

**Figure 4:**
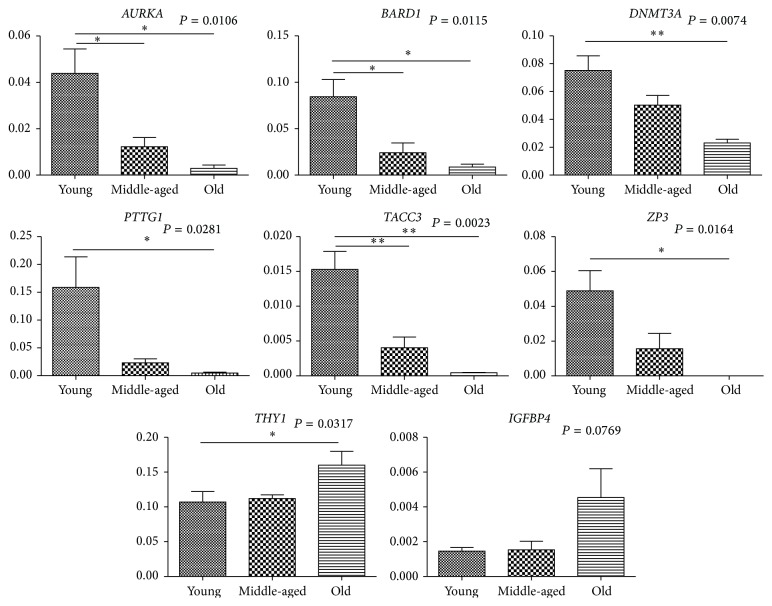
Validation of microarray results by qRT-PCR analysis of new RNA samples. ^*∗*^ *P* < 0.05; ^*∗∗*^ *P* < 0.01; *P* values are indicated for each gene.

Quantitative RT-PCR was performed to validate 25 differentially expressed genes selected randomly (5 upregulated and 20 downregulated in old ovaries) using the same RNA preparations used to generate microarray data (Table 3). As expected, MRAP and MMP9 were expressed at significantly different levels (*P* < 0.05) in the old and young groups and were upregulated in the old group. THY1, Loc717872, and IGFBP4 showed the same trend toward upregulation in old ovaries, although no significant differences were found using Student's *t*-test. Microarray and qRT-PCR data were in good agreement with most genes that were significantly downregulated, or trending in that direction, in old ovaries. However, some significant differential expression identified by microarray analysis (e.g., of* BARD1*,* LOC707199*,* IGF2BP3*, and* XRCC6*) was not validated by qRT-PCR analysis (Table 3). Surprisingly, microarray analysis found that* THBD* was downregulated in old ovaries; however, qRT-PCR analysis showed a trend toward upregulation (Table 3). Moreover, some genes were identified as significantly differentially expressed by qRT-PCR but not by microarray analysis, including* MRAP*,* HELLS*,* CDK1*, and* UBE2C* in old versus middle-aged ovaries (Table 3). This could result from an experimental artifact or the different significance levels calculated [37]. Furthermore, eight out of the 25 genes were randomly selected and validated by qRT-PCR analysis with new RNA samples, and the results were in agreement with those of the previous validation and microarray analysis (Figure 4), indicating the reliability of the microarray data.

### 3.3. GO Terms and KEGG Pathway Analysis

All differentially expressed genes were input into MAS 3.0 and assigned individual GO terms for* M. mulatta*, with 53.15%, 30.58%, and 16.27% representing the main functional categories of biological process, molecular function, and cellular component, respectively (Figure 3(b)). For the ontology “biological process,” the main functional categories were “cellular process,” “physiological process,” “metabolism,” and “biological regulation.” For the ontology “molecular function,” the differentially expressed genes were mainly enriched in “binding” and “catalytic activity.” For the ontology “cellular component,” the “cell part,” “cell,” and “organelle” were the first three categories (Figure 3(c) and Table S2). The GO term analysis showed that great changes of nucleus and microtubules of ovary cells might take place mainly by abnormal transcription regulation, DNA repair, and ligand binding affected by age. Cell cycle, oxidative stress and DNA repair, meiosis and spindle function, and ubiquitination have been considered to be affected by age [22], which is consistent with our results. Our findings for rhesus monkey ovary showed that many differentially expressed genes were involved in transcription regulation, cell cycle, DNA replication and repair, and some other important, aging-related biological processes (Table S2). We found that most of the genes (such as* THY1*,* HELL*, and* ZP3*) related to aging are similar to those previously identified in aging mouse ovaries [26], but some of the differentially expressed genes in pathways are quite different. For example, the important genes* Nfkb1*,* Trp53*, and* Tert* were differentially expressed in mouse, as reported by other groups [17, 25, 26, 44], but no age-related differences in these genes were found in monkeys. However, the genes* NLRP4*,* NLRP11*,* BCL2 L10*,* CYP11A*,* FADS1*, and* XRCC6*, which are related to regulation of NFkB, apoptosis, and TERT function [45–48], were differentially expressed in monkey ovaries. This discrepancy might be due to species-specific differences and/or different ovarian physiological status.

The results also indicated that 11 KEGG pathways were associated with the genes differentially expressed between the old and the young and middle-aged groups (Table S3). A few pathways that have been widely reported to be associated with the aging process, such as “cell cycle” and “TGF-*β*,” appeared in our results. It was reported that the TGF-*β* signaling pathway is constitutively active in aging of myogenic progenitors [49] and brain [50, 51]. We found that these KEGG pathways contained only a limited number of genes due to the lack of comprehensive annotation of the* M. mulatta* genome (Table S3).

### 3.4. KEGG Pathway Analysis with Human-*M. mulatta* Orthologs

**Figure 5:**
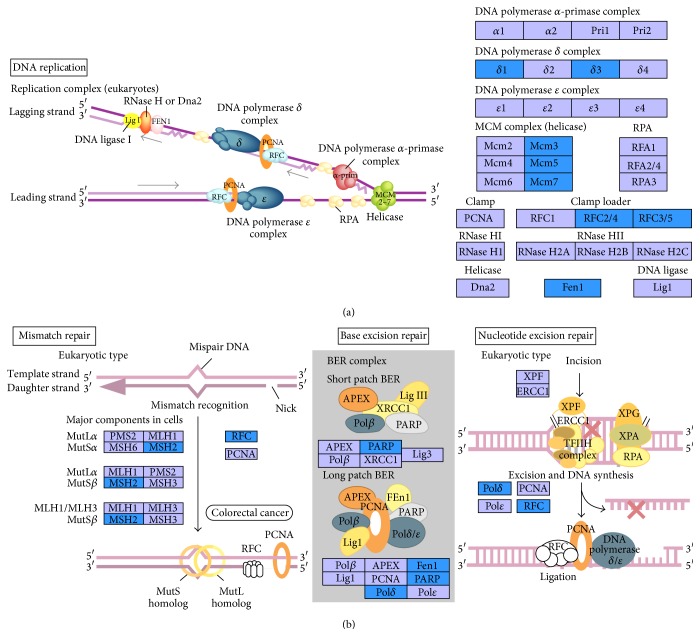
Differentially expressed genes involved in DNA replication pathway (a) and DNA repair pathway (b). Blue boxes backgrounds indicate genes differentially downregulated in old* M. mulatta* ovaries.

**Figure 6:**
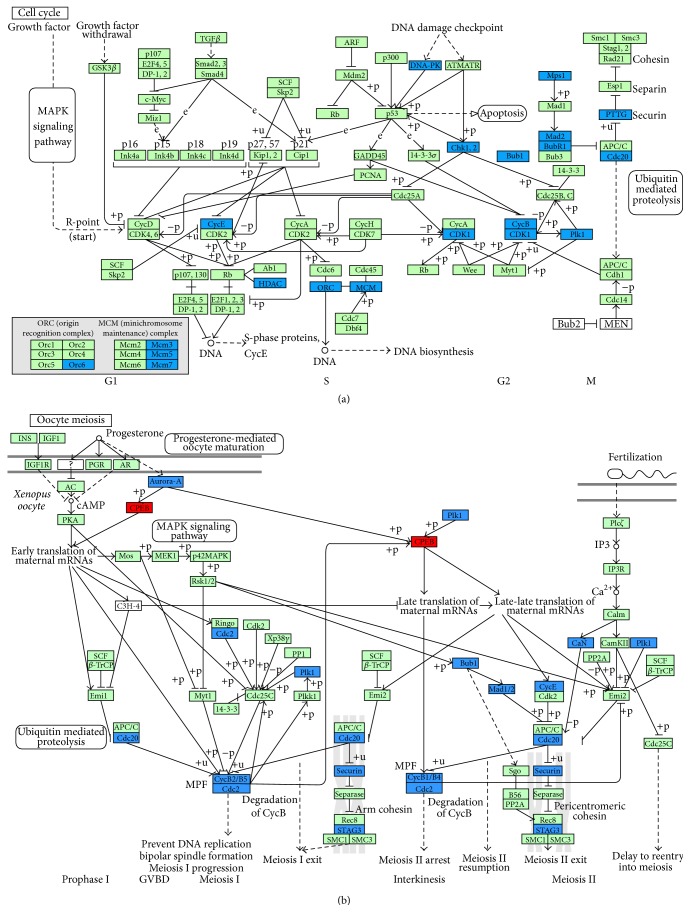
Differentially expressed genes involved in cell cycle (a) and oocyte meiosis (b) pathways. Blue backgrounds indicate genes differentially downregulated and red indicate genes differentially upregulated in old* M. mulatta* ovaries.

Human-*M. mulatta* orthologs were used to select the differentially expressed genes, and 476 annotated genes, 51 upregulated and 425 downregulated, were identified (Table S4). Analysis with DAVID Bioinformatics Resources 6.7 [38] showed that the differentially expressed genes were mainly enriched in the following seven KEGG pathways: “cell cycle,” “oocyte meiosis,” “progesterone-mediated oocyte maturation,” “p53 signaling pathway,” “DNA replication,” “mismatch repair,” and “nucleotide excision repair,” and all genes involved were downregulated in the old ovaries (Table 4). It is well known that aging is associated with both a decrease in the efficiency of repair and an accumulation of DNA damage [52, 53]. In our results, the genes involved in DNA replication and repair pathways were all downregulated in old monkey ovaries (Figure 5, Tables 4 and 5), which might indicate cellular senescence, caused by accumulation of damage to nuclear and mitochondrial DNA, decline in the function of stress resistance, which might lead to apoptosis and immune response [30, 44, 54]. In cell cycle and oocyte meiosis pathways, the differentially expressed genes involved were all downregulated in old ovaries except for the* Cpebs* gene (Figure 6), which reflected the lower control ability of cell proliferation and oocyte maturation. It has been reported that* Cpebs* have the function of balancing between senescence and proliferation depending on translational repression and/or activation, which can be regulated by progesterone. With the lack of progesterone in old animals, the function of* Cpebs* trends toward translational repression [55]. The p53 signaling pathway is intimately involved in aging and apoptosis according to a previous report [56]. A few popular mechanisms related to aging are listed in Table 5 by analyzing our microarray data, which shares some similarities to the previous reports in mice [25, 26].

In order to identify pronounced, age-related changes in gene expression in the ovary, we conducted further functional annotation focused on* GOTERM_BP_FAT* and* KEGG_PARTHWAY* of 51 orthologs genes upregulated in old versus young ovary and 58 orthologs genes downregulated in old versus young and middle-aged ovary in old ovary (Table S4). We found that the functions of upregulated genes were enriched mainly in negative regulation of cellular biological processes, aging, defense response, oxidation/reduction, cell growth, and KEGG pathways of “leukocyte transendothelial migration” and “biosynthesis of unsaturated fatty acids” (Table 6). The functions of downregulated genes were enriched primarily in cell cycle, reproduction, chromatin organization and regulation of transcription, DNA and RNA process, methylation, and KEGG pathways of “cell cycle,” “oocyte meiosis,” “progesterone-mediated oocyte maturation,” and “spliceosome” (Table 7). These data indicate that ovarian aging is accompanied with the increases in defense and immune responses and oxidation reduction, the decreases in capacity for reproduction, cell division, DNA replication and repair, and some changes of epigenetic regulation. These findings will be helpful for tracking biomarkers and understanding mechanisms of ovary aging in primates.

## 4. Conclusion

Our results demonstrate substantial differences in ovarian gene expression between old and middle-aged or young rhesus monkeys. These differences exist at the level of transcription of genes involved in critical biological functions and pathways in the ovary/oocytes, which probably affect aging process through DNA damage, mitochondrial dysfunction, oxidative stress and immune response, and epigenetics. Thus, age-specific gene expression profiling can provide information on processes that may be related to ovarian/oocyte aging and possibly reveal a contribution of altered gene expression to decreased fertility.

## Supplementary Material

In the Supplementary Material, Table S1 shows the genes differentially expressed in ovaries of old rhesus monkeys versus that of young and middle-aged monkeys. Table S2 shows the GO terms of genes differentially expressed in old versus young and middle-aged monkey ovaries analyzed by MAS3.0 online software. Table S3 shows the KEGG pathways of genes differentially expressed in old monkey ovaries as a function of age, analyzed by MAS3.0 online software. Table S4 shows the human-M mulatta orthologous genes retrieved by Bio-Mart online software.

## Figures and Tables

**Table 1:** Primers used for qRT-PCR.

RefSeq transcript ID	Gene symbol	Sequence (5′ to 3′)	Amplicon size (bp)
XM_001096328	*MRAP*	S: GCTGCTCCTCTTCCTCATCC A: TCAGTTCTGCTCCCTGGCTC	204

XM_001104871	*MMP9*	S: AGTCCACCCTTGTGCTCTTC A: CTGCCACCCGAGTGTAAC	103

NM_001042638	*THY1*	S: CCTGACCCGTGAGACAAAG A: GGTGAAGGCGGATAAGTAGAG	127

XM_001109859	*LOC717872*	S: GTGAACGGTCGCCTGTATC A: AAAACTGGGGTCCTTGAGC	241

XM_001097914	*IGFBP4*	S: AATTCGAGACCGGAGCAC A: GGATGGGAATGATGTAGAGGT	170

XM_001103253	*NASP*	S: GGGCTTGGCTTATGGGT A: ATCTCGGGTAGCAGTTCCTT	177

XM_001085259	*PTTG1*	S: GCTTTGGGAACCGTCAAC A: TTCTGGATAGGCATCGTCTG	153

XR_011039	*AURKA*	S: TCTGTGGCACCCTGGACTA A: AGGAGGCTTCCCAACTAAAA	119

XM_001085850	*BCL2L10*	S: GTGACAGCCTGGTGGAAGA A: AAGCCTGGATCAGCAGTTTT	220

XM_001084147	*BARD1*	S: GAAAGCCCAAGCCAGACA A: ACTTTGCCCTGCCGAAC	151

XM_001101192	*TACC3*	S: ATGTGCCACCCAAGAACG A: AGCCAAAGGAGCCTCAAGT	122

XM_001095416	*THBD*	S: TGTGAGCACTTCTGCGTTCC A: CCAGGTCGTAGCCAGGTTT	199

XM_001083479	*DNMT3A*	S: GGTTTACCCACCTGTCCCA A: CACCTGAATGCCCAAGTCC	109

XM_001114760	*ZP3*	S: GCACTCCAAGCCATTCCA A: GGCCCACTGCTCTACTTCATA	158

XR_010378	*LOC703074*	S: GAACTCTTCAAGCGTGTCTCA A: CCAGGTCGTTCATGTTGCT	130

XM_001087511	*WASF1*	S: CTTTCTGCCTTGCCATTTAG A: AGGTGGGTATCGGTTTCG	133

XR_011694	*LOC707199*	S: GACCTGAAGGACCCGTTTG A: CAGGAGGAAGTTGTGGGAGAT	132

XM_001106702	*MCM3*	S: AAGATGGGGATTCATACGACC A: GCCTTCAACCTGGATTCACT	139

XM_001098017	*IGF2BP3*	S: ATCTGAACGCCTTGGGTC A: TTGCTCAAACTGCGGGTA	101

XM_001105684	*XRCC6*	S: GCGAGCACTCAGCAGGTTA A: GTCTTGGTTTTCACTGGTTCAT	141

XM_001094077	*HELLS*	S: ACTTCCTAACTGGATGGCTGAG A: GCTGTAACGCATTTCGGTCT	188

XM_001095697	*CDK1*	S: CCTAGCATCCCACGTCAAA A: ATGATTCAGTGCCATTTTGC	109

XM_001093457	*FGF14*	S: CCAAGATCCCCAGCTCAA A: TGGCAACAACACGCAGTC	158

XM_001104061	*UBE2C*	S: TATGCCTGGACATCCTGAAG A: GGGACTATCAATGTTGGGTTC	104

XM_001093770	*THBS1*	S: GCCAGGGCGTCGAATAT A: TGCCATTGCCAGCGTAG	168

NM_001033084.1	*ACTB*	S: GCCCTGAGGCTCTCTTCCA A: CGGATGTCCACGTCACACTT	100

*ACTB* served as internal control. S: sense; A: antisense.

**Table 2:** Comparison analysis of follicles numbers in different aged monkey ovaries.

Groups	Number of ovaries counted	Number of sections counted per ovary	Number of healthy follicles per ovary per animal (mean ± SEM)	Number of atretic follicles per ovary per animal (mean ± SEM)^*∗*^	Total follicles per ovary per animal (mean ± SEM)
Young	3	31	22097 ± 1243.0^A^	400.7 ± 21.4^A^	22498 ± 1264.0^A^
Middle-aged	3	33	12581 ± 512.7^B^	534.7 ± 39.1^B^	13115 ± 499.4^B^
Old	3	20	7.3 ± 2.3^C^	2.7 ± 1.1^C^	10.0 ± 1.3^C^

^*∗*^Atretic follicles with no obvious oocytes were not counted. A, B, and C in each column indicate significant differences among groups (*P* < 0.001).

**Table 3:** qRT-PCR validation of microarray data by using the data-generated RNA samples.

Gene symbol	Microarray ratio		qRT-PCR ratio				
	O versus M	O versus Y	O versus M	*t*-test (*P* value)	O versus Y	*t*-test (*P* value)	RefSeq transcript ID
Upregulated genes							
*MRAP*	—	22.8616	19.1597	0.030^*∗*^	45.2548	0.027^*∗*^	XM_001096328
*MMP9*	—	5.2734	14.4765	0.815	4.2191	0.002^*∗∗*^	XM_001104871
*THY1*	—	2.8757	1.3062	0.273	1.6950	0.098	NM_001042638
*LOC717872*	—	7.0041	1.3145	0.054	3.3168	0.073	XM_001109859
*IGFBP4*	—	2.2457	0.8946	0.417	1.4958	0.403	XM_001097914
Downregulated genes							
*NASP*	0.4166	0.3654	0.2207	0.0001^*∗∗*^	0.2588	0.0001^*∗∗*^	XM_001103253
*PTTG1*	0.2017	0.1803	0.2505	0.045^*∗*^	0.2811	0.002^*∗∗*^	XM_001085022
*AURKA*	0.3774	0.2893	0.3221	0.043^*∗*^	0.1231	0.002^*∗∗*^	XR_011039
*BCL2L10*	0.0369	0.0279	0.0000	0.001^*∗∗*^	0.0000	0.032^*∗*^	XM_001085850
*BARD1*	0.3524	0.2966	0.5953	0.193	0.2293	0.015^*∗*^	XM_001084147
*TACC3*	0.1866	0.1238	0.1756	0.004^*∗∗*^	0.0884	0.004^*∗∗*^	XM_001101192
*THBD*	0.4251	0.4500	3.5801	0.160	3.8106	0.845	XM_001095416
*DNMT3A*	0.3844	0.3167	0.1805	0.0004^*∗∗*^	0.0634	0.0001^*∗∗*^	XM_001083234
*ZP3*	0.0804	0.0347	0.0005	0.020^*∗*^	0.0003	0.0002^*∗∗*^	XM_001114760
*LOC703074*	0.0294	0.0265	0.0010	0.001^*∗∗*^	0.0012	0.001^*∗∗*^	XR_010378
*WASF1*	0.2458	0.2101	0.3482	0.0001^*∗∗*^	0.1213	0.0001^*∗∗*^	XM_001087511
*LOC707199*	0.4462	0.3053	0.7749	0.435	0.2752	0.041^*∗*^	XR_011694
*MCM3*	0.2856	0.212	0.3024	0.0001^*∗∗*^	0.0734	0.002^*∗∗*^	XM_001106702
*IGF2BP3*	0.1042	0.0617	0.2369	0.092	0.0993	0.024^*∗*^	XM_001098017
*FGF14*	0.1707	0.1074	0.2601	0.005^*∗∗*^	0.0696	0.002^*∗∗*^	XM_001093457
*XRCC6*	—	0.4987	0.8063	0.855	0.5597	0.146	XM_001105684
*HELLS*	—	0.2874	0.227	0.0001^*∗∗*^	0.1330	0.0005^*∗∗*^	XM_001094077
*CDK1*	—	0.0942	0.1716	0.0001^*∗∗*^	0.1137	0.0005^*∗∗*^	XM_001095697
*UBE2C*	—	0.1019	0.3849	0.016^*∗*^	0.1806	0.001^*∗∗*^	XM_001104061
*THBS1*	0.3518	—	0.3748	0.001^*∗∗*^	0.1309	0.069	XM_001093770

O: old group; M: middle-aged group; Y: young group; —: no significant difference in gene expression; ^*∗*^ *P* < 0.05; ^*∗∗*^ *P* < 0.01.

**Table 4:** KEGG pathway analysis with the orthologs genes conserved between human and *M. mulatta*.

KEGG pathway	Genes
Cell cycle	*CCNE1, CHEK1, BUB1, HDAC2, TTK, CCNB1, PTTG1, CDC20, CDK1, BUB1B, MCM5, ORC6L, MCM3, MCM7, MAD2L1, PLK1, *and *CCNB2*

Oocyte meiosis	*CDK1, CCNE1, PPP3CB, BUB1, CCNB1, PTTG1, MAD2L1, PLK1, STAG3, CDC20, CCNB2, *and *AURKA*

Progesterone-mediated oocyte maturation	*CDK1, HSPCA, BUB1, CCNB1, MAD2L1, PLK1, *and *CCNB2*

p53 signaling pathway	*CDK1, CCNE1, CHEK1, CCNB1, CCNB2, *and *BID*

DNA replication	*RFC3, MCM5, RFC4, POLD1, POLD3, FEN1, MCM3, *and *MCM7*

Mismatch repair	*RFC3, RFC4, POLD1, POLD3, *and *MSH2*

Nucleotide excision repair	*RFC3, RFC4, POLD1, *and *POLD3*

**Table 5:** Analysis of differentially expressed orthologs genes with popular mechanisms of aging.

Mechanisms	Genes involved
Mitochondrion	*ALDH18A1, ALDOC* ^*∗*^ *, ARG2, BCL2L10, BID, CAPRIN2, CYP11A1* ^*∗*^ * DSP, FAM136A, FEN1, GATM, ILF3, LACTB2* ^*∗*^ * MTIF3, NADKD1* ^*∗*^ *, P4HA1, PPP3CB, RARS2, SHMT1, TOMM34, TPP1* ^*∗*^, and *USP30 *

Oxidation reduction and electron transport	*ALDH18A1, AOX1* ^*∗*^ *, CYP11A1* ^*∗*^ *, FADS1* ^*∗*^ *, HSD17B1, KDM1A, LDHAL6A, NARF, NELL1, P4HA1, *and *RETSAT* ^*∗*^

Apoptosis	*ALDOC* ^*∗*^ *, ARHGEF7, AVEN, BARD1, BCL2L10, BCLAF1, BID, BRCA1, BUB1B, CASP2, CDK1, CSE1L, DEDD, F2, GAL, HELLS, KRT8, MAEL, MMP9* ^*∗*^ *, MSH2, NELL1, NPM1, SOX4, TIA1, TUBB, *and *TUBB2C*

Immune system	*AOX1* ^*∗*^ *, BCL11A, CCNB2, CHD7, CHIT1* ^*∗*^ *, CLEC1A* ^*∗*^ *, F2, GAL, GLMN, IGFBP4* ^*∗*^ *, IGHE, IL1R1* ^*∗*^ *, ILF2, JARID2, MASP1* ^*∗*^ *, MMP9* ^*∗*^ *, MSH2, OPRK1, PPP3CB, SOX4, TACC3, TCF3, THY1* ^*∗*^ *, TUBB, TUBB2C,* and* XRCC6*

DNA replication and repair	*ASF1A, BARD1, BRCA1, CCNO, CHAF1A, CHEK1, DKC1, DTL, FANCE, FANCI, FEN1, GINS2, HMGA1, MCM10, MCM3, MCM5, MCM7, MORF4L1, MSH2, NASP, NEIL3, ORC6L, PAPD7, PARP1, POLD1, POLD3, PTTG1, RAD50, RAD51, RAD51C, RBM4, RFC3, RFC4, RMI2, RUVBL2, SFPQ, SSRP1, TDP1, TRIP13, TYMS, *and *XRCC6*

Methylation	*DHX9, DNMT3A, DNMT3B, FBL, FUS, HELLS, HMGA1, HNRNPD, HNRNPK, HNRNPU, HNRPDL, ILF3, MAEL, PABPC4, PRMT5, PRMT7, RAP1A* ^*∗*^ *, RASL10A, RHOQ* ^*∗*^ *, RRAS2, SFPQ, SRSF1, SUV39H2, TDRD1, TGS1, THOC4, TUBA1B, *and *TUBB*

Reproduction	*AMH, BCL2L10, CCNB1, CCNE1, CELSR2, CEP57, CHD7, CHEK1, DAZAP1, DEDD, DNMT3A, FIGLA, HIST1H1A, HSF2BP, LHX8, MAEL, MBD2, MSH2, NLRP14, NPM2, OOEP, PPAP2B* ^*∗*^ *, PRMT7, PTTG1, RAD51C, RPL39L, STRBP, TDRD1, TRIP13, ZP2, ZP3, *and *ZP4*

Aging	*NPM1, ENG* ^*∗*^ *, MSH2, FADS1* ^*∗*^, and *ALDOC* ^*∗*^

Telomere	*DKC1, PARP1, XRCC6, *and *RAD50*

^*∗*^Orthologs genes upregulated in old monkey ovaries.

**Table 6:** Results of functional annotation of orthologs genes upregulated in old monkey ovary compared to young monkey ovary.

Category	Term	Genes
Regulation of cellular process	Negative regulation of catalytic activity/negative regulation of molecular function	*PKIG, CAST, ENG, APOC1, *and *THY1*
	Negative regulation of cellular component organization	*RHOQ, APOC1, *and *THY1*
	Negative regulation of nitrogen compound metabolic process/negative regulation of cellular biosynthetic process/negative regulation of biosynthetic process	*PKIG, ENG, PBXIP1, APOC1, *and *CCDC85B*

Aging	Aging	*ENG*, *FADS1*, and *ALDOC*

Defense response	Response to wounding	*IGFBP4*, *AOX1*, *ENG*, *TFPI*, and *MASP1*
	Defense response	*CLEC1A*, *IGFBP4*, *AOX*1, *IL1R1*, and *MASP1*
	Response to endogenous stimulus	*CYP11A1*, *RHOQ, FADS1, *and *ALDOC*

Oxidation reduction	Oxidation reduction/fat-soluble vitamin metabolic process/unsaturated fatty acid biosynthetic process	*CYP11A1*, *AOX1*, *RETSAT*, *FADS1*, and *FADS2*

Cell growth	Regulation of cell growth	*IGFBP4,WISP2*, and *CCDC85B*

KEGG_PATHWAY	Leukocyte transendothelial migration	*MMP9*, *RAP1A*, *MYL9*, and *THY1*
	Biosynthesis of unsaturated fatty acids	*FADS1*, *FADS2*

Functional categories of genes were assembled from annotation and PubMed.

**Table 7:** Results of functional annotation of orthologs genes downregulated in old monkey ovary compared to young and middle-aged monkey ovary.

Category	Term	Genes
Cell cycle	Cell cycle/mitosis/mitotic cell cycle/nuclear division/cell division/organelle fission	*BARD1*, *RAD51*, *CDCA2*, *BUB1*, *TTK*, *CCNB1*, *PTTG1*, *KIF15*, *SUV39H2*, *NCAPH*, *NCAPG*, *MCM3*, *TACC3*, *OIP5*, *CCNB2*, and *AURKA*

Cellular macromolecules	Macromolecular complex assembly/macromolecular complex subunit organization/protein complex biogenesis/assembly	*TGS1, RAD51, TUBB2C, DNAAF2, ASF1A, OOEP, HIST1H1D, *and *ENSG00000112290*

Reproduction	Sexual reproduction/reproductive cellular process/gamete generation/oocyte development/fertilization	*FIGLA*, *ZP3*, *BCL2L10*, *CCNB1*, *OOEP*, *DNMT3A*, *PTTG1*, *ZP2*, and *AMH*

Chromatin	Chromosome organization/chromatin assembly or disassembly/chromatin organization	*SUV39H2*, *NCAPH*, *WHSC1*, *NCAPG,ASF1A*, *DNMT3A*, *PTTG1*, *HIST1H1D*, and *DNMT3B*

Regulation of cellular process	Positive regulation of macromolecule metabolic process/positive regulation of nitrogen compound metabolic process	*BARD1, RAD51, TESC, ASF1A, TTK, *and *CCNB1*
	Regulation of transcription	*FUBP1, OTX2, FIGLA, TGS1, MLF1IP, TESC, ASF1A, ZNF77, SUV39H2, HNRNPD, ELAVL2, DNMT3A, *and *MCM3*
	Regulation of apoptosis or cell death	*BARD1, TUBB2C, *and *BCL2L10*

DNA	DNA metabolic process	*RMI2*, *BARD1*, *RAD51*, *GINS2*, *ASF1A*, *DNMT3A*, *MCM3*, and *PTTG1*
	DNA repair/response to DNA damage stimulus	*BARD1, RAD51, ASF1A, *and *PTTG1*

RNA	RNA splicing/mRNA processing/mRNA metabolic process	*SRSF2*, *TGS1*, *HNRNPD*, *HNRNPA1L2*, and *ENSG00000135486*

Methylation	Biopolymer methylation/methylation	*SUV39H2*, *TGS1*, *DNMT3A*, and *DNMT3B*

KEGG_PATHWAY	Cell cycle	*BUB1*, *TTK*, *CCNB1*, *MCM3*, *PTTG1*, and *CCNB2*
	Oocyte meiosis	*BUB1*, *CCNB1*, *PTTG1*, *CCNB2*, and *AURKA*
	Progesterone-mediated oocyte maturation	*BUB1*, *CCNB1*, and *CCNB2*
	Spliceosome	*SRSF2*, *HNRNPA1L2, *and *ENSG00000135486*

Functional categories of genes were assembled from annotation and PubMed.
