# Supplementary material for: Age-Specific Gene Expression Profiles of Rhesus Monkey Ovaries Detected by Microarray Analysis
Source: Biomed Res Int. 2015 Sep 2;2015:625192. doi: 10.1155/2015/625192 (PMC4571527; doi:10.1155/2015/625192)
Supplement: Supplementary file 1 — In the Supplementary Material, Table S1 shows the genes differentially expressed in ovaries of old rhesus monkeys versus that of young and middle-aged monkeys. Table S2 shows the GO terms of genes differentially expressed in old versus young and middle-aged monkey ovaries analyzed by MAS3.0 online software. Table S3 shows the KEGG pathways of genes differentially expressed in old monkey ovaries as a function of age, analyzed by MAS3.0 online software. Table S4 shows the human-M mulatta orthologous genes retrieved by Bio-Mart online software. [file 625192.f1.pdf]

Supplemental Table S1. Genes differentially expressed in ovary of old rhesus monkeys vs. young and middle-age monkeys

Gene Name	Gene Symbol	Old vs. Young q-value* (%)	Old vs. Young Fold Change	Old vs. Young Differentially expressed	Old vs. Middle-age q-value (%)	Old vs. Middle-age Fold Change	Old vs. Middle-age Differentially expressed	RefSeq Transcript ID
Similar to Cofilin-1	*LOC721819*	4.495104	26.8915	+	-	-	-	XR_014660
melanocortin 2 rece	*MRAP*	4.646275	22.8616	+	-	-	-	XM_001096562 /// XM_001096665 /// XM_001096774
chitinase 1 (chitotri	*CHIT1*	3.396112	12.2749	+	-	-	-	XM_001102929 /// XM_001103012
chromosome 4 ope	*MGARP*	4.7194	7.4128	+	-	-	-	XM_001087713
similar to WNT1-in	*LOC717872*	3.431917	7.0041	+	-	-	-	XM_001109859
potassium voltage-	*KCNB1*	1.590142	5.8293	+	-	-	-	XM_001098678
Similar to peptidylp	*LOC707078*	2.453362	5.2929	+	-	-	-	XR_011674
matrix metallopepti	*MMP9*	2.735892	5.2734	+	-	-	-	XM_001104871
similar to mannan-l	*LOC708206*	4.257852	5.1997	+	-	-	-	XR_012871
Similar to profilin 1	*LOC706963*	4.257852	5.0668	+	-	-	-	XR_011653
similar to acyl-CoA	*LOC694871*	4.495104	4.5633	+	-	-	-	XM_001083742 /// XM_001083847 /// XM_001083961 /// XM_001084069 /// XM_001084192 /// XM_001084305
Hypothetical protein	*LOC701880*	4.257852	4.5056	+	-	-	-	XM_001090162
aldolase C, fructos	*ALDOC*	4.257852	4.3805	+	-	-	-	XM_001107579 /// XM_001107637
metallothionein 2A	*MT2A*	4.495104	4.2412	+	-	-	-	XM_001092096
similar to dedicator	*LOC698081*	4.257852	4.2226	+	-	-	-	XR_011087
transmembrane pro	*TMEFF2*	4.495104	3.8991	+	-	-	-	XM_001084739 /// XM_001084859
similar to fatty acid	*LOC719037*	4.495104	3.7342	+	-	-	-	XM_001116637
similar to phosphat	*LOC715993*	4.646275	3.5695	+	-	-	-	XM_001114350 /// XM_001114366
small-conductance	*SK3*	2.735892	3.5035	+	-	-	-	XM_001112021
heat shock 22kDa		*HSPB8*	4.495104	3.4527	+	-	-	-	NM_001032937
Similar to Cadherin	*LOC702800*	4.257852	3.3358	+	-	-	-	XR_011069
copine VIII	*CPNE8*	4.495104	3.1142	+	-	-	-	XM_001087185
C-type lectin domai	*CLEC1A*	4.495104	2.9213	+	-	-	-	XM_001114932
Thy-1 cell surface a	*THY1*	4.495104	2.8757	+	-	-	-	XM_001105767 /// XM_001105824 /// XM_001105894

similar to aspartate	*LOC699884*	0	2.8073	+	-	-	-	XM_001090473 /// XM_001090590 /// XM_001090710 /// XM_001090824
Hypothetical protei	*LOC716818*	4.495104	2.8029	+	-	-	-	XM_001107783
Similar to phosphol	*LOC718418*	2.735892	2.5773	+	-	-	-	XR_014465
similar to family wit	*LOC718937*	4.495104	2.5589	+	-	-	-	XM_001116963
similar to ras-like p	*LOC717769*	4.495104	2.5486	+	-	-	-	XM_001109631
tissue factor pathw	*TFPI*	4.646275	2.5296	+	-	-	-	XM_001104806
similar to Myosin re	*LOC709784*	4.222205	2.5159	+	-	-	-	XR_012098
Butyrylcholinestera	*BCHE*	4.756981	2.4292	+	-	-	-	XR_011736
Similar to TBC1 (tre	*LOC697357*	4.495104	2.2916	+	-	-	-	XM_001087581
insulin-like growth f	*IGFBP4*	4.495104	2.2457	+	-	-	-	XM_001097914
similar to cysteine s	*LOC701290*	4.257852	2.2242	+	-	-	-	XM_001102580 /// XM_001102666 /// XM_001102752 /// XM_001102845
similar to protein ty	*LOC703937*	4.7194	2.1583	+	-	-	-	XR_011208
Similar to PML-RAI	*LOC712017*	4.495104	2.1566	+	-	-	-	XR_012548
hyaluronoglucosam	*HYAL1*	4.495104	2.0921	+	-	-	-	XM_001101963 /// XM_001102052 /// XM_001102143 /// XM_001102231 /// XM_001102322 /// XM_001102414
Rho guanine nucle	*ARHGEF12*	4.257852	1.9327	+	-	-	-	XR_012563
coatomer protein c	*COPZ2*	4.646275	1.9702	+	-	-	-	XM_001084942
similar to cytochror	*LOC696520*	4.7194	1.9159	+	-	-	-	XM_001088961
chromosome 5 ope	*NADKD1*	3.010359	1.8107	+	-	-	-	XM_001092624
similar to pre-B-cell	*LOC717036*	2.735892	1.5683	+	-	-	-	XM_001114600 /// XM_001114624 /// XM_001114649
RAP1A, member o	*RAP1A*	4.7194	1.5621	+	-	-	-	XM_001106027
cell cycle progressi	*CCPG1*	4.222205	1.998	+	-	-	-	XM_001087398
Similar to ring finge	*LOC707577*	4.495104	1.9885	+	-	-	-	XR_012891
similar to Epithelial	*LOC717867*	4.222205	1.9722	+	-	-	-	XM_001113524
similar to PDZ dom	*LOC709901*	4.7194	1.7813	+	-	-	-	XM_001098466
similar to cAMP-de	*LOC712474*	3.431917	1.7564	+	-	-	-	XM_001109878 /// XM_001109925 /// XM_001109963 /// XM_001110008 /// XM_001110061
ring finger protein 1	*RNF13*	4.495104	1.8883	+	-	-	-	XM_001108379
hypothetical proteir	*LOC710818*	4.7194	1.9368	+	-	-	-	XR_012295
Similar to peptidase	*LOC714817*	4.495104	1.6799	+	-	-	-	XR_013065

hypothetical proteir	*LOC717131*	4.495104	1.7822	+	-	-	-	XM_001108353
similar to Endoglin	*ENG*	4.646275	1.9318	+	-	-	-	XR_011598
septin 11	*SEPT11*	4.495104	1.9907	+	-	-	-	XM_001093263 /// XM_001093370
similar to meningio	*LOC713280*	4.495104	1.995	+	-	-	-	XR_012573
similar to Homeobc	*LOC710591*	4.495104	1.799	+	-	-	-	XM_001106266 /// XM_001106332
similar to activating	*LOC719150*	4.646275	1.5342	+	-	-	-	XM_001115871
coiled-coil domain (	*CCDC85B*	4.495104	1.5389	+	-	-	-	XM_001112215
similar to Kruppel-li	*KLF7*	4.495104	1.8212	+	-	-	-	XM_001106942 /// XM_001107009 /// XM_001107070 /// XM_001107137
X-ray repair comple	*XRCC6*	2.46188	0.4987	+	-	-	-	XM_001105684
SWI/SNF-related m	*SMARCC1*	1.022234	0.4967	+	-	-	-	XR_012360
hypothetical proteir	*LOC711168*	0.526605	0.4966	+	-	-	-	XM_001107514 /// XM_001107572 /// XM_001107633
similar to Heteroge	*HNRPA1*	3.010359	0.4954	+	-	-	-	NM_001042783 /// XM_001109413 /// XR_009653 /// XR_009774 /// XR_010989 /// XR_011222 /// XR_011984 /// XR_012371 /// XR_013205 /// XR_013276 /// XR_013977 /// XR_014497
similar to jumonji, A	*LOC702291*	1.362979	0.4946	+	-	-	-	XR_011465
arginyl-tRNA synth	*RARS2*	3.181323	0.4936	+	-	-	-	XM_001092575
similar to CG15120	*LOC715522*	0	0.4913	+	-	-	-	XM_001103696 /// XM_001103783 /// XM_001103954 /// XM_001104045 /// XM_001104130 /// XM_001104213 /// XM_001104292
general transcriptio	*GTF3C4*	4.114412	0.4909	+	-	-	-	XM_001104736
similar to tripartite r	*LOC711397*	2.46188	0.4906	+	-	-	-	XM_001113088 /// XM_001113121 /// XM_001113153
THAP domain cont	*THAP9*	2.92445	0.4888	+	-	-	-	XM_001086766
downstream neight	*DONSON*	4.495104	0.4847	+	-	-	-	XM_001090910 /// XM_001091031
similar to exonucle	*LOC696366*	4.048115	0.4793	+	-	-	-	XM_001088290 /// XM_001088611 /// XM_001088716 /// XM_001088838 /// XM_001088943 /// XM_001089058
similar to TBP-assc	*LOC704624*	3.495013	0.4747	+	-	-	-	XR_012571
hypothetical proteir	*LOC695614 /// L*	1.775852	0.4734	+	-	-	-	XR_010359 /// XR_012282 /// XR_013032
similar to Splicing f	*LOC712439*	2.273754	0.4701	+	-	-	-	XR_012628
similar to Protein C	*C3H21orf59*	4.114412	0.4672	+	-	-	-	XM_001095114 /// XM_001095455 /// XM_001095667 /// XM_001095768 /// XM_001095882
similar to polyamin	*LOC711508*	2.46188	0.4669	+	-	-	-	XM_001105501 /// XM_001105574
melanoma antigen	*MAGED4B*	3.495013	0.4641	+	-	-	-	XM_001085892

similar to COP9 co	*LOC712697*	4.7194	0.4633	+	-	-	-	XM_001113695 /// XM_001113722 /// XM_001113748 /// XM_001113774 /// XM_001113795 /// XM_001113822 /// XM_001113847 /// XM_001113876 /// XM_001113901 /// XM_001113929 /// XM_001113954 /// XM_001113976
similar to Nucleopla	*LOC712300*	2.044468	0.4625	+	-	-	-	XM_001111044
similar to transloca	*LOC711965*	2.92445	0.461	+	-	-	-	XR_013721
melanoma antigen	*MAGED2*	1.775852	0.4596	+	-	-	-	XM_001090720 /// XM_001091068 /// XM_001091197 /// XM_001091315 /// XM_001091438 /// XM_001091552
RNA binding motif	*RBMX2*	3.495013	0.4559	+	-	-	-	XM_001093627
hypothetical proteir	*LOC709979*	2.92445	0.4535	+	-	-	-	XR_012341
Secretory carrier m	*SCAMP2*	1.775852	0.4523	+	-	-	-	XM_001098273
similar to related R	*LOC699700*	3.907825	0.4482	+	-	-	-	XM_001093122
spermatid perinucle	*STRBP*	3.095314	0.4466	+	-	-	-	XM_001084032 /// XM_001084266 /// XM_001084386 /// XM_001084509 /// XM_001084632
similar to zinc finge	*LOC721492*	3.907825	0.4447	+	-	-	-	XM_001117659
zinc finger protein 2	*ZNF219*	2.46188	0.4427	+	-	-	-	XM_001095832
similar to Protein C	*LOC694192*	2.775191	0.4426	+	-	-	-	XM_001082497 /// XM_001082628 /// XM_001082761
similar to Cornichor	*LOC701416*	4.222205	0.4417	+	-	-	-	XM_001095074 /// XM_001095178
hypothetical proteir	*LOC721604*	0	0.438	+	-	-	-	XR_014635
similar to Transcrip	*LOC721325*	3.181323	0.435	+	-	-	-	XR_014602
similar to GDNF fa	*LOC714050*	3.095314	0.4345	+	-	-	-	XM_001112599 /// XM_001112630
hypothetical proteir	*LOC699208*	4.7194	0.4338	+	-	-	-	XM_001089726 /// XM_001089845
UDP-Gal:betaGlcN	*B4GALT4*	4.048115	0.4335	+	-	-	-	XM_001108377 /// XM_001108435 /// XM_001108497 /// XM_001108549 /// XM_001108604 /// XM_001108651 /// XM_001108706
coiled-coil domain	*CCDC99*	3.181323	0.4334	+	-	-	-	XM_001091652 /// XM_001091769
regulator of G-prote	*RGS7*	4.222205	0.4305	+	-	-	-	XM_001095075 /// XM_001095180
hypothetical proteir	*LOC703861*	2.46188	0.4291	+	-	-	-	XM_001097994
aldehyde dehydrog	*ALDH18A1*	3.251114	0.4264	+	-	-	-	XM_001099359 /// XM_001099461
similar to immunog	*LOC719535*	4.048115	0.4262	+	-	-	-	XM_001117327 /// XM_001117340 /// XM_001117344
similar to fibronecti	*LOC701832*	3.649076	0.4244	+	-	-	-	XR_011739
similar to Claudin-1	*CLDN15*	2.46188	0.4234	+	-	-	-	XR_014238
protein BAP28	*HEATR1*	3.482703	0.422	+	-	-	-	XR_013684

similar to heteroger	*LOC693392*	2.92445	0.4218	+	-	-	-	XR_009874
hypothetical proteir	*LOC713815*	4.114412	0.4209	+	-	-	-	XM_001111926 /// XM_001111964 ///<br>XM_001112002 /// XM_001112038
Similar to kallikrein	*LOC701865*	3.495013	0.4198	+	-	-	-	XR_010946
Treacher Collins-Fr	*TCOF1*	3.095314	0.418	+	-	-	-	NM_001039952
Hypothetical proteir	*LOC708774*	4.756981	0.4167	+	-	-	-	XR_011931
similar to synaptota	*LOC700889*	1.986055	0.4155	+	-	-	-	XR_010792
hypothetical proteir	*LOC719916*	3.396112	0.4151	+	-	-	-	XR_014316
dishevelled associa	*DAAM1*	1.930887	0.4131	+	-	-	-	XM_001093054 /// XM_001093489 ///<br>XM_001093607 /// XM_001093729 ///<br>XM_001093845
TGFB-induced fact	*TGIF1*	2.46188	0.4085	+	-	-	-	XM_001082760 /// XM_001082891 ///<br>XM_001083016 /// XM_001083131 ///<br>XM_001083247 /// XM_001083375 ///<br>XM_001083496 /// XM_001083606 ///<br>XM_001083709
cyclin E1	*CCNE1*	3.907825	0.4073	+	-	-	-	XM_001084880 /// XM_001084995
similar to high mob	*LOC718536*	2.775191	0.4049	+	-	-	-	XM_001116392 /// XM_001116397 ///<br>XM_001116404 /// XM_001116410 ///<br>XM_001116415
cyclin J	*CCNJ*	1.579816	0.4041	+	-	-	-	XM_001092632
glomulin, FKBP ass	*GLMN*	2.775191	0.3998	+	-	-	-	XM_001099081
Fanconi anemia, cc	*FANCI*	3.181323	0.3976	+	-	-	-	XM_001092590 /// XM_001092826 ///<br>XM_001093053
similar to calmoduli	*LOC721823*	0	0.3945	+	-	-	-	XM_001118018
similar to RAS and	*LOC708296*	4.257852	0.3923	+	-	-	-	XM_001104352
membrane bound (	*MBOAT2*	3.482703	0.3912	+	-	-	-	XM_001083800 /// XM_001083917
apoptosis, caspase	*AVEN*	3.095314	0.3907	+	-	-	-	XM_001085261
zinc finger protein 2	*ZNF226*	0	0.3906	+	-	-	-	XM_001108650 /// XM_001108756 ///<br>XM_001108801
Similar to Protein a	*LOC708356*	2.044468	0.3896	+	-	-	-	XM_001096827
DNA (cytosine-5-)-r	*DNMT3B*	2.92445	0.388	+	-	-	-	XM_001107249 /// XM_001107376 ///<br>XM_001107440
hypothetical proteir	*LOC695033*	4.7194	0.3868	+	-	-	-	XR_009953
similar to tubulin al	*LOC710110*	2.92445	0.3844	+	-	-	-	XR_013691
similar to SCL/TAL	*LOC710099*	4.257852	0.3828	+	-	-	-	XM_001109403 /// XM_001109488 ///<br>XM_001109536 /// XM_001109592
similar to gastrin-re	*LOC696983*	4.048115	0.3823	+	-	-	-	XM_001086863 /// XM_001086970 ///<br>XM_001087091
chromosome 14 op	*C EP128*	4.048115	0.3823	+	-	-	-	XM_001104541
WDYHV motif cont	*WDYHV1*	4.048115	0.3822	+	-	-	-	XM_001100514
ring finger protein 3	*RNF38*	1.775852	0.3794	+	-	-	-	XM_001082059

Gamma-aminobuty	*GABRA1*	3.095314	0.3793	+	-	-	-	XM_001086287
Similar to Protein K	*LOC708959*	4.495104	0.3786	+	-	-	-	XR_013442
similar to Wee1-like	*WEE1*	2.92445	0.3758	+	-	-	-	XR_012636
cyclin O	*CCNO*	3.095314	0.3732	+	-	-	-	XM_001098228
hypothetical protein	*LOC710539*	3.095314	0.3731	+	-	-	-	XM_001099174
cyclin A	*CCNA2*	3.095314	0.3729	+	-	-	-	XM_001100860
Similar to cyclin K	*LOC716572*	3.095314	0.3713	+	-	-	-	XM_001107367
Similar to non-POU	*LOC697263*	3.649076	0.3705	+	-	-	-	XM_001090372 /// XM_001090945 /// XM_001091069 /// XM_001091198 /// XM_001091316
similar to ring finge	*LOC719403*	4.756981	0.3703	+	-	-	-	XM_001113579
Regulator of calcin	*RCAN2*	2.92445	0.368	+	-	-	-	XM_001101876 /// XM_001101974 /// XM_001102066 /// XM_001102152 /// XM_001102242 /// XM_001102338 /// XM_001102428 /// XM_001102525
Similar to E74-like	*LOC696064*	2.044468	0.3644	+	-	-	-	XR_010093
similar to Tetraspan	*TSPAN6*	4.048115	0.3607	+	-	-	-	XM_001091467
similar to Cadherin	*LOC702800*	1.022234	0.36	+	-	-	-	XR_011069
similar to mucolipin	*LOC710909*	1.022234	0.3547	+	-	-	-	XM_001107980
similar to nucleopla	*LOC715448*	1.986055	0.3523	+	-	-	-	XM_001105673
structural maintena	*SMC4*	3.482703	0.3488	+	-	-	-	XM_001097924 /// XM_001098208 /// XM_001098314 /// XM_001098414 /// XM_001098509 /// XM_001098604 /// XM_001098705 /// XM_001098806 /// XM_001098912 /// XM_001099015 /// XM_001099123 /// XM_001099224 /// XM_001099331 /// XM_001099428
similar to CXXC fin	*LOC693707*	4.495104	0.3488	+	-	-	-	XR_009908
Hypothetical protein	*LOC718666*	3.495013	0.3434	+	-	-	-	XM_001111650
G protein-coupled r	*GPR63*	3.010359	0.3432	+	-	-	-	XM_001100856
similar to CG12772	*LOC703875*	3.482703	0.3395	+	-	-	-	XR_012218
similar to Ras-like p	*RRP22*	2.273754	0.3389	+	-	-	-	XM_001105399 /// XM_001105469
RAB38, member R	*RAB38*	3.495013	0.3371	+	-	-	-	XM_001104269
similar to Midkine p	*MDK*	2.775191	0.3331	+	-	-	-	XR_012413
similar to centrosor	*LOC708753*	3.069357	0.3247	+	-	-	-	XM_001095020
similar to cell divisi	*LOC719808*	3.495013	0.3214	+	-	-	-	XM_001115347 /// XM_001115361
minichromosome m	*MCM10*	4.114412	0.3196	+	-	-	-	XM_001085751
hypothetical protein	*LOC715808*	0	0.3117	+	-	-	-	XM_001112378

minichromosome n	*MCM5*	1.362979	0.3074	+	-	-	-	XR_014169
similar to stromal a	*LOC711345*	4.048115	0.3027	+	-	-	-	XM_001102876
Hypothetical protei	*LOC709633*	4.222205	0.3007	+	-	-	-	XR_013764
similar to Mitotic sp	*LOC708574*	3.495013	0.2998	+	-	-	-	XM_001097054
ubiquitin specific pe	*USP2*	4.222205	0.2988	+	-	-	-	XM_001105267 /// XM_001105333 ///<br>XM_001105412 /// XM_001105484 ///<br>XM_001105553
coagulation factor I	*F2*	2.044468	0.2968	+	-	-	-	NM_001104585 /// XM_001101815 ///<br>XM_001111947
mesothelin	*MSLN*	2.92445	0.2961	+	-	-	-	XM_001087333
similar to ADAM m	*LOC712710*	3.482703	0.2956	+	-	-	-	XR_013898
basonuclin 1	*BNC1*	1.986055	0.2933	+	-	-	-	XM_001111612
Adrenergic, beta-3-	*ADRB3*	2.46188	0.2909	+	-	-	-	NM_001044730 /// XM_001089778
hypothetical protei	*LOC703567*	0	0.2883	+	-	-	-	XM_001101149 /// XM_001101233
wingless-type MMT	*WNT10A*	2.044468	0.2876	+	-	-	-	XM_001095740
helicase, lymphoid-	*HELLS*	3.095314	0.2874	+	-	-	-	XM_001094077 /// XM_001094189 ///<br>XM_001094310 /// XM_001094687 ///<br>XM_001094806 /// XM_001094924 ///<br>XM_001095039 /// XM_001095147 ///<br>XM_001095267 /// XM_001095376 ///<br>XM_001095492 /// XM_001095601 ///
echinoderm microt	*EML4*	2.780477	0.2872	+	-	-	-	XR_013124
Immunoglobulin ka	*IGKC*	1.930887	0.2868	+	-	-	-	XR_011822
Reticulon 4	*RTN4*	3.495013	0.2835	+	-	-	-	XM_001112090
family with sequen	*FAM54A*	3.482703	0.2803	+	-	-	-	XM_001098719 /// XM_001098822
phosphatase and a	*PHACTR3*	2.46188	0.2749	+	-	-	-	XM_001090873 /// XM_001091107
Solute carrier famil	*SLC7A1*	4.646275	0.2653	+	-	-	-	XM_001098500
TPX2, microtubule-	*TPX2*	3.482703	0.2644	+	-	-	-	XM_001109495 /// XM_001109543 ///<br>XM_001109645 /// XM_001109690 ///<br>XM_001109738 /// XM_001109786 ///<br>XM_001109833
SRY (sex determin	*SOX4*	4.222205	0.2617	+	-	-	-	XM_001098923
B-cell translocation	*BTG4*	0	0.2575	+	-	-	-	XM_001105332 /// XM_001105411 ///<br>XM_001105483
Similar to otoferlin i	*LOC696717*	3.482703	0.2571	+	-	-	-	XR_010476
similar to thyroid hc	*LOC709328*	2.775191	0.2554	+	-	-	-	XM_001096221
phosphorylase kina	*PHKA2*	3.069357	0.2544	+	-	-	-	XM_001084454
similar to synaptoja	*LOC705639*	2.92445	0.2506	+	-	-	-	XM_001093995
lamin B1	*LMNB1*	3.251114	0.2472	+	-	-	-	XM_001097237 /// XM_001097340

thymidylate synthet	*TYMS*	1.986055	0.2445	+	-	-	-	XM_001088931 /// XM_001089048 /// XM_001089166 /// XM_001089274 /// XM_001089391
similar to family wit	*LOC699767*	1.579816	0.2438	+	-	-	-	XM_001088222
uroplakin 1B	*UPK1B*	2.92445	0.2425	+	-	-	-	XM_001108219
similar to odd Oz/te	*LOC701138*	1.086124	0.2392	+	-	-	-	XR_010828
nei endonuclease \	*NEIL3*	3.495013	0.2389	+	-	-	-	XM_001090459
aurora kinase C	*AURKC*	1.775852	0.2347	+	-	-	-	XM_001098364
similar to CHK1 ch	*LOC713358*	2.044468	0.2328	+	-	-	-	XM_001111357 /// XM_001111392 /// XM_001111431
similar to chromatir	*LOC721861*	2.780477	0.2318	+	-	-	-	XR_014668
hypothetical proteir	*LOC718812*	4.114412	0.2292	+	-	-	-	XR_014535
serine/threonine/tyr	*STYXL1*	2.780477	0.2291	+	-	-	-	XM_001109465
kinesin family mem	*KIF23*	4.756981	0.2277	+	-	-	-	XM_001086180 /// XM_001086776
glycine amidinotrar	*GATM*	4.756981	0.2228	+	-	-	-	XM_001111486 /// XM_001111522 /// XM_001111561 /// XM_001111604
similar to Protein C	*LOC706590*	2.92445	0.2196	+	-	-	-	XM_001094961
BMP and activin m	*BAMBI*	2.044468	0.2166	+	-	-	-	XM_001106773
ninjurin 2	*NINJ2*	0	0.2139	+	-	-	-	XM_001095186
hypothetical proteir	*LOC718180*	3.495013	0.2103	+	-	-	-	XM_001110520
stanniocalcin 1	*STC1*	2.92445	0.2083	+	-	-	-	XM_001106462 /// XM_001106526
kinesin family mem	*KIF4A*	3.010359	0.2081	+	-	-	-	XM_001084213
ATPase family, AA	*ATAD4*	1.930887	0.2062	+	-	-	-	XM_001084512
similar to IQ motif c	*LOC719575*	4.646275	0.2014	+	-	-	-	XM_001114017
Similar to S. cerevi	*LOC710333*	0	0.2007	+	-	-	-	XR_013519
GNAS complex loc	*GNAS*	3.181323	0.2003	+	-	-	-	XM_001083221 /// XM_001083345 /// XM_001083466 /// XM_001083581 /// XM_001083687 /// XM_001083906 /// XM_001084136
maternal embryoni	*MELK*	3.095314	0.1869	+	-	-	-	XM_001115076
myosin VB	*MYO5B*	2.92445	0.1864	+	-	-	-	XM_001090434 /// XM_001090545 /// XM_001090668
methyl-CpG bindin	*MBD2*	1.412844	0.1858	+	-	-	-	XM_001082249
similar to D4, zinc a	*LOC694878*	2.92445	0.1835	+	-	-	-	XR_010127
Similar to hemicent	*LOC714026*	3.095314	0.1832	+	-	-	-	XR_014149
keratin 8	*KRT8*	1.930887	0.1806	+	-	-	-	XM_001099707
similar to leucine ri	*LOC706386*	2.775191	0.1789	+	-	-	-	XM_001104109 /// XM_001104190
Similar to microtub	*LOC712421*	2.775191	0.1751	+	-	-	-	XR_013783

Hypothetical protei	*LOC713257*	2.306811	0.1739	+	-	-	-	XR_012778
ZW10 interactor	*ZWINT*	2.92445	0.173	+	-	-	-	XM_001098136
v-myc myelocytoma	*MYCN*	4.756981	0.1689	+	-	-	-	XM_001091117 /// XM_001091248 /// XM_001091369
similar to NACHT, I	*LOC708292*	1.022234	0.1594	+	-	-	-	XM_001107454
similar to CDC28 p	*LOC697324*	3.396112	0.1547	+	-	-	-	XM_001089494
3'-phosphoadenosi	*PAPSS2*	2.92445	0.1521	+	-	-	-	XM_001101529 /// XM_001101802 /// XM_001101896 /// XM_001101994
hypothetical protei	*LOC709015*	1.362979	0.1518	+	-	-	-	XM_001098396
similar to PDZ dom	*LOC707432*	3.251114	0.1452	+	-	-	-	XM_001104248
tudor domain conta	*TDRD1*	1.362979	0.1426	+	-	-	-	XM_001091904 /// XM_001092021 /// XM_001092139
similar to B-cell CL	*LOC717675*	2.775191	0.142	+	-	-	-	XR_014354
CAP-GLY domain c	*CLIP1*	4.646275	0.1384	+	-	-	-	XM_001097802 /// XM_001097991 /// XM_001098085 /// XM_001098185 /// XM_001098293
similar to centrome	*LOC709000*	3.482703	0.1347	+	-	-	-	XM_001105956
kinesin family mem	*KIF2C*	2.775191	0.1338	+	-	-	-	XM_001093746
similar to tubulin, a	*LOC707215 /// L*	2.46188	0.127	+	-	-	-	XM_001101935 /// XM_001102025 /// XM_001110230
desmoplakin	*DSP*	0	0.1207	+	-	-	-	XM_001084900 /// XM_001085012
similar to IQ motif c	*LOC714807*	3.095314	0.1122	+	-	-	-	XM_001103600
hydroxysteroid (17-	*HSD17B1*	4.048115	0.1113	+	-	-	-	NM_001047132 /// XM_001109754
similar to BAI1-ass	*LOC718278*	2.044468	0.1099	+	-	-	-	XM_001110709
Similar to ubiquitin	*LOC706403*	1.930887	0.1028	+	-	-	-	XM_001102149 /// XM_001102237 /// XM_001102331 /// XM_001102423 /// XM_001102518
hypothetical protei	*LOC693608*	1.086124	0.1023	+	-	-	-	XM_001082320
ubiquitin-conjugati	*UBE2C*	3.251114	0.1019	+	-	-	-	XM_001104061
similar to leiomodi	*LOC696239*	1.412844	0.1012	+	-	-	-	XM_001087095 /// XM_001087217
cell division cycle 2	*CDC2*	1.022234	0.0942	+	-	-	-	XM_001095697 /// XM_001095798 /// XM_001095903
collagen, type IV, a	*COL4A4*	2.044468	0.0873	+	-	-	-	XM_001110249
follistatin	*FST*	4.048115	0.0856	+	-	-	-	XM_001095829
similar to family wit	*LOC716656*	0	0.0807	+	-	-	-	XM_001116668 /// XM_001116674 /// XM_001116680 /// XM_001116688
similar to Histone H	*LOC694975*	0	0.0777	+	-	-	-	XM_001083516
adenylate kinase 5	*AK5*	1.362979	0.0696	+	-	-	-	XM_001102743 /// XM_001102925 /// XM_001103009 /// XM_001103089 /// XM_001103172
similar to Muelleria	*LOC717539*	2.273754	0.0678	+	-	-	-	XR_013651
maelstrom homolog	*MAEL*	1.362979	0.0668	+	-	-	-	XM_001087413

similar to beta-gala	*LOC713552*	4.114412	0.0649	+	-	-	-	XM_001109602
chromosome 1 ope	*C1H1orf59*	0	0.0593	+	-	-	-	XM_001087383
Similar to Alpha-1 (	*LOC696813*	2.92445	0.0493	+	-	-	-	XR_010215
similar to LIM home	*LOC709153*	0	0.0423	+	-	-	-	XM_001097664
similar to Nucleoph	*LOC695010 /// L*	1.022234	0.5538	+	-	-	-	XR_010011 /// XR_010477 /// XR_011764 /// XR_014470
eukaryotic translati	*EIF3F*	4.756981	0.6012	+	-	-	-	XM_001105764 /// XM_001105823 /// XM_001105893
Similar to SHC tran	*LOC711071*	2.92445	0.5818	+	-	-	-	XR_012348
similar to chromodo	*LOC706471*	1.412844	0.572	+	-	-	-	XR_011581
tubulin, beta 2A	*TUBB2A*	3.181323	0.5467	+	-	-	-	NM_001032813
Similar to ribosoma	*LOC697181*	3.181323	0.5801	+	-	-	-	XR_010589
activin A receptor, t	*ACVR2B*	3.482703	0.6495	+	-	-	-	XM_001084713
similar to protein ki	*LOC708029*	4.257852	0.6274	+	-	-	-	XM_001100513 /// XM_001100610
similar to vasculin	*GPBP1L1*	3.495013	0.6637	+	-	-	-	XM_001101335 /// XM_001101605 /// XM_001101703
THO complex 4	*THOC4*	3.495013	0.5867	+	-	-	-	XM_001112225
hypothetical protein	*LOC706284*	2.92445	0.6533	+	-	-	-	XM_001095006
proline rich 3	*PRR3*	4.646275	0.5141	+	-	-	-	XM_001100535
hypothetical protein	*LOC718166*	3.095314	0.5316	+	-	-	-	XM_001110487
hypothetical protein	*LOC722270*	4.756981	0.6694	+	-	-	-	XM_001118437
Similar to Small VC	*LOC700825*	1.775852	0.5697	+	-	-	-	XM_001091376
hypothetical protein	*LOC701261*	2.92445	0.5507	+	-	-	-	XM_001089583
Leukocyte immuno	*LILRBB*	4.048115	0.5259	+	-	-	-	---
interferon regulator	*IRF4*	2.92445	0.6344	+	-	-	-	XM_001094391
hematological and	*HN1L*	3.649076	0.5729	+	-	-	-	XM_001089865 /// XM_001089979
similar to nucleolar	*LOC718202*	3.495013	0.6638	+	-	-	-	XM_001110561
similar to Histone d	*LOC694662*	4.048115	0.6053	+	-	-	-	XR_009889
DEAH (Asp-Glu-Ala	*DHX9*	1.775852	0.6093	+	-	-	-	XM_001114384 /// XM_001114405
GRB2-associated b	*GAB2*	2.46188	0.5953	+	-	-	-	XM_001093686 /// XM_001093917
similar to hyalurona	*LOC710213*	1.362979	0.5767	+	-	-	-	XM_001106183 /// XM_001106244
Poliovirus receptor-	*PVRL2*	3.251114	0.6685	+	-	-	-	NM_001043593 /// XM_001109459 /// XM_001109508
HIP14-related prote	*HIP14L*	3.396112	0.5723	+	-	-	-	XM_001096071 /// XM_001096184
hypothetical protein	*LOC711376*	1.412844	0.6084	+	-	-	-	XR_012406
similar to DEAD (As	*LOC711026*	2.775191	0.6304	+	-	-	-	XM_001109331
similar to RCC1-like	*LOC712305*	3.907825	0.5067	+	-	-	-	XR_012607

similar to nuclear fr	*LOC701445*	3.495013	0.6445	+	-	-	-	XR_011485
similar to WD repea	*LOC709710*	3.495013	0.5629	+	-	-	-	XR_012080
similar to Fanconi a	*LOC704048*	3.495013	0.6274	+	-	-	-	XM_001092387
nuclear cap binding	*NCBP1*	3.649076	0.5035	+	-	-	-	XR_013860
similar to zinc finge	*LOC723011*	3.251114	0.5273	+	-	-	-	XM_001119083
Similar to linker for	*LOC704656*	4.222205	0.6499	+	-	-	-	XM_001102058
similar to RAD51 h	*LOC714828*	2.92445	0.5096	+	-	-	-	XM_001104781
similar to colony sti	*LOC711512*	4.048115	0.5993	+	-	-	-	XM_001107711 /// XM_001107833
Similar to carbamo	*LOC699460*	3.482703	0.5848	+	-	-	-	XR_011163
chaperonin contain	*CCT2*	2.46188	0.5606	+	-	-	-	XM_001108460
WD repeat domain	*WDR21A*	4.756981	0.5439	+	-	-	-	XM_001085494 /// XM_001085621 /// XM_001085744 /// XM_001085856
Similar to FYVE, RI	*LOC698436*	3.495013	0.6027	+	-	-	-	XM_001084734 /// XM_001084857 /// XM_001084967 /// XM_001085322 /// XM_001085434 /// XR_010950
similar to S100P bi	*LOC709096*	2.46188	0.6068	+	-	-	-	XM_001105254
mutS homolog 2	*MSH2*	3.181323	0.5245	+	-	-	-	XR_013778
similar to Rho GTP	*LOC710956*	2.92445	0.6073	+	-	-	-	XM_001108281 /// XM_001108338 /// XM_001108455 /// XM_001108514 /// XM_001108566 /// XM_001108619
LSM3 homolog, U6	*LSM3*	3.649076	0.6556	+	-	-	-	XM_001092186
similar to Protein C	*LOC710992*	2.92445	0.6453	+	-	-	-	XR_012335
hypothetical protei	*LOC700505*	2.417806	0.5866	+	-	-	-	XM_001098673 /// XM_001110539 /// XM_001110579 /// XR_009679 /// XR_011685
Theta defensin 1a	*LOC574122*	3.482703	0.6009	+	-	-	-	NM_001032817
hypothetical protei	*LOC701856*	4.048115	0.541	+	-	-	-	XR_011762
similar to chaperon	*LOC706098*	4.048115	0.641	+	-	-	-	XM_001089869 /// XM_001090105 /// XM_001090218 /// XR_011773
RNA binding motif	*RBM4*	3.010359	0.5876	+	-	-	-	XM_001108929 /// XM_001108984 /// XM_001109150 /// XM_001109362 /// XM_001109416
thimet oligopeptida	*THOP1*	4.495104	0.6188	+	-	-	-	XM_001117760
Similar to Cytochro	*LOC722081*	3.251114	0.6575	+	-	-	-	XR_014702
protein arginine me	*PRMT7*	2.044468	0.5358	+	-	-	-	XM_001099129 /// XM_001099228
Hermansky-Pudlak	*HPS4*	2.92445	0.5487	+	-	-	-	XM_001099509 /// XM_001099910
zinc finger protein 2	*ZNF208*	3.495013	0.5792	+	-	-	-	XM_001113534
similar to ret finger	*LOC707239*	3.069357	0.5656	+	-	-	-	XM_001104514

suppressor of defe	*SUDS3*	4.222205	0.6228	+	-	-	-	XM_001084823
similar to DNA poly	*LOC713853*	3.482703	0.5611	+	-	-	-	XM_001103318
heterogeneous nuc	*HNRNPC*	3.251114	0.5709	+	-	-	-	XM_001095254 /// XM_001095481 ///<br>XM_001095687 /// XM_001095788 ///<br>XM_001096357 /// XM_001096469 ///<br>XM_001096584 /// XM_001096688 ///<br>XM_001096801
similar to CG5323-	*LOC702718*	4.048115	0.5658	+	-	-	-	XM_001099918
similar to heteroge	*LOC709112*	2.69426	0.5641	+	-	-	-	XM_001104959 /// XM_001105129 ///<br>XM_001105200 /// XM_001105271 ///<br>XM_001105335 /// XM_001105416 ///<br>XM_001105487 /// XM_001105555 ///<br>XM_001105628 /// XM_001105699 ///<br>XM_001105769 /// XM_001105828 ///<br>XM_001105897 /// XM_001105973 ///<br>XM_001106046 /// XM_001106112 ///
Neural cell adhesio	*NCAM1*	2.780477	0.5616	+	-	-	-	XM_001082236 /// XM_001082361 ///<br>XM_001083241 /// XM_001083366 ///<br>XM_001083486 /// XM_001083597 ///<br>XM_001083697 /// XM_001083809 ///<br>XM_001083924
hypothetical protei	*LOC703785*	2.92445	0.634	+	-	-	-	XM_001090217
similar to death eff	*LOC719875*	2.044468	0.6486	+	-	-	-	XM_001117730 /// XM_001117731 ///<br>XM_001117735 /// XM_001117737 ///<br>XM_001117740
splicing factor, argi	*SFRS6*	2.46188	0.589	+	-	-	-	XM_001084701
trophinin	*TRO*	2.044468	0.6275	+	-	-	-	XM_001092862
Similar to Aprataxi	*LOC702671*	3.181323	0.6597	+	-	-	-	XM_001090958
similar to mucin an	*LOC717579*	4.7194	0.5069	+	-	-	-	XM_001115415 /// XM_001115429
similar to Ubiquitin-	*LOC722434*	2.92445	0.6084	+	-	-	-	XM_001118584
similar to RB-assoc	*LOC716026*	3.495013	0.5838	+	-	-	-	XM_001106540
similar to zinc finge	*LOC719069*	2.273754	0.5965	+	-	-	-	XM_001112671
similar to heteroge	*LOC711941*	2.775191	0.5724	+	-	-	-	XR_012531
similar to membran	*LOC717473*	3.181323	0.5917	+	-	-	-	XM_001115730
CD94	*LOC574145*	4.756981	0.6298	+	-	-	-	NM_001032828
similar to Nuclear t	*LOC719697*	1.362979	0.5111	+	-	-	-	XM_001117254 /// XM_001117257 ///<br>XM_001117262 /// XM_001117267
similar to tubulin, b	*LOC709979*	2.92445	0.6115	+	-	-	-	XR_011014 /// XR_012341
similar to Fanconi a	*LOC718889*	4.222205	0.6368	+	-	-	-	XM_001116683
similar to LAS1-like	*LOC709053*	3.095314	0.6445	+	-	-	-	XR_012095

eukaryotic translati	*EIF3L*	2.92445	0.6145	+	-	-	-	XM_001089709 /// XM_001090065
similar to prickle-lik	*LOC701254*	3.251114	0.6347	+	-	-	-	XM_001089576
CSE1 chromosome	*CSE1L*	2.92445	0.5748	+	-	-	-	XM_001100361 /// XM_001100454 /// XM_001100553 /// XM_001100735
PHD finger protein	*PHF8*	3.482703	0.5349	+	-	-	-	XR_011174
Inositol(myo)-1(or 4	*IMPA2*	3.495013	0.6325	+	-	-	-	XM_001096082 /// XM_001096318
DAZ associated pro	*DAZAP1*	2.92445	0.6031	+	-	-	-	XM_001094635 /// XM_001094757
chromosome 10 op	*C9H10orf119*	2.92445	0.6038	+	-	-	-	XM_001099969
t-complex 1	*TCP1*	2.775191	0.563	+	-	-	-	XM_001097116 /// XM_001097333 /// XM_001097437 /// XM_001097535 /// XM_001097631 /// XM_001098115
heterogeneous nuc	*HNRPH1*	3.495013	0.5658	+	-	-	-	XM_001098332 /// XM_001099039 /// XM_001099249 /// XM_001099346 /// XM_001099448 /// XM_001099754 /// XM_001099955 /// XM_001100049 /// XM_001100137
acidic ribosomal ph	*LOC702073 /// L*	4.048115	0.6642	+	-	-	-	XM_001087189 /// XR_011652 /// XR_012192 /// XR_014511 /// XR_014620
coiled-coil domain c	*CCDC50*	4.222205	0.6628	+	-	-	-	XM_001094521 /// XM_001094644
NIMA (never in mito	*NEK3*	4.257852	0.539	+	-	-	-	XM_001106886 /// XM_001106955 /// XM_001107019
Hypothetical protei	*LOC696050*	3.495013	0.6626	+	-	-	-	XM_001085398 /// XM_001085513 /// XM_001085757
structure specific re	*SSRP1*	4.222205	0.5872	+	-	-	-	XM_001103334
polypyrimidine tract	*PTBP1*	2.273754	0.5655	+	-	-	-	XM_001092088 /// XM_001092214 /// XM_001092330
Similar to patatin-lik	*LOC700824*	2.775191	0.5761	+	-	-	-	XR_010674
RPGRIP1-like	*RPGRIP1L*	3.251114	0.6052	+	-	-	-	XM_001086391 /// XM_001086628 /// XM_001086752
nucleoporin 155kDa	*NUP155*	3.010359	0.604	+	-	-	-	XM_001094346
poly(A) binding prot	*PABPC4*	2.92445	0.5674	+	-	-	-	XM_001113342
Serpin peptidase in	*SERPINA5*	3.010359	0.5798	+	-	-	-	NM_001044733 /// XM_001099865 /// XM_001099962 /// XM_001100054 /// XM_001100142 /// XM_001100235 /// XM_001100324 /// XM_001100411 /// XM_001100511
Similar to G protein	*LOC694500*	2.044468	0.5616	+	-	-	-	XM_001083657 /// XM_001083760
prolyl 4-hydroxylase	*P4HA1*	4.7194	0.6158	+	-	-	-	XM_001103799 /// XM_001103971 /// XM_001104057 /// XM_001104142
similar to Histone d	*LOC694662*	4.222205	0.6082	+	-	-	-	XR_009889
prenyl (decaprenyl)	*PDSS1*	2.92445	0.5873	+	-	-	-	XM_001102166 /// XM_001102259

similar to microfilan	*LOC718651*	3.095314	0.6551	+	-	-	-	XR_013970
Similar to phosphat	*LOC706649*	3.649076	0.6204	+	-	-	-	XM_001104375
similar to splicing fa	*LOC713776*	3.495013	0.6461	+	-	-	-	XM_001103237
similar to bromodoi	*LOC713107*	4.048115	0.601	+	-	-	-	XM_001098494 /// XM_001098590 /// XM_001098692 /// XM_001098795 /// XM_001098901 /// XM_001098998 /// XM_001099107
similar to basic leu	*LOC714627*	3.495013	0.5709	+	-	-	-	XM_001104484
Hypothetical protei	*LOC702801*	3.251114	0.6248	+	-	-	-	XR_011070
similar to zinc finge	*LOC708866*	1.775852	0.5835	+	-	-	-	XM_001097368
NEDD8 ultimate bu	*NYREN18*	1.775852	0.6223	+	-	-	-	XM_001104453 /// XM_001104529
very low density lip	*VLDLR*	4.048115	0.5263	+	-	-	-	NM_001032907
3-hydroxy-3-methyl	*HMGCR*	3.010359	0.5273	+	-	-	-	XM_001103958 /// XM_001104378 /// XM_001104462 /// XM_001104536 /// XM_001104607
similar to heat shoc	*LOC709585*	4.048115	0.5131	+	-	-	-	XM_001106646 /// XM_001106903 /// XM_001106968
similar to protein pl	*LOC706955*	3.649076	0.5403	+	-	-	-	XM_001101248 /// XM_001101345 /// XM_001101437 /// XM_001101528 /// XM_001101616
similar to coiled-coi	*LOC718348*	1.022234	0.6324	+	-	-	-	XM_001116564 /// XM_001116599 /// XM_001116623
similar to two AAA	*LOC704478*	4.646275	0.5331	+	-	-	-	XR_012440
zinc finger protein 1	*ZNF184*	2.044468	0.6657	+	-	-	-	XM_001094409 /// XM_001094532 /// XM_001094654 /// XM_001094778
polymerase (DNA c	*POLD1*	4.7194	0.5492	+	-	-	-	XM_001116048 /// XM_001116065
TSR1, 20S rRNA a	*TSR1*	4.646275	0.5378	+	-	-	-	XM_001086730
similar to Mdm4, tra	*LOC718062*	3.482703	0.557	+	-	-	-	XM_001117173
similar to nucleoph	*LOC697676 /// L*	4.646275	0.6103	+	-	-	-	XM_001095288 /// XM_001099639 /// XM_001099733 /// XR_010527
Similar to complem	*LOC711817*	3.495013	0.5283	+	-	-	-	XM_001100754 /// XM_001100835 /// XM_001100940
similar to zinc finge	*LOC709906*	4.646275	0.6614	+	-	-	-	XM_001098471
serine hydroxymeth	*SHMT1*	0	0.5074	+	-	-	-	XM_001096193 /// XM_001096314 /// XM_001096436 /// XM_001096547 /// XM_001096653 /// XM_001096755 /// XM_001096871 /// XM_001096982
similar to membran	*LOC710049*	2.775191	0.6331	+	-	-	-	XM_001105583 /// XM_001105651 /// XM_001105724
hypothetical protei	*LOC709214 /// L*	3.095314	0.6375	+	-	-	-	XM_001103237 /// XM_001106049
similar to fusion (in	*LOC713433*	3.095314	0.6393	+	-	-	-	XM_001112596 /// XM_001112628 /// XM_001113691
SET domain contai	*SETD4*	2.044468	0.5815	+	-	-	-	XM_001085337 /// XM_001085470 /// XM_001085591 /// XM_001085715 /// XM_001085828

MKI67 (FHA domai	*MKI67IP*	2.273754	0.6344	+	-	-	-	XM_001085690
zinc finger protein 8	*ZNF8*	4.222205	0.6321	+	-	-	-	XM_001118943
testis expressed 10	*TEX10*	3.482703	0.5365	+	-	-	-	XM_001112220 /// XM_001112298 /// XM_001112329
dyskeratosis conge	*DKC1*	3.649076	0.5716	+	-	-	-	XM_001090867
glucuronic acid epi	*GLCE*	3.251114	0.6576	+	-	-	-	XM_001085491 /// XM_001085618
RNA binding motif	*RBM34*	0	0.5113	+	-	-	-	XM_001102020 /// XM_001102111
similar to CG5514-	*LOC712248*	3.181323	0.5226	+	-	-	-	XM_001101185
splicing factor 3a, s	*SF3A3*	3.482703	0.6326	+	-	-	-	XR_014105
adenine phosphoril	*APRT*	2.92445	0.591	+	-	-	-	XM_001089636 /// XM_001089867
poly (ADP-ribose) p	*PARP1*	4.114412	0.584	+	-	-	-	XM_001090628 /// XM_001090751 /// XM_001090871 /// XM_001090984
similar to interleuki	*LOC713427*	2.46188	0.5963	+	-	-	-	XM_001102411
similar to WD repea	*LOC722123*	2.92445	0.6459	+	-	-	-	XM_001118316
similar to DNA poly	*LOC720356*	3.649076	0.5583	+	-	-	-	XM_001115742
zinc finger protein 2	*ZNF22*	3.495013	0.5598	+	-	-	-	XM_001101436
centrosomal protei	*CEP57*	2.775191	0.6085	+	-	-	-	XM_001093124 /// XM_001093231 /// XM_001093445 /// XM_001093565
hypothetical protei	*LOC696606*	2.775191	0.5064	+	-	-	-	XM_001086735 /// XM_001087206 /// XM_001087318 /// XM_001087439
fibrillarin	*FBL*	4.114412	0.5399	+	-	-	-	XM_001088664
cleavage and polya	*CPSF1*	2.775191	0.6463	+	-	-	-	XR_011108
ankyrin repeat dom	*ANKRD10*	1.775852	0.568	+	-	-	-	XM_001084642 /// XM_001084758 /// XM_001084875 /// XM_001084990 /// XM_001085115
zinc finger and SCA	*ZSCAN16*	1.362979	0.5087	+	-	-	-	XM_001098323
chromosome 12 op	*C11H12orf35*	1.579816	0.6029	+	-	-	-	XM_001084139 /// XM_001084251 /// XM_001084371
similar to chloride i	*LOC721634*	3.010359	0.5987	+	-	-	-	XM_001117823
Similar to Protein C	*LOC715966*	3.482703	0.6694	+	-	-	-	XM_001112658
hypothetical protei	*LOC709214*	2.775191	0.6264	+	-	-	-	XM_001106049
similar to large sub	*LOC693645 /// L*	4.222205	0.6285	+	-	-	-	XM_001082219 /// XM_001089531 /// XM_001089653 /// XM_001089766 /// XM_001094570 /// XM_001102282 /// XM_001102532 /// XM_001104426 /// XM_001113675
similar to DNA-repa	*LOC722296*	3.396112	0.5666	+	-	-	-	XM_001118460

similar to NS1-assc	*LOC696756*	4.646275	0.5957	+	-	-	-	XM_001088395 /// XM_001088618 ///<br>XM_001088724 /// XM_001088849 ///<br>XM_001088952 /// XM_001089067 ///<br>XM_001089188 /// XM_001089294 ///<br>XM_001089415 /// XM_001089521 ///<br>XM_001089645 /// XM_001089759
similar to adaptor-r	*LOC710171*	4.114412	0.6074	+	-	-	-	XR_012175
Similar to Double-s	*LOC722248*	3.095314	0.6489	+	-	-	-	XR_014726
hypermethylated in	*HIC2*	2.92445	0.5433	+	-	-	-	XM_001086914
similar to Metastasi	*LOC710095*	2.306811	0.594	+	-	-	-	XR_012162
similar to mitochon	*LOC710571*	1.412844	0.5802	+	-	-	-	XM_001095653 /// XM_001095754 ///<br>XM_001095868 /// XM_001095976 ///<br>XM_001096080
Protocadherin alph	*PCDHA5*	3.649076	0.5746	+	-	-	-	XM_001087166
zinc finger protein 1	*ZNF146*	4.257852	0.6434	+	-	-	-	XM_001103038
similar to additional	*LOC711799*	3.495013	0.5585	+	-	-	-	XM_001107681 /// XM_001107740 ///<br>XM_001107802 /// XM_001107867 ///<br>XM_001107928 /// XM_001107985 ///<br>XM_001108038
lysine (K)-specific c	*KDM1*	3.181323	0.543	+	-	-	-	XM_001111525
ubiquitin specific pe	*USP30*	3.095314	0.5681	+	-	-	-	XM_001104010 /// XM_001104096
pericentrin	*PCNT*	3.251114	0.6642	+	-	-	-	XM_001117998
THO complex 3	*THOC3*	2.92445	0.5429	+	-	-	-	XM_001083281 /// XM_001083412
similar to MutL prot	*LOC712565*	3.010359	0.5433	+	-	-	-	XM_001102056
programmed cell d	*PDCD2L*	2.775191	0.5042	+	-	-	-	XM_001090900 /// XM_001091018
nuclear prelamin A	*NARF*	2.775191	0.6619	+	-	-	-	XM_001113437 /// XM_001113463 ///<br>XM_001113522 /// XM_001113550
Similar to regulator	*LOC703125*	3.495013	0.5809	+	-	-	-	XM_001097555 /// XM_001097657
tyrosyl-DNA phospl	*TDP1*	3.095314	0.5039	+	-	-	-	XM_001088865 /// XM_001088974 ///<br>XM_001089084 /// XM_001089202 ///<br>XM_001089310 /// XM_001089545 ///<br>XM_001089662 /// XM_001089775
similar to MAX prot	*MAX*	4.222205	0.6007	+	-	-	-	XM_001103369
similar to zinc finge	*LOC712885*	2.775191	0.57	+	-	-	-	XM_001102012
Similar to trace-am	*LOC714255*	1.775852	0.5463	+	-	-	-	XR_012952
LSM5 homolog, U6	*LSM5*	4.222205	0.6237	+	-	-	-	XM_001082513
Similar to Ect3 CG:	*LOC700650*	3.095314	0.5969	+	-	-	-	XM_001088996
Similar to NADH-ub	*LOC721259*	2.775191	0.4476	+	4.95028	0.51	+	XM_001117356
similar to ASF1 ant	*LOC714761*	1.775852	0.4103	+	4.95028	0.506	+	XM_001110511

thymopoietin	*TMPO*	1.579816	0.369	+	4.95028	0.4575	+	XM_001082220 /// XM_001082600
Leukocyte immuno	*LILRAE*	2.775191	0.2056	+	4.95028	0.2761	+	NM_001040674 /// XM_001085526
fibroblast growth fa	*FGF14*	2.131262	0.1664	+	4.95028	0.1707	+	XM_001093457 /// XM_001093573
cyclin B1	*CCNB1*	3.095314	0.1341	+	4.95028	0.1945	+	XM_001090925 /// XM_001091170 /// XM_001091421 /// XM_001091533
similar to cyclin B2	*LOC702184*	2.775191	0.0917	+	4.95028	0.1184	+	XR_012150
pituitary tumor-tran	*PTTG1*	1.086124	0.1803	+	4.7013	0.2017	+	XM_001085022 /// XM_001085145 /// XM_001085259
similar to Ribose-5-	*RPIA*	1.775852	0.5446	+	4.7013	0.6648	+	XM_001094618
heterogeneous nuc	*HNRNPD*	4.7194	0.66	+	4.48191	0.5343	+	XM_001088727 /// XM_001089070 /// XM_001089190 /// XM_001089297
WAS/WASL intera	*WIPF3*	2.044468	0.1777	+	4.48191	0.1642	+	XM_001087108 /// XM_001087227
Leukocyte immuno	*LILRBC*	1.579816	0.1167	+	4.48191	0.1318	+	NM_001040676 /// XM_001085404
insulin-like growth f	*IGF2BP3*	0	0.0429	+	4.48191	0.1042	+	XM_001098017 /// XM_001098318 /// XM_001098422
trimethylguanosine	*TGS1*	3.482703	0.608	+	4.48191	0.6351	+	XM_001084205 /// XM_001084320
tescalcin	*TESC*	1.022234	0.1702	+	4.33453	0.332	+	XM_001082221
similar to CTD (car	*LOC711917*	3.649076	0.6496	+	4.33453	0.6626	+	XM_001110266 /// XM_001110308 /// XM_001110346 /// XM_001110393 /// XM_001110440 /// XM_001110479
hypothetical protein	*LOC718881*	2.044468	0.4831	+	3.7902	0.5717	+	XM_001112202
similar to zinc finge	*LOC712142*	3.095314	0.479	+	3.7902	0.5968	+	XM_001099725
similar to lactate de	*LOC693907*	1.086124	0.0859	+	3.7902	0.179	+	XM_001083122 /// XM_001083239 /// XM_001083363
Ly1 antibody reacti	*LYAR*	2.306811	0.392	+	3.39538	0.4287	+	XM_001096675 /// XM_001096786 /// XM_001096901
suppressor of varie	*SUV39H2*	2.273754	0.331	+	3.01812	0.4596	+	XR_010683
similar to transmen	*LOC714325*	1.412844	0.2585	+	3.01812	0.3007	+	XM_001111550 /// XM_001111593 /// XM_001111631
similar to Histone H	*LOC698238*	4.114412	0.2126	+	3.01812	0.1916	+	XM_001087823
cadherin 15, M-cad	*CDH15*	1.775852	0.1916	+	3.01812	0.1755	+	XR_012266
similar to chromos	*LOC715235*	2.775191	0.1284	+	3.01812	0.1479	+	XM_001102882
Similar to DEAD (A	*LOC708293*	3.396112	0.0964	+	3.01812	0.116	+	XR_013023
Similar to high-mob	*LOC701187*	0	0.3963	+	2.58696	0.4222	+	XR_011422
DNA (cytosine-5-)-m	*DNMT3A*	1.362979	0.3167	+	2.58696	0.3844	+	XM_001083234 /// XM_001083358 /// XM_001083479
Hypothetical protei	*LOC697443*	2.92445	0.2848	+	2.58696	0.3541	+	XR_010822
Rho guanine nucle	*ARHGEF7*	2.273754	0.2318	+	2.58696	0.236	+	XR_010342
similar to RAD51 h	*LOC708579*	2.273754	0.1935	+	2.58696	0.2736	+	XM_001097058
similar to cell divisi	*LOC711581*	1.086124	0.1649	+	2.58696	0.2561	+	XR_013479

similar to ubiquitin-l	*LOC695531*	1.362979	0.0687	+	2.58696	0.0856	+	XM_001082762 /// XM_001082893 /// XM_001083019 /// XM_001083134
NLR family, pyrin d	*NLRP7*	0	0.0337	+	2.58696	0.0553	+	NM_001114353 /// NM_001114354 /// XR_010295
ephrin-B3	*EFNB3*	0	0.3102	+	2.18275	0.4317	+	XM_001110580
similar to SH3 dom	*LOC707199*	0	0.3053	+	2.18275	0.4462	+	XR_011694
Similar to pregnanc	*LOC714549*	1.022234	0.1393	+	2.18275	0.1739	+	XR_014018
similar to denticlele	*LOC711236*	4.646275	0.3276	+	1.66305	0.2559	+	XM_001108416
similar to G-protein	*LOC696458*	1.930887	0.3158	+	1.66305	0.4577	+	XR_010411
ureidopropionase, l	*UPB1*	2.131262	0.178	+	1.66305	0.0433	+	XM_001095184 /// XM_001095304
Opa interacting pro	*OIP5*	1.986055	0.1147	+	1.66305	0.1545	+	XM_001100229
R-spondin 2 homol	*RSPO2*	1.086124	0.1147	+	1.66305	0.1476	+	XM_001089208 /// XM_001089315 /// XM_001089438
GINS complex sub	*GINS2*	0	0.0969	+	1.66305	0.1559	+	XM_001082775
similar to Protein C	*LOC719026*	1.775852	0.5748	+	1.66305	0.6537	+	XM_001112551
BRCA1 associated	*BARD1*	2.044468	0.2966	+	0.84885	0.3524	+	XM_001084147 /// XM_001084377 /// XM_001084499 /// XM_001084623 /// XM_001084740
similar to Heteroge	*LOC707925 /// L*	3.482703	0.5822	+	0.84885	0.4079	+	NM_001042783 /// XM_001109413 /// XR_010780 /// XR_010848 /// XR_012339 /// XR_012371 /// XR_012982 /// XR_013591 /// XR_014497
nuclear autoantiger	*NASP*	2.131262	0.487	+	0	0.4166	+	XM_001103253 /// XM_001103320
Thrombomodulin	*THBD*	1.022234	0.45	+	0	0.4251	+	XM_001095416
similar to low densi	*LOC717748*	1.775852	0.4211	+	0	0.457	+	XM_001109580
similar to Wolf-Hirs	*LOC712618*	2.306811	0.2922	+	0	0.3421	+	XR_012450
aurora kinase A	*AURKA*	2.044468	0.2893	+	0	0.1933	+	XR_011039
minichromosome n	*MCM3*	1.086124	0.212	+	0	0.2856	+	XM_001106702 /// XM_001106899 /// XM_001106966
similar to Ig epsilon	*IGHE*	2.92445	0.2107	+	0	0.2288	+	XM_001116734
WAS protein family	*WASF1*	0.526605	0.2101	+	0	0.2458	+	XM_001087511
hypothetical proteir	*LOC711569*	1.930887	0.2027	+	0	0.3446	+	XM_001104895
similar to MLF1 inte	*LOC696925*	1.022234	0.1592	+	0	0.2188	+	XR_010233
hypothetical proteir	*LOC717634*	0	0.1368	+	0	0.1596	+	XM_001109346
transforming, acidic	*TACC3*	0	0.1238	+	0	0.1866	+	XM_001101192 /// XM_001101279
similar to actin like	*LOC701280*	0	0.1044	+	0	0.1501	+	XM_001090405
Heat shock transcr	*HSF4*	1.775852	0.0984	+	0	0.1083	+	XR_010449
T-cell leukemia/lym	*TCL1B*	0	0.0947	+	0	0.1426	+	XM_001101160 /// XM_001101242

Transmembrane pr	*TMPRSS3*	1.086124	0.0869	+	0	0.1209	+	XM_001105565 /// XM_001105638 /// XM_001105841 /// XM_001105909 /// XM_001106059 /// XM_001106126
NLR family, pyrin d	*NLRP11*	0	0.064	+	0	0.0672	+	NM_001114109 /// XM_001092392
Similar to c11.1 CG	*LOC713971*	0	0.0624	+	0	0.0811	+	XR_012905
hypothetical protein	*LOC706226*	0	0.0445	+	0	0.0627	+	XM_001094581
similar to Tubulin b	*LOC718907*	0	0.0361	+	0	0.0547	+	XR_014035
zygote arrest 1	*ZAR1*	0	0.0359	+	0	0.054	+	XM_001103446
zona pellucida glyc	*ZP3*	0	0.0347	+	0	0.0804	+	XM_001114760
NLR family, pyrin d	*NLRP4*	0	0.0274	+	0	0.0362	+	XM_001090206 /// XM_001090323 /// XM_001090438
similar to tubulin, b	*LOC703074*	0	0.0265	+	0	0.0294	+	XR_010378
similar to ELAV-like	*LOC708195*	0	0.0259	+	0	0.0439	+	XM_001106242 /// XM_001106303 /// XM_001106373 /// XM_001106435 /// XM_001106502
similar to stella	*LOC712263*	0	0.024	+	0	0.0378	+	XM_001111317
similar to Factor in	*LOC710606*	0	0.0224	+	0	0.054	+	XM_001099273
BCL2-like 10 (apop	*BCL2L10*	0	0.0144	+	0	0.0179	+	XM_001085850
NLR family, pyrin d	*NLRP5*	0	0.0068	+	0	0.0145	+	NM_001127631 /// XM_001118932 /// XR_011260
similar to tubulin, b	*LOC703074 /// L*	0	0.0047	+	0	0.0057	+	XM_001104035 /// XM_001104203 /// XR_010378 /// XR_013160
dickkopf homolog 3	*DKK3*	-	-	-	4.33453	0.4889	+	XM_001097713 /// XM_001097811 /// XM_001097909
thrombospondin 1	*THBS1*	-	-	-	3.7902	0.3518	+	XM_001093770
similar to nel-like 1	*LOC701438*	-	-	-	0	0.2704	+	XM_001092428 /// XM_001092540 /// XM_001092655
similar to Collagen	*LOC722009*	-	-	-	0	0.5456	+	XM_001118209
similar to transcript	*LOC696006*	-	-	-	4.95028	0.5354	+	XR_010513
similar to heteroger	*LOC713066*	-	-	-	3.7902	0.543	+	XM_001102264
SWI/SNF related, r	*SMARCE1*	-	-	-	3.01812	0.6624	+	XM_001099001 /// XM_001099419 /// XM_001099528 /// XM_001099623
similar to mortality f	*LOC711357*	-	-	-	3.7902	0.661	+	XM_001108512 /// XM_001108618 /// XM_001108662
heterogeneous nuc	*HNRPDL*	-	-	-	2.18275	0.5597	+	XM_001087953 /// XM_001088081 /// XM_001088191 /// XM_001088302 /// XM_001088399 /// XM_001088510

*Q-value, false discovery rate-adjusted p-value; red, upregulated; green, downregulated; +, significant difference (p<0.05); -, nonsignificant difference. Genes highlighted in yellow were functionally obscure in *M. mulatta* .

Supplemental Table S2. GO terms of genes differentially expressed in old vs. young and middle-aged monkey ovaries

Biological process

GO Term	Number	p-Value	q-Value*	Protein
GO:0006355 regulation of transcription, DNA-dependent	31	1.53E-24	3.36E-23	JARID2;CDC5L;FUBP1;ZNF93;ZNF167;ZNF430;TCF3;TGIF;HMGA1;SOX4;POU3F1;BNC1;WHSC1;REIIBP;MCM7;MCM3;LHX2;LHX8;FIGLA;LOC91661;SFPQ;HNRPD;RUVBL2;GTF2F2;BCLAF1;RBM14;SAFB;NFYA;SSRP1;HDAC2;MAX
GO:0007049 cell cycle	22	1.14E-20	1.79E-19	RAP1A;CDC5L;RAD50;SMC4L1;SPAG5;MAD2L1;STK6;MCM7;CHEK1;KIF23;MCM3;ZWINT;CKS2;BUB1B;CDC20;UBE2C;CDC2;CCNB2;BUB1;POLS;KATNA1;MLH1
GO:0007067 mitosis	14	1.74E-20	2.40E-19	PLK1;SPAG5;MAD2L1;STK6;KIF23;BUB1B;KIF2C;CDC20;UBE2C;CDC2;CCNB2;BUB1;POLS;KATNA1
GO:0051301 cell division	13	2.05E-18	2.50E-17	SMC4L1;SPAG5;MAD2L1;KIF23;CKS2;BUB1B;CDC20;UBE2C;CDC2;CCNB2;BUB1;POLS;KATNA1
GO:0006281 DNA repair	12	1.57E-14	1.44E-13	RFC4;DKFZp564E2182;BRCA1;CHEK1;TYMS;RAD51;SFPQ;RUVBL2;RBM14;POLD1;PARP1;POLG2
GO:0007018 microtubule-based movement	7	5.67E-14	4.45E-13	TUBB;KIF23;TUBB2C;KIF2C;TUBB2A;Q9GLW6_MACMU;TBAK_HUMAN_hsa-homolog
GO:0048015 phosphoinositide-mediated signaling	9	8.68E-14	6.37E-13	RFC4;FEN1;SPAG5;STK6;TYMS;ZWINT;CKS2;BUB1B;UBE2C
GO:0006350 transcription	23	2.46E-12	1.59E-11	ATF5;JARID2;FUBP1;ZNF93;ZNF167;ZNF430;HMGA1;SOX4;BNC1;MCM7;MCM3;SFPQ;HNRPD;RUVBL2;GTF2F2;ZNF354A;BCLAF1;RBM14;SAFB;ILF3;NFYA;ILF2;HDAC2
GO:0007051 spindle organization and biogenesis	6	2.98E-12	1.82E-11	SPAG5;STK6;ZWINT;CKS2;BUB1B;UBE2C
GO:0006260 DNA replication	9	5.13E-11	2.69E-10	NAP1L1;RFC4;FEN1;MCM7;TYMS;MCM3;RBM14;POLS;POLD1

GO:0000398 nuclear mRNA splicing, via spliceosome	8	1.14E-10	5.70E-10	CDC5L;SFPQ;DDX46;LSM3;SFRS6;HNRPM;SRPK1;SFRS1
GO:0051258 protein polymerization	5	5.03E-10	2.40E-09	TUBB;TUBB2C;TUBB2A;Q9GLW6_MACMU;TBAK_HUMAN_hsa-homolog
GO:0008283 cell proliferation	11	5.83E-09	2.56E-08	IGFBP4;SIL;PLK1;MDK;CKS2;NDP;BUB1B;KIF2C;BUB1;SKB1;CSF1R
GO:0046549 retinal cone cell development	2	1.31E-08	4.88E-08	THY1;AAC05640
GO:0048147 negative regulation of fibroblast proliferation	2	1.31E-08	4.88E-08	THY1;AAC05640
GO:0050860 negative regulation of T cell receptor signaling pathway	2	1.31E-08	4.88E-08	THY1;AAC05640
GO:0051281 positive regulation of release of sequestered calcium ion into cytosol	2	1.31E-08	4.88E-08	THY1;AAC05640
GO:0006231 dTMP biosynthesis	3	1.31E-08	4.88E-08	Q8WYK3_HUMAN_hsa-homolog;TYMS;Q8WYK4_HUMAN_hsa-homolog;
GO:0043547 positive regulation of GTPase activity	2	5.22E-08	1.53E-07	THY1;AAC05640
GO:0045576 mast cell activation	2	5.22E-08	1.53E-07	THY1;AAC05640
GO:0050771 negative regulation of axonogenesis	2	5.22E-08	1.53E-07	THY1;AAC05640
GO:0006270 DNA replication initiation	3	7.10E-08	1.95E-07	MCM7;Q9H4N9_HUMAN_hsa-homolog;MCM3
GO:0006468 protein amino acid phosphorylation	10	4.94E-07	1.18E-06	PLK1;STK6;CHEK1;MELK;BUB1B;CDC2;TTK;BUB1;CSF1R;SRPK1

GO:0030336 negative regulation of cell migration	2	7.26E-07	1.60E-06	THY1;AAC05640
GO:0048041 focal adhesion formation	2	7.26E-07	1.60E-06	THY1;AAC05640
GO:0050852 T cell receptor signaling pathway	2	7.26E-07	1.60E-06	THY1;AAC05640
GO:0050731 positive regulation of peptidyl-tyrosine phosphorylation	2	1.55E-06	3.07E-06	THY1;AAC05640
GO:0000074 regulation of progression through cell cycle	8	2.49E-06	4.68E-06	ATF5;PLK1;MDK;CDC20;CDC2;CCNB2;TTK;CCT2
GO:0006457 protein folding	6	2.86E-06	5.16E-06	HSPB8;NPM3;TOMM34;FKBP6;RUVBL2;CCT2
GO:0007131 meiotic recombination	3	4.67E-06	8.03E-06	RAD50;CHEK1;RAD51
GO:0006345 loss of chromatin silencing	2	5.58E-06	9.09E-06	DKFZp564E2182;HMGA1
GO:0031116 positive regulation of microtubule polymerization	1	5.58E-06	9.09E-06	MAPT
GO:0045773 positive regulation of axon extension	1	5.58E-06	9.09E-06	MAPT
GO:0050870 positive regulation of T cell activation	2	1.45E-05	2.24E-05	THY1;AAC05640
GO:0006302 double-strand break repair	3	1.69E-05	2.54E-05	RAD50;FEN1;POLS
GO:0007264 small GTPase mediated signal transduction	5	2.19E-05	3.21E-05	RAP1A;AAZ81000;Q5U0C3_HUMAN_hsa-homolog;RASEF;RRP22
GO:0006469 negative regulation of protein kinase activity	2	3.27E-05	4.47E-05	THY1;AAC05640

GO:0042267 natural killer cell mediated cytotoxicity	2	3.33E-05	4.47E-05	TUBB2C;TUBB2A
GO:0007094 mitotic spindle checkpoint	2	3.33E-05	4.47E-05	TTK;BUB1
GO:0007399 nervous system development	6	6.97E-05	9.12E-05	TPP1;MDK;EFNB3;NDP;THBS1;NELL1
GO:0006636 fatty acid desaturation	2	8.31E-05	1.07E-04	FADS2;Q9H3G3_HUMAN_hsa-homolog
GO:0008284 positive regulation of cell proliferation	4	1.30E-04	1.61E-04	NAP1L1;BNC1;UBE2C;TTK
GO:0043066 negative regulation of apoptosis	3	1.96E-04	2.32E-04	THY1;AAC05640;BARD1
GO:0016575 histone deacetylation	2	1.99E-04	2.32E-04	RBM14;HDAC2
GO:0006813 potassium ion transport	3	2.29E-04	2.60E-04	KCNK3;SK3;KCNN3
GO:0001525 angiogenesis	2	2.36E-04	2.65E-04	THY1;AAC05640
GO:0007076 mitotic chromosome condensation	2	2.48E-04	2.75E-04	SMC4L1;POLS
GO:0006366 transcription from RNA polymerase II promoter	6	2.56E-04	2.82E-04	TAF1A;FUBP1;HSF2BP;TRIP13;NFYA;PARP1
GO:0007155 cell adhesion	6	2.64E-04	2.87E-04	WISP2;BGLAP;MSLN;CDH3;THBS1;NELL1
GO:0006268 DNA unwinding during replication	2	3.02E-04	3.15E-04	HMGA1;RAD51
GO:0007026 negative regulation of microtubule depolymerization	1	3.02E-04	3.15E-04	MAPT
GO:0006376 mRNA splice site selection	2	3.02E-04	3.15E-04	SFRS6;SFRS1

GO:0007267 cell-cell signaling	6	3.04E-04	3.15E-04	WISP2;MDK;EFNB3;STC1;NDP;AMH
GO:0007093 mitotic checkpoint	2	3.62E-04	3.67E-04	MAD2L1;BUB1B
GO:0007605 sensory perception of sound	3	4.46E-04	4.38E-04	NDP;ZNF354A;RPGR
GO:0006334 nucleosome assembly	3	4.90E-04	4.77E-04	NAP1L1;DKFZp564E2182;HIST1H1A
GO:0001558 regulation of cell growth	3	6.64E-04	6.35E-04	WISP2;IGFBP4;RUVBL2
GO:0007218 neuropeptide signaling pathway	3	8.09E-04	7.55E-04	GRP;Q53YA0_HUMAN_hsa-homolog;GPR56
GO:0006310 DNA recombination	3	9.39E-04	8.54E-04	SFPQ;RUVBL2;RBM14
GO:0007283 spermatogenesis	3	1.05E-03	9.35E-04	NASP;HSF2BP;HIST1H1A
GO:0006508 proteolysis	6	1.19E-03	1.04E-03	MMP9;RNF13;TPP1;CASP2;Q1WK24_MACMU;THOP1
GO:0008277 regulation of G-protein coupled receptor protein signaling pathway	2	1.25E-03	1.08E-03	RAMP3;RGS7
GO:0000079 regulation of cyclin-dependent protein kinase activity	2	1.75E-03	1.45E-03	CHEK1;CKS2
GO:0007017 microtubule-based process	2	2.17E-03	1.74E-03	TUBB;Q9GLW6_MACMU
GO:0030325 adrenal gland development	1	2.37E-03	1.74E-03	MDK
GO:0031441 negative regulation of mRNA 3'-end processing	1	2.37E-03	1.74E-03	BARD1

GO:0045732 positive regulation of protein catabolism	1	2.37E-03	1.74E-03	BARD1
GO:0046826 negative regulation of protein export from nucleus	1	2.37E-03	1.74E-03	BARD1
GO:0000072 M phase specific microtubule process	1	2.37E-03	1.74E-03	KPNA2
GO:0000022 mitotic spindle elongation	1	2.37E-03	1.74E-03	KIF23
GO:0051106 positive regulation of DNA ligation	1	2.37E-03	1.74E-03	RAD51
GO:0007506 gonadal mesoderm development	1	2.37E-03	1.74E-03	AMH
GO:0043484 regulation of RNA splicing	1	2.37E-03	1.74E-03	HNRPF
GO:0006168 adenine salvage	1	2.37E-03	1.74E-03	APRT
GO:0042384 cilium biogenesis	1	2.37E-03	1.74E-03	PCNT
GO:0007601 visual perception	3	3.07E-03	2.03E-03	CDH3;NDP;RPGR
GO:0006396 RNA processing	4	3.22E-03	2.11E-03	HNRPD;NUFIP1;HNRPU;HNRPF
GO:0006886 intracellular protein transport	4	3.26E-03	2.12E-03	RAMP3;KPNA2;RPGR;AP1G1
GO:0007126 meiosis	2	3.33E-03	2.16E-03	RAD50;RAD51
GO:0000226 microtubule cytoskeleton organization and biogenesis	2	4.10E-03	2.62E-03	MAPT
GO:0045124 regulation of bone resorption	1	4.73E-03	2.78E-03	BGLAP

GO:0000019 regulation of mitotic recombination	1	4.73E-03	2.78E-03	RAD50
GO:0001570 vasculogenesis	1	0.0047269	2.78E-03	GLMN
GO:0009650 UV protection	1	0.0047269	2.78E-03	FEN1
GO:0008054 cyclin catabolism	1	0.0047269	2.78E-03	UBE2C
GO:0001880 Mullerian duct regression	1	0.0047269	2.78E-03	AMH
GO:0035246 peptidyl-arginine N-methylation	1	0.0047269	2.78E-03	SKB1
GO:0006420 arginyl-tRNA aminoacylation	1	0.0047269	2.78E-03	RARS
GO:0006426 glycyl-tRNA aminoacylation	1	0.0047269	2.78E-03	POLG2
GO:0006633 fatty acid biosynthesis	2	0.0051615	2.84E-03	FADS2;Q9H3G3_HUMAN_hsa-homolog
GO:0016337 cell-cell adhesion	2	0.0062212	3.35E-03	THY1;AAC05640
GO:0006702 androgen biosynthesis	1	0.0070821	3.47E-03	CYP11A1
GO:0050715 positive regulation of cytokine secretion	1	0.0070821	3.47E-03	GLMN
GO:0009157 deoxyribonucleoside monophosphate biosynthesis	1	0.0070821	3.47E-03	TYMS;TYMS
GO:0046882 negative regulation of follicle-stimulating hormone secretion	1	0.0070821	3.47E-03	FST

GO:0042921 glucocorticoid receptor signaling pathway	1	0.0070821	3.47E-03	RBM14
GO:0007062 sister chromatid cohesion	1	0.0070821	3.47E-03	POLS
GO:0006166 purine ribonucleoside salvage	1	0.0070821	3.47E-03	APRT
GO:0000012 single strand break repair	1	0.0070821	3.47E-03	XRCC1
GO:0008544 epidermis development	2	0.0070958	3.47E-03	BNC1;DSP
GO:0048699 generation of neurons	1	0.0073573	3.58E-03	MAPT
GO:0006928 cell motility	3	0.0083862	4.05E-03	TSPAN6;WASF1;THBS1
GO:0007165 signal transduction	12	0.0091822	4.37E-03	WISP2;IGFBP4;CALCRL;GRP;MDK;GPSM2;RTN1;GPR56;GPR39;NDP;CRHBP;CSF1R
GO:0030500 regulation of bone mineralization	1	0.0094316	4.37E-03	BGLAP
GO:0042476 odontogenesis	1	0.0094316	4.37E-03	BGLAP
GO:0042130 negative regulation of T cell proliferation	1	0.0094316	4.37E-03	GLMN
GO:0001894 tissue homeostasis	1	0.0094316	4.37E-03	BARD1
GO:0000085 G2 phase of mitotic cell cycle	1	0.0094316	4.37E-03	KPNA2
GO:0006527 arginine catabolism	1	0.0094316	4.37E-03	ARG2
GO:0006703 estrogen biosynthesis	1	0.0094316	4.37E-03	HSD17B1
GO:0048477 oogenesis	1	0.0094316	4.37E-03	FIGLA

Cellular component

GO Term	Number	p-Value	q-Value*	Protein
GO:0005634 nucleus	76	4.791E-66	5.27E-64	RNF13;ATF5;HNRPA1;JARID2;NAP1L1;SMARCC1;NPM3;FUBP1;ZNF93;;ZNF167;ZNF430;TCF3;RFC4;HNRPA3;TCOF1;DKFZp564E2182;TGIF;HMGA1;FEN1;PLK1;SOX4;SMC4L1;POU3F1;MAD2L1;BARD1;KPNA2;BNC1;MCM7;BRCA1;TRIP13;LMNB1;CHEK1;KIF23;MCM3;RAD51;LHX2;ZWINT;BUB1B;STK6;KIF2C;CDC2;CCNB2;BUB1;HIST1H1A;LHX8;FIGLA;SFPQ;HNRPD;NASP;RUVBL2;GTF2F2;DDX46;NUFIP1;ZNF354A;BCLAF1;RBM14;SAFB;LSM3;ILF3;POLS;SFRS6;NFYA;SSRP1;ILF2;HDAC2;SRPK1;POLD1;SFRS1;PARP1;CEP57;CPSF1;XRCC1;COIL;MLH1;MAX; Q15918_HUMAN_hsa-homolog
GO:0005874 microtubule	10	1.581E-16	1.581E-15	TUBB;SPAG5;KIF23;TUBB2C;KIF2C;TUBB2A;Q9GLW6_MACMU;KATNA1;CEP57; TBAK_HUMAN_hsa-homolog;
GO:0016020 membrane	37	1.828E-11	1.059E-10	CYP11A1;MRAP;KCNK3;ACSL1;FADS2;SK3;KCNN3;THY1;AAC05640;CALCRL;RAP1A;EMP3;IL1R1;TOMM34;TSPAN6;TMPO;RRP22;EFNB3;MSLN;RTN1;GPR56;DKFZp761G1118;PHKA2;BAMBI;CDH3;GPR39;DBH;ZP2;HIG2;M11S1;VLDLR;AAZ80936;Q6S4M1_MACMU;Q96L30_HUMAN_hsa-homolog;SYNGR3; Q9H3G3_HUMAN_hsa-homolog; Q5U0C3_HUMAN_hsa-homolog
GO:0030426 growth cone	3	3.244E-11	1.784E-10	THY1;AAC05640;MAPT
GO:0005829 cytosol	6	1.123E-07	3.012E-07	THY1;AAC05640;MAPT;RUVBL2;CCT2;CCT1
GO:0005819 spindle	4	5.982E-07	1.4E-06	STK6;KIF23;CDC20;TTK
GO:0005887 integral to plasma membrane	12	1.422E-06	2.896E-06	KCNK3;RAMP3;FADS2;THY1;CALCRL;IL1R1;EFNB3;GPR39;CSF1R;HNRPM;M11S1;SYNGR3
GO:0005615 extracellular space	8	1.771E-06	3.417E-06	CHIT1;MMP9;GRP;MDK;NDP;ZP2;AMH;DKK3
GO:0009897 external side of plasma membrane	2	2.832E-06	5.165E-06	THY1;AAC05640
GO:0045121 lipid raft	2	4.67E-06	8.026E-06	THY1;AAC05640

GO:0000776 kinetochore	3	4.67E-06	8.026E-06	MAD2L1;BUB1B;BUB1
GO:0045298 tubulin	1	5.575E-06	9.085E-06	MAPT
GO:0005625 soluble fraction	5	2.982E-05	4.205E-05	WISP2;GRP;DBH;CRHBP;RARS
GO:0030424 axon	1	8.31E-05	0.0001069	MAPT
GO:0000785 chromatin	4	0.0001777	0.0002172	SMARCC1;HMGA1;MCM7;SSRP1
GO:0005737 cytoplasm	15	0.0001972	0.0002323	TUBB;FUBP1;BARD1;KPNA2;BAMBI;TUBB2C;DBH;HSD17B1;TUBB2A;SKB1 ;Q96K27_HUMAN_hsa-homolog;RARS;HDAC2;SRPK1;APRT
GO:0005681 spliceosome complex	3	0.0002223	0.0002548	HNRPA1;CDC5L;HNRPM
GO:0030530 heterogeneous nuclear ribonucleoprotein complex	2	0.0003622	0.0003673	HNRPU;HNRPF
GO:0000781 chromosome, telomeric region	2	0.0004274	0.0004236	RAD50;HNRPD
GO:0005871 kinesin complex	2	0.0008319	0.0007658	KIF23;KIF2C
GO:0005875 microtubule associated complex	2	0.0009738	0.000878	KIF23;MAPT
GO:0016021 integral to membrane	16	0.0012077	0.0010544	MRAP;ACSL1;SK3;KCNN3;EMP3;TOMM34;TMPO;TSPAN6;RTN1;GPR56;DKFZp761G1118;BAMBI;CDH3;ZP2;HIG2; Q9NYP7_HUMAN_hsa-homolog
GO:0005694 chromosome	3	0.0013011	0.0011182	TMPO;SMC4L1;HIST1H1A
GO:0000775 chromosome, pericentric region	2	0.0014867	0.0012629	BIRC5;KIF2C
GO:0005905 coated pit	2	0.0020235	0.0016611	SNAP91;AP1G1
GO:0030496 midbody	1	0.0023663	0.0017411	BIRC5
GO:0042583 chromaffin granule	1	0.0023663	0.0017411	DBH
GO:0051233 spindle midzone	1	0.0023663	0.0017411	BUB1B
GO:0000262 mitochondrial chromosome	1	0.0023663	0.0017411	POLG2

GO:0005847 mRNA cleavage and polyadenylation specificity factor complex	1	0.0023663	0.0017411	CPSF1
GO:0030529 ribonucleoprotein complex	4	0.0029952	0.0019968	HNRPA1;HNRPA3;HNRPD;RBM14
GO:0030870 Mre11 complex	1	0.0047269	0.0027806	RAD50
GO:0031436 BRCA1-BARD1 complex	1	0.0047269	0.0027806	BARD1
GO:0005638 lamin filament	1	0.0047269	0.0027806	LMNB1
GO:0005674 transcription factor TFIIF complex	1	0.0047269	0.0027806	GTF2F2
GO:0005663 DNA replication factor C complex	1	0.0070821	0.003469	RFC4
GO:0000796 condensin complex	1	0.0070821	0.003469	SMC4L1
GO:0005658 alpha DNA polymerase:primase complex	1	0.0070821	0.003469	MCM3
GO:0005816 spindle pole body	1	0.0070821	0.003469	BUB1
GO:0016602 CCAAT-binding factor complex	1	0.0070821	0.003469	NFYA
GO:0005576 extracellular region	7	0.0085289	0.0040969	WISP2;IGFBP4;BGLAP;STC1;DBH;FST;THBS1
GO:0005964 phosphorylase kinase complex	1	0.0094316	0.0043683	PHKA2
GO:0005730 nucleolus	2	0.0108252	0.004821	TCOF1;ILF2

Molecular function

GO Term	Number	p-Value	q-Value*	Protein

GO:0000166 nucleotide binding	56	9.88E-52	5.43E-50	RAP1A;AAZ81000;RAP1A;HNRPA1;DKFZp434J0315;LOC81691;RBMX2;RAD50;RFC4;HNRPA3;PLK1;SMC4L1;RRP22;MCM7;;TRIP13;CHEK1;KIF23;KIF23;MCM3;RAD51;MELK;TUBB2C;NALP14;BUB1B;STK6;KIF2C;CDC2;TTK;BUB1;NALP11;NALP5;TUBB2A;TIA1;SFPQ;HNRPD;RUVBL2;GTF2F2;DDX46;CCT2;RBM14;CSF1R;HNRPU;SAFB;Q9GLW6_MACMU;SFRS6;HNRPM;RARS;TUBB;KATNA1;CCT1;HNRPF;SRPK1;POLD1;SFRS1;hnrpdl; Q5U0C3_HUMAN_hsa-homolog; Q9H4N9_HUMAN_hsa-homolog; TBAK_HUMAN_hsa-homolog
GO:0005524 ATP binding	33	3.14E-28	1.15E-26	RAD50;RFC4;PLK1;SMC4L1;MCM7;TRIP13;CHEK1;KIF23;MCM3;RAD51;MELK;NALP14;BUB1B;STK6;KIF2C;CDC2;TTK;BUB1;NALP11;NALP5;RUVBL2;GTF2F2;DDX46;CCT2;CSF1R;HNRPU;RARS;KATNA1;CCT1;SRPK1;POLG2;MLH1; Q9H4N9_HUMAN_hsa-homolog;
GO:0005515 protein binding	51	5.71E-26	1.57E-24	WISP2;THY1;ARHGEF12;RAP1A;RNF13;IL1R1;CCDC85B;CDC5L;RFC4;P4HA2;GLMN;FEN1;PLK1;SMC4L1;MAD2L1;BARD1;KPNA2;MSLN;WHSC1;REIIBP;STK6;MCM7;BIRC5;MCM3;CDH3;BUB1B;CDC20;CDC2;CCNB2;FST;CRHBP;SFPQ;NUFIP1;BCLAF1;SAFB;LSM3;ILF3;NFYA;THOP1;SSRP1;ILF2;RPGR;HNRPF;SRPK1;KIAA1418;POLD1;SFRS1;CPSF1;XRCC1;MLH1;THBS1
GO:0003677 DNA binding	30	9.79E-23	1.79E-21	JARID2;CDC5L;RAD50;ZNF430;RFC4;HMGA1;TMPO;WHSC1;MCM7;BRCA1;MCM3;HIST1H1A;FIGLA;SMARCC1;SFPQ;HNRPD;GTF2F2;ZNF354A;BCLAF1;HNRPU;SAFB;ILF3;POLS;SSRP1;ILF2;POLD1;PARP1;POLG2;MAX; Q9H4N9_HUMAN_hsa-homolog
GO:0003723 RNA binding	17	1.23E-17	1.36E-16	HNRPA1;CDC5L;SFPQ;RBMX2;HNRPA3;BARD1;TIA1;HNRPD;DDX46;NUFIP1;RBM14;HNRPU;SAFB;LSM3;SFRS6;HNRPF;SFRS1;CPSF1
GO:0008270 zinc ion binding	23	3.19E-14	2.70E-13	MMP9;RNF13;RAD50;ZNF93;ZNF167;ZNF430;Zn15;BARD1;BNC1;WHSC1;REIIBP;BIRC5;BRCA1;LHX2;LHX8;ZAR1;HIP14L;NUFIP1;ZNF354A;THOP1;POLD1;PARP1; Q15918_HUMAN_hsa-homolog

GO:0005525 GTP binding	10	8.30E-13	5.70E-12	RAP1A;AAZ81000;TUBB;RASEF;RRP22;TUBB2C;TUBB2A;Q9GLW6_MACMU;TBAK_HUMAN_hsa-homolog; Q5U0C3_HUMAN_hsa-homolog;
GO:0003700 transcription factor activity	14	1.44E-09	6.61E-09	FUBP1;ZNF93;ZNF167;TCF3;TGIF;HMGA1;SOX4;POU3F1;BNC1;LHX2;LHX8;NFYA;HDAC2;MAX
GO:0004799 thymidylate synthase activity	3	1.31E-08	4.88E-08	Q8WYK3_HUMAN_hsa-homolog;TYMS;Q8WYK4_HUMAN_hsa-homolog
GO:0003924 GTPase activity	6	1.42E-08	4.88E-08	TUBB;RRP22;TUBB2C;TUBB2A;Q9GLW6_MACMU;TBAK_HUMAN_hsa-homolog
GO:0016740 transferase activity	17	1.77E-08	5.90E-08	PLK1;STK6;TYMS;CHEK1;MELK;BUB1B;CDC2;TTK;BUB1;SKB1;CSF1R;POLS;SRPK1;POLD1;POLG2; Q8WYK4_HUMAN_hsa-homolog; Q96K27_HUMAN_hsa-homolog
GO:0051082 unfolded protein binding	7	2.02E-08	6.52E-08	HSPB8;NPM3;TOMM34;TUBB2C;RUVBL2;CCT2;CCT1
GO:0005198 structural molecule activity	9	2.09E-08	6.57E-08	TUBB;NEFL;LMNB1;TUBB2C;TUBB2A;Q9GLW6_MACMU;THBS1;NELL1; TBAK_HUMAN_hsa-homolog
GO:0030161 calpain inhibitor activity	2	5.22E-08	1.53E-07	CAST;Q15786_HUMAN_hsa-homolog
GO:0046872 metal ion binding	21	2.15E-07	5.64E-07	CYP11A1;FADS2;RNF13;RAD50;ZNF93;ZNF167;ZNF430;P4HA2;BARD1;BNC1;BIRC5;LHX2;DBH;ARG2;LHX8;NUFIP1;ZNF354A;THOP1;P4HA1;POLD1;PARP1
GO:0003684 damaged DNA binding	4	3.13E-07	8.00E-07	FEN1;RAD51;RUVBL2;XRCC1
GO:0008017 microtubule binding	3	3.72E-07	9.29E-07	BIRC5;MAPT;KATNA1
GO:0048503 GPI anchor binding	2	4.55E-07	1.11E-06	THY1;AAC05640

GO:0005509 calcium ion binding	10	6.587E-07	1.51E-06	MMP9;MYL9;BGLAP;RASEF;Q96N04_HUMAN_hsa-homolog;CDH3;AAZ80936;Q6S4M1_MACMU;THBS1;NELL1
GO:0008094 DNA-dependent ATPase activity	3	9.141E-07	1.93E-06	MCM7;Q9H4N9_HUMAN_hsa-homolog;MCM3;
GO:0004674 protein serine/threonine kinase activity	8	1.102E-06	2.29E-06	PLK1;STK6;CHEK1;MELK;BUB1B;TTK;BUB1;SRPK1
GO:0031418 L-ascorbic acid binding	3	1.55E-06	3.07E-06	P4HA2;DBH;P4HA1
GO:0005516 calmodulin binding	4	2.508E-06	4.68E-06	SK3;KCNN3;PHKA2;PCNT
GO:0005100 Rho GTPase activator activity	2	3.676E-06	6.52E-06	THY1;AAC05640
GO:0042802 identical protein binding	5	1.272E-05	2.00E-05	GPSM2;RAD51;RUVBL2;PARP1;POLG2
GO:0004656 procollagen-proline 4-dioxygenase activity	2	1.67E-05	2.54E-05	P4HA2;P4HA1
GO:0008083 growth factor activity	5	2.058E-05	3.06E-05	GRP;MDK;DKFZp761G1118;NDP;AMH
GO:0003690 double-stranded DNA binding	3	2.237E-05	3.24E-05	FEN1;RAD51;SAFB
GO:0008168 methyltransferase activity	4	2.77E-05	3.96E-05	TYMS;Q8WYK4_HUMAN_hsa-homolog;SKB1;Q96K27_HUMAN_hsa-homolog
GO:0003697 single-stranded DNA binding	3	3.266E-05	4.47E-05	FUBP1;RAD51;POLG2
GO:0005521 lamin binding	1	3.335E-05	4.47E-05	TMPO
GO:0004527 exonuclease activity	3	0.0001122	1.42E-04	DKFZp434J0315;LOC81691;FEN1
GO:0042288 MHC class I protein binding	2	0.0001162	1.45E-04	TUBB2C;TUBB2A

GO:0016717 oxidoreductase activity, acting on paired donors, with oxidation of a pair of donors resulting in the reduction of molecular oxygen to two molecules of water	2	0.0001985	2.32E-04	FADS2;Q9H3G3_HUMAN_hsa-homolog
GO:0030374 ligand-dependent nuclear receptor transcription coactivator activity	2	0.0001985	2.32E-04	HMGA1;RBM14
GO:0046982 protein heterodimerization activity	3	0.0002807	3.03E-04	SMC4L1;BARD1;KATNA1
GO:0003777 microtubule motor activity	2	0.0003482	3.58E-04	KIF23;KIF2C
GO:0005506 iron ion binding	5	0.000373	3.73E-04	CYP11A1;FADS2;Q9H3G3_HUMAN_hsa-homolog;P4HA2;P4HA1
GO:0017111 nucleoside-triphosphatase activity	6	0.0005433	5.24E-04	RFC4;MCM7;Q9H4N9_HUMAN_hsa-homolog;TRIP13;RAD51;KATNA1
GO:0015269 calcium-activated potassium channel activity	2	0.0007406	7.02E-04	SK3;KCNN3
GO:0005200 structural constituent of cytoskeleton	2	0.0007489	7.04E-04	MAPT;DSP
GO:0016706 oxidoreductase activity, acting on paired donors, with incorporation or reduction of molecular oxygen, 2-oxoglutarate as one donor, and incorporation of one atom each of oxygen into both donors	2	0.0008319	7.66E-04	P4HA2;P4HA1

GO:0005520 insulin-like growth factor binding	2	0.0011365	1.01E-03	WISP2;IGFBP4
GO:0008408 3'-5' exonuclease activity	2	0.0014867	1.26E-03	RAD50;POLD1
GO:0043565 sequence-specific DNA binding	5	0.0016602	1.39E-03	ATF5;TGIF;POU3F1;LHX2;LHX8
GO:0016251 general RNA polymerase II transcription factor activity	2	0.0018819	1.56E-03	TAF1A;GTF2F2
GO:0003725 double-stranded RNA binding	2	0.0023214	1.74E-03	ILF3;ILF2
GO:0008386 cholesterol monooxygenase (side-chain-cleaving) activity	1	0.0023663	1.74E-03	CYP11A1
GO:0008061 chitin binding	1	0.0023663	1.74E-03	CHIT1
GO:0004229 gelatinase B activity	1	0.0023663	1.74E-03	MMP9
GO:0019131 tripeptidyl-peptidase I activity	1	0.0023663	1.74E-03	TPP1
GO:0042975 peroxisome proliferator activated receptor binding	1	0.0023663	0.0017411	HMGA1
GO:0005171 hepatocyte growth factor receptor binding	1	0.0023663	0.0017411	GLMN
GO:0043142 single-stranded DNA-dependent ATPase activity	1	0.0023663	0.0017411	RAD51
GO:0043221 SMC protein binding	1	0.0023663	0.0017411	POLS

GO:0008568 microtubule-severing ATPase activity	1	0.0023663	0.0017411	KATNA1
GO:0003999 adenine phosphoribosyltransferase activity	1	0.0023663	0.0017411	APRT
GO:0020037 heme binding	3	0.0025918	0.0017491	CYP11A1;FADS2;Q9H3G3_HUMAN_hsa-homolog
GO:0016702 oxidoreductase activity, acting on single donors with incorporation of molecular oxygen, incorporation of two atoms of oxygen	2	0.0026388	0.0017699	P4HA2;P4HA1
GO:0004842 ubiquitin-protein ligase activity	2	0.0030666	0.002026	BARD1;UBE2C
GO:0016491 oxidoreductase activity	6	0.0040538	0.0026077	FADS2;P4HA2;HSD17B1;P4HA1;Q8N9Z6_HUMAN_hsa-homolog;Q9H3G3_HUMAN_hsa-homolog
GO:0003682 chromatin binding	2	0.0045099	0.0027806	SMARCC1;DKFZp564E2182
GO:0042803 protein homodimerization activity	2	0.0047226	0.0027806	BARD1;CEP57
GO:0016286 small conductance calcium-activated potassium channel activity	1	0.0047269	0.0027806	SK3
GO:0004909 interleukin-1, Type I, activating receptor activity	1	0.0047269	0.0027806	IL1R1

GO:0000014 single-stranded DNA specific endodeoxyribonuclease activity	1	0.0047269	0.0027806	RAD50
GO:0003680 AT DNA binding	1	0.0047269	0.0027806	HMGA1
GO:0046965 retinoid X receptor binding	1	0.0047269	0.0027806	HMGA1
GO:0008309 double-stranded DNA specific exodeoxyribonuclease activity	1	0.0047269	0.0027806	FEN1
GO:0019237 centromeric DNA binding	1	0.0047269	0.0027806	KIF2C
GO:0004053 arginase activity	1	0.0047269	0.0027806	ARG2
GO:0017106 activin inhibitor activity	1	0.0047269	0.0027806	FST
GO:0005011 macrophage colony stimulating factor receptor activity	1	0.0047269	0.0027806	CSF1R
GO:0004814 arginine-tRNA ligase activity	1	0.0047269	0.0027806	RARS
GO:0004820 glycine-tRNA ligase activity	1	0.0047269	0.0027806	POLG2
GO:0030674 protein binding, bridging	2	0.0056185	0.0030748	RAD50;RBM14
GO:0005216 ion channel activity	2	0.0059257	0.0032269	SK3;KCNN3
GO:0008201 heparin binding	2	0.0060933	0.0033018	MDK;THBS1
GO:0004948 calcitonin receptor activity	1	0.0070821	0.003469	CALCRL

GO:0003701 RNA polymerase I transcription factor activity	1	0.0070821	0.003469	TAF1A
GO:0046848 hydroxyapatite binding	1	0.0070821	0.003469	BGLAP
GO:0016299 regulator of G-protein signaling activity	1	0.0070821	0.003469	RGS7
GO:0003891 delta DNA polymerase activity	1	0.0070821	0.003469	POLD1
GO:0003895 gamma DNA-directed DNA polymerase activity	1	0.0070821	0.003469	POLG2
GO:0017134 fibroblast growth factor binding	1	0.0070821	0.003469	CEP57
GO:0003702 RNA polymerase II transcription factor activity	3	0.0077462	0.0037537	ATF5;ZNF354A;ILF2
GO:0008133 collagenase activity	1	0.0094316	0.0043683	MMP9
GO:0005005 transmembrane-ephrin receptor activity	1	0.0094316	0.0043683	EFNB3
GO:0043027 caspase inhibitor activity	1	0.0094316	0.0043683	BIRC5
GO:0016538 cyclin-dependent protein kinase regulator activity	1	0.0094316	0.0043683	CKS2
GO:0008143 poly(A) binding	1	0.0094316	0.0043683	TIA1
GO:0003714 transcription corepressor activity	2	0.0099021	0.0044458	ATF5;TGIF
GO:0008565 protein transporter activity	2	1.05E-02	4.70E-03	RAMP3;KPNA2

*Q-value, false discovery
rate-adjusted p-value.

Supplemental Table S3. KEGG pathways of genes differentially expressed in rhesus monkey ovary as a function of age

**Pathway_KEGG**	Number	p-Value	q-Value*	Genes
Cell cycle	11	2.97E-15	2.4E-14	PLK1_hsa-homolog;MAD2L1_hsa-homolog;MCM7_hsa-homolog;CHEK1_hsa-homolog;MCM3_hsa-homolog;BUB1B_hsa-homolog;CDC20_hsa-homolog;CDC2_hsa-homolog;CCNB2_hsa-homolog;BUB1_hsa-homolog;HDAC2_hsa-homolog
Gap junction	10	2.23E-13	1.3E-12	LOC574113;TUBB2C_hsa-homolog;LOC707215;LOC718034;CDC2_hsa-homolog;LOC715011;LOC715011;LOC574113;TUBB_hsa-homolog;LOC574113;TUBA2;K-ALPHA-1_hsa-homolog;LOC574113;CSNK1A1_hsa-homolog
Arginine and proline metabolism	4	2.16E-05	3.2E-05	P4HA2_hsa-homolog;ARG2_hsa-homolog;RARS_hsa-homolog;P4HA1_hsa-homolog
DNA polymerase	3	4.11E-05	5.8E-05	POLS_hsa-homolog;POLD1_hsa-homolog;POLG2_hsa-homolog
MAPK signaling pathway	6	6.10E-05	8.1E-05	RAP1A_hsa-homolog;IL1R1_hsa-homolog;CASP2_hsa-homolog;MAPT;MAPT_hsa-homolog;MAX_hsa-homolog
Tyrosine metabolism	3	4.50E-04	0.00046	AOX1_hsa-homolog;AOX1_hsa-homolog;DBH_hsa-homolog;PRMT5_hsa-homolog
TGF-beta signaling pathway	3	1.23E-03	0.00118	FST_hsa-homolog;AMH_hsa-homolog;THBS1_hsa-homolog
Pyrimidine metabolism	3	1.35E-03	0.00124	TYMS_hsa-homolog;TYMS_hsa-homolog;POLD1_hsa-homolog;CAD_hsa-homolog
Ubiquitin mediated proteolysis	2	3.48E-03	0.00273	CDC20_hsa-homolog;UBE2C_hsa-homolog
Leukocyte transendothelial migration	3	4.27E-03	0.00315	MMP9_hsa-homolog;THY1_hsa-homolog;RAP1A_hsa-homolog
Androgen and estrogen metabolism	2	6.59E-03	0.00467	HSD17B1_hsa-homolog;PRMT5_hsa-homolog

*Q-value, false discovery rate-adjusted p-value.

Supplemental Table S4. Human-*M. mulatta* orthologous genes

Ensembl Gene ID	Human Ensembl Gene ID	% Identity with respect to query gene	dN*	% Identity with respect to human gene	dS#	Homology Type	Associated Gene Name	Marks
ENSMMUG00000010240	ENSG00000169442	88	0.0599	87	0.1807	ortholog_one2one	*CD52*	
ENSMMUG00000005832	ENSG00000150048	90	0.0526	90	0.078	ortholog_one2one	*CLEC1A*	
ENSMMUG00000002200	ENSG00000162407	95	0.0402	95	0.0934	ortholog_one2one	*PPAP2B*	
ENSMMUG00000013666	ENSG00000139117	99	0.0024	99	0.0673	ortholog_one2one	*CPNE8*	
ENSMMUG00000012661	ENSG00000121440	99	0.0039	99	0.1251	ortholog_one2one	*PDZRN3*	
ENSMMUG00000012487	ENSG00000144724	99	0.0073	99	0.0423	ortholog_one2one	*PTPRG*	
ENSMMUG00000014486	ENSG00000116473	100	0	100	0.0263	ortholog_one2one	*RAP1A*	
ENSMMUG00000020979	ENSG00000185614	97	0.0165	95	0.0788	ortholog_one2one	*FAM212A*	
ENSMMUG00000016976	ENSG00000107099	99	0.0064	95	0.0747	ortholog_one2one	*DOCK8*	
ENSMMUG00000017793	ENSG00000143603	96	0.0256	96	0.1153	ortholog_one2one	*KCNN3*	
ENSMMUG00000006576	ENSG00000163346	92	0.0371	93	0.1011	ortholog_one2one	*PBXIP1*	
ENSMMUG00000002248	ENSG00000106991	94	0.0262	55	0.1097	ortholog_one2one	*ENG*	
ENSMMUG00000023335	ENSG00000082996	93	0.0508	92	0.1232	ortholog_one2one	*RNF13*	
ENSMMUG00000021232	ENSG00000152137	100	0	100	0.0712	ortholog_one2one	*HSPB8*	
ENSMMUG00000013004	ENSG00000127241	97	0.0165	97	0.0834	ortholog_one2one	*MASP1*	
ENSMMUG00000006442	ENSG00000158445	100	0.0011	100	0.06	ortholog_one2one	*KCNB1*	
ENSMMUG00000008709	ENSG00000133063	95	0.0257	95	0.0971	ortholog_one2one	*CHIT1*	
ENSMMUG00000016549	ENSG00000100985	94	0.026	94	0.0858	ortholog_one2one	*MMP9*	
ENSMMUG00000009448	ENSG00000003436	93	0.0314	93	0.0509	ortholog_one2one	*TFPI*	
ENSMMUG00000010605	ENSG00000064205	96	0.0191	60	0.1266	ortholog_one2one	*WISP2*	
ENSMMUG00000010602	ENSG00000168734	99	0.0062	99	0.0666	ortholog_one2one	*PKIG*	
ENSMMUG00000001475	ENSG00000144339	100	0.0014	100	0.0245	ortholog_one2one	*TMEFF2*	
ENSMMUG00000021931	ENSG00000138356	96	0.0226	96	0.0627	ortholog_one2one	*AOX1*	
ENSMMUG00000016447	ENSG00000101335	100	0	95	0.0735	ortholog_one2one	*MYL9*	
ENSMMUG00000008652	ENSG00000175602	100	0.0021	100	0.1636	ortholog_one2one	*CCDC85B*	
ENSMMUG00000000327	ENSG00000134824	98	0.0091	98	0.0977	ortholog_one2one	*FADS2*	
ENSMMUG00000013798	ENSG00000149485	89	0.0456	89	0.1117	ortholog_one2one	*FADS1*	

ENSMMUG00000007745	ENSG00000166340	98	0.0079	98	0.0512	ortholog_one2one	*TPP1*	
ENSMMUG00000008923	ENSG00000154096	99	0.0052	99	0.1051	ortholog_one2one	*THY1*	
ENSMMUG00000005391	ENSG00000119729	100	0	78	0.0064	ortholog_one2one	*RHOQ*	
ENSMMUG00000019416	ENSG00000042445	95	0.0292	95	0.0823	ortholog_one2many	*RETSAT*	
ENSMMUG00000020737	ENSG00000115594	95	0.0227	95	0.0538	ortholog_one2one	*IL1R1*	
ENSMMUG00000010293	ENSG00000170262	91	0.0411	91	0.127	ortholog_one2one	*MRAP*	
ENSMMUG00000001384	ENSG00000137449	97	0.0038	55	0.0333	ortholog_one2one	*CPEB2*	
ENSMMUG00000003829	ENSG00000122679	96	0.0175	96	0.1448	ortholog_one2one	*RAMP3*	
ENSMMUG00000020322	ENSG00000109107	99	0.0027	99	0.0706	ortholog_one2one	*ALDOC*	
ENSMMUG00000021934	ENSG00000005243	98	0.0039	99	0.0561	ortholog_one2one	*COPZ2*	
ENSMMUG00000015226	ENSG00000152620	100	0.0015	63	0.0583	ortholog_one2one	*NADKD1*	
ENSMMUG00000004196	ENSG00000137463	83	0.0213	96	0.051	ortholog_one2one	*C4ORF49*	
ENSMMUG00000009384	ENSG00000141753	100	0	100	0.1277	ortholog_one2one	*IGFBP4*	
ENSMMUG00000002062	ENSG00000153113	90	0.02	85	0.0521	ortholog_one2one	*CAST*	
ENSMMUG00000013183	ENSG00000145743	87	0.0803	47	0.1501	ortholog_one2one	*FBXL17*	
ENSMMUG00000002195	ENSG00000108950	97	0.0118	96	0.1172	ortholog_one2one	*FAM20A*	
ENSMMUG00000009837	ENSG00000138593	99	0.0029	99	0.0704	ortholog_one2one	*SECISBP2L*	
ENSMMUG00000010798	ENSG00000260916	97	0.012	90	0.0467	ortholog_one2one	*CCPG1*	
ENSMMUG00000003239	ENSG00000140459	98	0.0117	98	0.0704	ortholog_one2one	*CYP11A1*	
ENSMMUG00000022243	ENSG00000244020	85	0.0829	85	0.626	ortholog_many2many		
ENSMMUG00000022303	ENSG00000003989	90	0.0593	85	0.1343	ortholog_one2one	*SLC7A2*	
ENSMMUG00000003347	ENSG00000168300	99	0.0024	99	0.032	ortholog_one2one	*PCMTD1*	
ENSMMUG00000012513	ENSG00000147592	84	0.1194	85	0.2198	ortholog_one2one	*LACTB2*	
ENSMMUG00000028749	ENSG00000130208	86	0.0676	67	0.1655	ortholog_one2one	*APOC1*	
ENSMMUG00000013067	ENSG00000142227	99	0.0026	99	0.17	ortholog_one2one	*EMP3*	
ENSMMUG00000000070	ENSG00000087586	98	0.0121	97	0.0523	ortholog_one2one	*AURKA*	*co-down*
ENSMMUG00000000416	ENSG00000203907	95	0.0248	86	0.1074	ortholog_one2one	*OOEP*	*co-down*
ENSMMUG00000002425	ENSG00000165588	99	0.0029	99	0.0155	ortholog_one2one	*OTX2*	*co-down*
ENSMMUG00000002875	ENSG00000102466	100	0	100	0.0252	ortholog_one2one	*FGF14*	*co-down*
ENSMMUG00000002899	ENSG00000162613	98	0.0014	100	0.0631	ortholog_one2one	*FUBP1*	*co-down*
ENSMMUG00000003637	ENSG00000188372	80	0.0675	86	0.1479	ortholog_one2one	*ZP3*	*co-down*
ENSMMUG00000004579	ENSG00000051180	95	0.0237	95	0.057	ortholog_one2one	*RAD51*	*co-down*

ENSMMUG00000004839	ENSG00000136231	99	0.0008	100	0.035	ortholog_one2one	*IGF2BP3*	*co-down*
ENSMMUG00000005478	ENSG00000137574	94	0.0295	94	0.0622	ortholog_one2one	*TGS1*	*co-down*
ENSMMUG00000005700	ENSG00000138376	95	0.0219	95	0.0777	ortholog_one2one	*BARD1*	*co-down*
ENSMMUG00000006704	ENSG00000182223	83	0.0956	83	0.1686	ortholog_one2one	*ZAR1*	*co-down*
ENSMMUG00000007887	ENSG00000135486	99	0.0034	71	0.0406	ortholog_one2many		*co-down*
ENSMMUG00000007988	ENSG00000179873	83	0.0939	75	0.0988	ortholog_one2one	*NLRP11*	*co-down*
ENSMMUG00000008284	ENSG00000165506	92	0.0343	93	0.0991	ortholog_one2one	*DNAAF2*	*co-down*
ENSMMUG00000008458	ENSG00000175691	95	0.0244	94	0.0951	ortholog_one2one	*ZNF77*	*co-down*
ENSMMUG00000008526	ENSG00000111875	100	0	100	0.0202	ortholog_one2one	*ASF1A*	*co-down*
ENSMMUG00000009256	ENSG00000153574	100	0.0015	100	0.0544	ortholog_one2one	*RPIA*	*co-down*
ENSMMUG00000009775	ENSG00000241697	98	0.0062	99	0.0259	ortholog_one2one	*TMEFF1*	*co-down*
ENSMMUG00000010038	ENSG00000107105	93	0	100	0.0287	ortholog_one2one	*ELAVL2*	*co-down*
ENSMMUG00000010393	ENSG00000062038	96	0.0195	90	0.1048	ortholog_one2one	*CDH3*	*co-down*
ENSMMUG00000010582	ENSG00000184661	90	0.0577	90	0.1457	ortholog_one2one	*CDCA2*	*co-down*
ENSMMUG00000010745	ENSG00000147655	98	0.012	98	0.0315	ortholog_one2one	*RSPO2*	*co-down*
ENSMMUG00000010852	ENSG00000117148	98	0.0108	98	0.1087	ortholog_one2one	*ACTL8*	*co-down*
ENSMMUG00000011191	ENSG00000013810	81	0.1321	79	0.1982	ortholog_one2one	*TACC3*	*co-down*
ENSMMUG00000011479	ENSG00000125871	96	0.0148	96	0.072	ortholog_one2one	*C10H20ORF72*	*co-down*
ENSMMUG00000012164	ENSG00000188229	100	0	100	0.1328	ortholog_one2one	*TUBB2C*	*co-down*
ENSMMUG00000012176	ENSG00000151725	75	0.1784	76	0.317	ortholog_one2one	*MLF1IP*	*co-down*
ENSMMUG00000012679	ENSG00000104147	95	0.027	95	0.0668	ortholog_one2one	*OIP5*	*co-down*
ENSMMUG00000012991	ENSG00000139675	93	0.0347	86	0.0633	ortholog_one2one	*HNRNPA1L2*	*co-down*
ENSMMUG00000013565	ENSG00000119772	99	0.0039	99	0.062	ortholog_one2one	*DNMT3A*	*co-down*
ENSMMUG00000014030	ENSG00000138668	100	0	100	0.0112	ortholog_one2one	*HNRNPD*	*co-down*
ENSMMUG00000014614	ENSG00000166800	98	0.0105	98	0.0523	ortholog_one2one	*LDHAL6A*	*co-down*
ENSMMUG00000015838	ENSG00000213231	86	0.0486	91	0.1588	ortholog_one2one	*TCL1B*	*co-down*
ENSMMUG00000015865	ENSG00000081181	99	0.0055	99	0.0533	ortholog_one2one	*ARG2*	*co-down*
ENSMMUG00000016742	ENSG00000137770	100	0.001	100	0.0172	ortholog_one2one	*CTDSPL2*	*co-down*
ENSMMUG00000017174	ENSG00000152455	100	0.0013	85	0.0504	ortholog_one2many	*SUV39H2*	*co-down*
ENSMMUG00000016759	ENSG00000103310	94	0.0274	94	0.0717	ortholog_one2one	*ZP2*	*co-down*
ENSMMUG00000017372	ENSG00000137875	86	0.0787	86	0.2149	ortholog_one2one	*BCL2L10*	*co-down*
ENSMMUG00000017478	ENSG00000088992	100	0.002	100	0.0634	ortholog_one2one	*TESC*	*co-down*

ENSMMUG00000017653	ENSG00000169679	96	0.0183	96	0.0484	ortholog_one2one	*BUB1*	*co-down*
ENSMMUG00000018070	ENSG00000109805	95	0.0323	94	0.081	ortholog_one2one	*NCAPG*	*co-down*
ENSMMUG00000018142	ENSG00000134057	99	0.0069	99	0.0306	ortholog_one2one	*CCNB1*	*co-down*
ENSMMUG00000018907	ENSG00000178752	84	0.0678	33	0.3648	ortholog_one2one	*FAM132B*	*co-down*
ENSMMUG00000020207	ENSG00000112742	97	0.015	94	0.0641	ortholog_one2one	*TTK*	*co-down*
ENSMMUG00000020594	ENSG00000131153	99	0.0052	99	0.0739	ortholog_one2one	*GINS2*	*co-down*
ENSMMUG00000020852	ENSG00000183733	92	0.0399	92	0.0923	ortholog_one2one	*FIGLA*	*co-down*
ENSMMUG00000022050	ENSG00000124575	95	0.0313	95	0.119	ortholog_one2one	*HIST1H1D*	*co-down*
ENSMMUG00000021149	ENSG00000112118	100	0.0023	94	0.0608	ortholog_one2one	*MCM3*	*co-down*
ENSMMUG00000022413	ENSG00000161547	100	0.0039	100	0.1424	ortholog_one2one	*SRSF2*	*co-down*
ENSMMUG00000023077	ENSG00000164611	99	0.0076	99	0.0368	ortholog_one2one	*PTTG1*	*co-down*
ENSMMUG00000023151	ENSG00000163808	98	0.011	98	0.0422	ortholog_one2one	*KIF15*	*co-down*
ENSMMUG00000023269	ENSG00000121152	93	0.0454	92	0.1927	ortholog_one2one	*NCAPH*	*co-down*
ENSMMUG00000023393	ENSG00000145220	97	0.012	97	0.0823	ortholog_one2one	*LYAR*	*co-down*
ENSMMUG00000028711	ENSG00000160505	92	0.0432	92	0.1159	ortholog_one2one	*NLRP4*	*co-down*
ENSMMUG00000029234	ENSG00000175643	99	0.0059	99	0.1231	ortholog_one2one	*RMI2*	*co-down*
ENSMMUG00000029400	ENSG00000112290	100	0.0024	38	0.0505	ortholog_one2many		*co-down*
ENSMMUG00000029628	ENSG00000157456	90	0.0585	91	0.1476	ortholog_one2one	*CCNB2*	*co-down*
ENSMMUG00000030305	ENSG00000211891	85	0.0795	85	0.1607	ortholog_one2one	*IGHE*	*co-down*
ENSMMUG00000031962	ENSG00000187569	76	0.1175	79	0.0891	ortholog_one2one	*DPPA3*	*co-down*
ENSMMUG00000000135	ENSG00000106688	98	0.0073	98	0.0602	ortholog_one2one	*SLC1A1*	
ENSMMUG00000000262	ENSG00000100024	97	0.0108	98	0.0966	ortholog_one2one	*UPB1*	
ENSMMUG00000000299	ENSG00000104899	98	0.0099	29	0.212	ortholog_one2one	*AMH*	
ENSMMUG00000000308	ENSG00000116497	94	0.0292	94	0.0523	ortholog_one2one	*S100PBP*	
ENSMMUG00000000500	ENSG00000164694	90	0.0576	88	0.1364	ortholog_one2one	*FNDC1*	
ENSMMUG00000000567	ENSG00000095739	98	0.0074	98	0.0503	ortholog_one2one	*BAMBI*	
ENSMMUG00000000664	ENSG00000133818	100	0	100	0.0287	ortholog_one2one	*RRAS2*	
ENSMMUG00000000704	ENSG00000113569	96	0.0256	52	0.1089	ortholog_one2one	*NUP155*	
ENSMMUG00000000823	ENSG00000113368	99	0.0033	99	0.0519	ortholog_one2one	*LMNB1*	
ENSMMUG00000000824	ENSG00000140873	92	0.0551	92	0.1171	ortholog_one2one	*ADAMTS18*	
ENSMMUG00000000835	ENSG00000102854	83	0.0887	83	0.1992	ortholog_one2one	*MSLN*	
ENSMMUG00000000929	ENSG00000105146	95	0.023	95	0.049	ortholog_one2one	*AURKC*	

ENSMMUG00000000966	ENSG00000088325	97	0.0175	97	0.0579	ortholog_one2one	*TPX2*
ENSMMUG00000000974	ENSG00000095002	99	0.0065	91	0.0712	ortholog_one2one	*MSH2*
ENSMMUG00000000979	ENSG00000206053	94	0.0307	94	0.0773	ortholog_one2one	*HN1L*
ENSMMUG00000001035	ENSG00000100462	98	0.013	95	0.089	ortholog_one2one	*PRMT5*
ENSMMUG00000001125	ENSG00000185917	98	0.0113	98	0.0606	ortholog_one2one	*SETD4*
ENSMMUG00000001126	ENSG00000116001	100	0.0012	100	0.0246	ortholog_one2one	*TIA1*
ENSMMUG00000001172	ENSG00000120669	89	0.0647	75	0.1152	ortholog_one2many	
ENSMMUG00000001201	ENSG00000090061	95	0	67	0.0689	ortholog_one2one	*CCNK*
ENSMMUG00000001269	ENSG00000141219	93	0.0391	93	0.0619	ortholog_one2one	*C16H17orf80*
ENSMMUG00000001329	ENSG00000012048	93	0.0332	92	0.0617	ortholog_one2one	*BRCA1*
ENSMMUG00000001359	ENSG00000110888	94	0.0095	98	0.0412	ortholog_one2one	*CAPRIN2*
ENSMMUG00000001513	ENSG00000163637	100	0.0011	100	0.0818	ortholog_one2one	*PRICKLE2*
ENSMMUG00000001526	ENSG00000136891	100	0.0011	100	0.029	ortholog_one2one	*TEX10*
ENSMMUG00000001758	ENSG00000113712	100	0	100	0.0263	ortholog_one2one	*CSNK1A1*
ENSMMUG00000001856	ENSG00000196419	94	0.0464	94	0.1089	ortholog_one2one	*XRCC6*
ENSMMUG00000001858	ENSG00000170144	90	0.0414	83	0.0746	ortholog_one2many	
ENSMMUG00000001930	ENSG00000198182	97	0.015	97	0.0834	ortholog_one2one	*ZNF607*
ENSMMUG00000002010	ENSG00000158796	100	0.0016	100	0.0317	ortholog_one2one	*DEDD*
ENSMMUG00000002034	ENSG00000158806	95	0.0222	95	0.0458	ortholog_one2one	*NPM2*
ENSMMUG00000002066	ENSG00000132600	91	0.0562	75	0.1423	ortholog_one2one	*PRMT7*
ENSMMUG00000002105	ENSG00000116996	92	0.0433	82	0.0961	ortholog_one2one	*ZP4*
ENSMMUG00000002109	ENSG00000120685	96	0.0176	96	0.0641	ortholog_one2one	*C13ORF23*
ENSMMUG00000002163	ENSG00000170421	99	0.0051	94	0.1291	ortholog_one2many	*KRT8*
ENSMMUG00000002257	ENSG00000058600	96	0.0289	96	0.1505	ortholog_one2one	*POLR3E*
ENSMMUG00000002460	ENSG00000031691	95	0.021	54	0.0642	ortholog_one2one	*CENPQ*
ENSMMUG00000002620	ENSG00000160783	87	0.0723	71	0.137	ortholog_one2many	
ENSMMUG00000002621	ENSG00000183856	97	0.0123	98	0.0567	ortholog_one2one	*IQGAP3*
ENSMMUG00000002838	ENSG00000132823	98	0.0081	98	0.0463	ortholog_one2one	*C20ORF111*
ENSMMUG00000002912	ENSG00000104953	90	0.0528	68	0.2366	ortholog_one2one	*TLE6*
ENSMMUG00000003041	ENSG00000167306	97	0.0173	97	0.0865	ortholog_one2one	*MYO5B*
ENSMMUG00000003043	ENSG00000160953	90	0.0475	90	0.1289	ortholog_one2one	*MUM1*
ENSMMUG00000003047	ENSG00000071626	100	0.0012	100	0.0683	ortholog_one2one	*DAZAP1*

ENSMMUG00000003102	ENSG00000059573	98	0.0089	98	0.0547	ortholog_one2one	*ALDH18A1*
ENSMMUG00000003130	ENSG00000113522	99	0.0028	99	0.0408	ortholog_one2one	*RAD50*
ENSMMUG00000003136	ENSG00000134046	98	0.0094	98	0.0223	ortholog_one2one	*MBD2*
ENSMMUG00000003149	ENSG00000117399	100	0.0019	100	0.0683	ortholog_one2one	*CDC20*
ENSMMUG00000003247	ENSG00000161944	60	0.2733	33	2.5162	ortholog_many2man	*ASGR1*
ENSMMUG00000003324	ENSG00000167183	99	0.0048	99	0.0882	ortholog_one2one	*PRR15L*
ENSMMUG00000003331	ENSG00000196812	98	0.0105	98	0.0788	ortholog_one2one	*ZSCAN16*
ENSMMUG00000003371	ENSG00000133119	100	0.0014	100	0.0527	ortholog_one2one	*RFC3*
ENSMMUG00000003384	ENSG00000101447	97	0.013	92	0.099	ortholog_one2one	*FAM83D*
ENSMMUG00000003442	ENSG00000162664	98	0.0091	98	0.0486	ortholog_one2one	*ZNF326*
ENSMMUG00000003592	ENSG00000121578	97	0.0157	97	0.0758	ortholog_one2one	*B4GALT4*
ENSMMUG00000003625	ENSG00000119599	97	0.0151	95	0.0878	ortholog_one2one	*DCAF4*
ENSMMUG00000003654	ENSG00000103275	77	0.0813	89	0.0999	ortholog_one2one	*UBE2I*
ENSMMUG00000003671	ENSG00000106689	100	0.001	100	0.0897	ortholog_one2one	*LHX2*
ENSMMUG00000003691	ENSG00000118620	96	0.0156	96	0.0334	ortholog_one2one	*ZNF430*
ENSMMUG00000003701	ENSG00000159147	97	0.0153	96	0.0591	ortholog_one2one	*DONSON*
ENSMMUG00000003704	ENSG00000163918	98	0.0092	98	0.0842	ortholog_one2one	*RFC4*
ENSMMUG00000003709	ENSG00000146410	94	0.0259	94	0.0578	ortholog_one2one	*FAM54A*
ENSMMUG00000003710	ENSG00000029363	95	0.0322	95	0.0778	ortholog_one2one	*BCLAF1*
ENSMMUG00000003782	ENSG00000165304	96	0.0216	96	0.0765	ortholog_one2one	*MELK*
ENSMMUG00000003791	ENSG00000112877	93	0.0339	93	0.1078	ortholog_one2one	*CEP72*
ENSMMUG00000003915	ENSG00000183207	100	0	100	0.1722	ortholog_one2one	*RUVBL2*
ENSMMUG00000003922	ENSG00000001497	90	0.0635	90	0.1234	ortholog_one2one	*LAS1L*
ENSMMUG00000004009	ENSG00000178814	98	0.0101	95	0.1488	ortholog_one2one	*OPLAH*
ENSMMUG00000004021	ENSG00000166526	94	0.0413	93	0.1586	ortholog_one2one	*ZNF3*
ENSMMUG00000004034	ENSG00000185798	98	0.0102	79	0.072	ortholog_one2one	*WDR53*
ENSMMUG00000004046	ENSG00000176890	98	0.0105	98	0.0997	ortholog_one2one	*TYMS*
ENSMMUG00000004063	ENSG00000128805	97	0.0123	95	0.1397	ortholog_one2one	*ARHGAP22*
ENSMMUG00000004122	ENSG00000186868	94	0.0313	94	0.0874	ortholog_one2one	*MAPT*
ENSMMUG00000004132	ENSG00000093000	97	0.0144	97	0.0852	ortholog_one2one	*NUP50*
ENSMMUG00000004255	ENSG00000119596	99	0.0063	84	0.0369	ortholog_one2one	*YLPM1*
ENSMMUG00000004341	ENSG00000125952	100	0	100	0.0063	ortholog_one2one	*MAX*

ENSMMUG00000004346	ENSG00000154040	40	0.4964	51	0.7148	ortholog_one2one	*CABYR*
ENSMMUG00000004374	ENSG00000258677	23	0.8375	22	71.6004	ortholog_many2many	
ENSMMUG00000004460	ENSG00000042088	97	0.0143	97	0.051	ortholog_one2one	*TDP1*
ENSMMUG00000004646	ENSG00000186625	99	0.0049	78	0.0453	ortholog_one2one	*KATNA1*
ENSMMUG00000004706	ENSG00000085552	97	0.0085	98	0.0647	ortholog_one2one	*IGSF9*
ENSMMUG00000004725	ENSG00000136450	100	0	100	0	ortholog_one2one	*SRSF1*
ENSMMUG00000004875	ENSG00000164109	100	0	100	0.0283	ortholog_one2one	*MAD2L1*
ENSMMUG00000005076	ENSG00000148459	97	0.016	97	0.0688	ortholog_one2one	*PDSS1*
ENSMMUG00000005086	ENSG00000177426	93	0.0314	67	0.1157	ortholog_one2one	*TGIF1*
ENSMMUG00000005237	ENSG00000135093	100	0.0018	100	0.0603	ortholog_one2one	*USP30*
ENSMMUG00000005278	ENSG00000169857	95	0.0252	93	0.0761	ortholog_one2one	*AVEN*
ENSMMUG00000005287	ENSG00000162624	99	0.0039	99	0.0525	ortholog_one2one	*LHX8*
ENSMMUG00000005602	ENSG00000065328	95	0.0328	94	0.1173	ortholog_one2one	*MCM10*
ENSMMUG00000005635	ENSG00000135829	99	0.0018	100	0.057	ortholog_one2one	*DHX9*
ENSMMUG00000005674	ENSG00000213066	98	0.0093	98	0.0728	ortholog_one2one	*FGFR1OP*
ENSMMUG00000005768	ENSG00000130826	99	0.0027	99	0.056	ortholog_one2one	*DKC1*
ENSMMUG00000005856	ENSG00000171766	97	0.0133	97	0.0331	ortholog_one2one	*GATM*
ENSMMUG00000005883	ENSG00000266953	39	0.186	67	0.2286	ortholog_one2many	
ENSMMUG00000005988	ENSG00000165105	95	0.0232	95	0.0842	ortholog_one2one	*RASEF*
ENSMMUG00000006018	ENSG00000076382	92	0.0513	92	0.069	ortholog_one2one	*SPAG5*
ENSMMUG00000006395	ENSG00000099284	100	0.0013	100	0.0467	ortholog_one2one	*H2AFY2*
ENSMMUG00000006461	ENSG00000165804	96	0.0186	87	0.0693	ortholog_one2one	*ZNF219*
ENSMMUG00000006548	ENSG00000122778	95	0.0244	91	0.0898	ortholog_one2one	*KIAA1549*
ENSMMUG00000006598	ENSG00000254772	100	0.0011	100	0.0477	ortholog_one2one	*EEF1G*
ENSMMUG00000006616	ENSG00000049759	91	0.0414	92	0.1346	ortholog_one2one	*NEDD4L*
ENSMMUG00000006657	ENSG00000168496	99	0.0036	99	0.0387	ortholog_one2one	*FEN1*
ENSMMUG00000006668	ENSG00000075886	100	0	100	0.1102	ortholog_one2many	
ENSMMUG00000006742	ENSG00000124193	88	0.0869	86	0.1086	ortholog_one2many	
ENSMMUG00000007041	ENSG00000177054	91	0.0587	91	0.0897	ortholog_one2one	*HIP14L*
ENSMMUG00000007135	ENSG00000140153	98	0	95	0.0287	ortholog_one2one	*WDR20*
ENSMMUG00000007141	ENSG00000054267	99	0.0058	96	0.0396	ortholog_one2one	*ARID4B*
ENSMMUG00000007268	ENSG00000172009	85	0.0958	83	0.5332	ortholog_one2one	*THOP1*

ENSMMUG00000007380	ENSG00000071894	97	0.0157	97	0.2578	ortholog_one2one	*CPSF1*
ENSMMUG00000007394	ENSG00000109674	92	0.0366	92	0.0448	ortholog_one2one	*NEIL3*
ENSMMUG00000007497	ENSG00000090621	100	0.0007	100	0.0382	ortholog_one2one	*PABPC4*
ENSMMUG00000007584	ENSG00000134443	99	0.0043	67	0.015	ortholog_one2one	*GRP*
ENSMMUG00000007622	ENSG00000198331	97	0.0133	97	0.0335	ortholog_one2one	*HYLS1*
ENSMMUG00000007681	ENSG00000077800	97	0.0177	97	0.0824	ortholog_one2one	*FKBP6*
ENSMMUG00000007760	ENSG00000114107	96	0.019	96	0.0323	ortholog_one2one	*CEP70*
ENSMMUG00000007830	ENSG00000239306	99	0.003	99	0.0323	ortholog_one2one	*RBM14*
ENSMMUG00000007896	ENSG00000143498	90	0.0666	89	0.0713	ortholog_one2one	*TAF1A*
ENSMMUG00000007960	ENSG00000127561	75	0.1793	68	0.8341	ortholog_one2one	*SYNGR3*
ENSMMUG00000007962	ENSG00000035141	94	0.0028	99	0.1314	ortholog_one2many	*FAM136A*
ENSMMUG00000008060	ENSG00000096654	99	0.0048	99	0.0401	ortholog_one2one	*ZNF184*
ENSMMUG00000008079	ENSG00000114638	99	0.0036	99	0.069	ortholog_one2one	*UPK1B*
ENSMMUG00000008127	ENSG00000131242	99	0.0022	87	0.1368	ortholog_one2one	*RAB11FIP4*
ENSMMUG00000008149	ENSG00000143621	100	0	100	0.0329	ortholog_one2many	*ILF2*
ENSMMUG00000008209	ENSG00000000003	90	0.0673	90	0.1069	ortholog_one2one	*TSPAN6*
ENSMMUG00000008211	ENSG00000102362	99	0.0056	99	0.0368	ortholog_one2one	*SYTL4*
ENSMMUG00000008422	ENSG00000067445	95	0.0254	89	0.0386	ortholog_one2one	*TRO*
ENSMMUG00000008557	ENSG00000157657	87	0.0031	96	0.1064	ortholog_one2one	*ZNF618*
ENSMMUG00000008585	ENSG00000158077	94	0.0313	94	0.0662	ortholog_one2one	*NLRP14*
ENSMMUG00000008611	ENSG00000080345	95	0.0238	95	0.0718	ortholog_one2one	*RIF1*
ENSMMUG00000008628	ENSG00000163380	97	0.0136	97	0.0361	ortholog_one2one	*LMOD3*
ENSMMUG00000008751	ENSG00000172943	98	0.0133	98	0.043	ortholog_one2one	*PHF8*
ENSMMUG00000008768	ENSG00000149554	98	0.0038	99	0.0357	ortholog_one2one	*CHEK1*
ENSMMUG00000008858	ENSG00000159167	100	0.0018	100	0.0414	ortholog_one2one	*STC1*
ENSMMUG00000008877	ENSG00000173876	93	0.0354	90	0.3083	ortholog_many2many	
ENSMMUG00000008921	ENSG00000036672	97	0.0154	97	0.0657	ortholog_one2one	*USP2*
ENSMMUG00000008937	ENSG00000196591	94	0.0354	94	0.1096	ortholog_one2one	*HDAC2*
ENSMMUG00000008966	ENSG00000144554	94	0.0511	32	0.0764	ortholog_one2many	
ENSMMUG00000008975	ENSG00000169131	94	0.0257	94	0.1734	ortholog_one2one	*ZNF354A*
ENSMMUG00000008976	ENSG00000178338	96	0.0158	96	0.1355	ortholog_one2one	*ZNF354B*
ENSMMUG00000009017	ENSG00000071564	89	0.0625	88	0.3073	ortholog_one2one	*TCF3*

ENSMMUG00000009030	ENSG00000122033	87	0.0692	87	0.0434	ortholog_one2one	*MTIF3*
ENSMMUG00000009133	ENSG00000108384	99	0.0075	96	0.0397	ortholog_one2one	*RAD51C*
ENSMMUG00000009157	ENSG00000089157	100	0	100	0.0384	ortholog_one2one	*RPLP0*
ENSMMUG00000009173	ENSG00000102572	100	0.0021	100	0.0882	ortholog_one2one	*STK24*
ENSMMUG00000009280	ENSG00000121058	96	0.0197	96	0.0519	ortholog_one2one	*COIL*
ENSMMUG00000009345	ENSG00000173473	85	0.1059	51	0.1488	ortholog_one2one	*SMARCC1*
ENSMMUG00000009365	ENSG00000040275	91	0.0697	90	0.1318	ortholog_one2one	*SPDL1*
ENSMMUG00000009431	ENSG00000162639	90	0.0145	95	0.0466	ortholog_one2one	*HENMT1*
ENSMMUG00000009459	ENSG00000258890	95	0.0249	95	0.0547	ortholog_one2one	*CCDC45*
ENSMMUG00000009559	ENSG00000197771	100	0.0015	99	0.0582	ortholog_one2one	*MCMBP*
ENSMMUG00000009562	ENSG00000127603	99	0.0066	54	0.0479	ortholog_one2many	
ENSMMUG00000009773	ENSG00000004487	99	0.0039	99	0.0494	ortholog_one2one	*KDM1A*
ENSMMUG00000009862	ENSG00000102445	87	0.0856	78	0.1386	ortholog_one2one	*KIAA0226L*
ENSMMUG00000009884	ENSG00000108786	95	0.0237	93	0.1329	ortholog_one2one	*HSD17B1*
ENSMMUG00000009915	ENSG00000125484	99	0.0023	100	0.051	ortholog_one2one	*GTF3C4*
ENSMMUG00000009943	ENSG00000109685	76	0.1556	36	0.3879	ortholog_one2one	*WHSC1*
ENSMMUG00000010084	ENSG00000203908	93	0.0333	88	0.0803	ortholog_one2one	*KHDC3L*
ENSMMUG00000010095	ENSG00000174842	98	0.011	98	0.0124	ortholog_one2one	*GLMN*
ENSMMUG00000010176	ENSG00000143476	98	0.0091	98	0.0412	ortholog_one2one	*DTL*
ENSMMUG00000010266	ENSG00000087460	94	0.0251	83	0.0688	ortholog_one2one	*GNAS1*
ENSMMUG00000010304	ENSG00000159079	99	0.0049	99	0.0955	ortholog_one2one	*C3H21ORF59*
ENSMMUG00000010335	ENSG00000143786	85	0.014	99	0.0938	ortholog_one2one	*CNIH3*
ENSMMUG00000010372	ENSG00000152669	96	0.0161	96	0.0753	ortholog_one2one	*CCNO*
ENSMMUG00000010417	ENSG00000141668	99	0.0059	99	0.0491	ortholog_one2one	*CBLN2*
ENSMMUG00000010423	ENSG00000101901	95	0.0261	36	0.0677	ortholog_one2one	*ALG13*
ENSMMUG00000010484	ENSG00000139436	99	0.0051	99	0.0586	ortholog_one2one	*GIT2*
ENSMMUG00000010564	ENSG00000152128	98	0.0077	98	0.0685	ortholog_one2one	*TMEM163*
ENSMMUG00000010729	ENSG00000182901	95	0.0176	97	0.0904	ortholog_one2one	*RGS7*
ENSMMUG00000010820	ENSG00000166226	100	0	100	0.0488	ortholog_one2one	*CCT2*
ENSMMUG00000010878	ENSG00000166747	93	0.0534	93	0.1268	ortholog_one2one	*AP1G1*
ENSMMUG00000010898	ENSG00000204977	98	0.0106	98	0.0501	ortholog_one2one	*TRIM13*
ENSMMUG00000010910	ENSG00000180611	100	0.0009	100	0.0555	ortholog_one2one	*MB21D2*

ENSMMUG00000011025	ENSG00000115875	100	0	100	0.0233	ortholog_one2one	*SRSF7*
ENSMMUG00000011029	ENSG00000173876	94	0.0288	94	0.284	ortholog_many2many	
ENSMMUG00000011049	ENSG00000137269	100	0.0019	100	0.0386	ortholog_one2one	*LRRC1*
ENSMMUG00000011073	ENSG00000080824	85	0.0804	50	0.3749	ortholog_one2many	*HSPCA*
ENSMMUG00000011076	ENSG00000177485	99	0.0026	99	0.035	ortholog_one2one	*ZBTB33*
ENSMMUG00000011155	ENSG00000140525	97	0.0127	93	0.0589	ortholog_one2one	*FANCI*
ENSMMUG00000011274	ENSG00000122884	95	0.0354	95	0.1082	ortholog_one2one	*P4HA1*
ENSMMUG00000011303	ENSG00000119866	92	0.0227	90	0.0744	ortholog_one2one	*BCL11A*
ENSMMUG00000011323	ENSG00000121892	96	0.0227	94	0.0781	ortholog_one2one	*PDS5A*
ENSMMUG00000011514	ENSG00000100473	100	0.0018	100	0.0395	ortholog_one2one	*COCH*
ENSMMUG00000011556	ENSG00000182578	96	0.0191	96	0.0944	ortholog_one2one	*CSF1R*
ENSMMUG00000011584	ENSG00000171316	98	0.0137	98	0.0552	ortholog_one2one	*CHD7*
ENSMMUG00000011937	ENSG00000170144	99	0.0033	72	0.0437	ortholog_one2many	
ENSMMUG00000011942	ENSG00000091651	96	0.0176	96	0.0492	ortholog_one2one	*ORC6L*
ENSMMUG00000011943	ENSG00000196345	95	0.0238	94	0.0713	ortholog_one2one	*ZNF167*
ENSMMUG00000011966	ENSG00000139970	90	0.0563	88	0.1557	ortholog_one2one	*RTN1*
ENSMMUG00000012281	ENSG00000136937	97	0.0234	97	0.0977	ortholog_one2one	*NCBP1*
ENSMMUG00000012340	ENSG00000163026	94	0.028	94	0.0637	ortholog_one2one	*C13H2orf44*
ENSMMUG00000012463	ENSG00000145833	100	0.0017	100	0.0567	ortholog_one2one	*DDX46*
ENSMMUG00000012516	ENSG00000113643	98	0.0114	98	0.0505	ortholog_one2one	*RARS*
ENSMMUG00000012534	ENSG00000123975	100	0	100	0.0107	ortholog_one2one	*CKS2*
ENSMMUG00000012546	ENSG00000173876	90	0.0452	90	0.2422	ortholog_many2many	
ENSMMUG00000012575	ENSG00000135316	100	0	100	0.022	ortholog_one2one	*SYNCRIP*
ENSMMUG00000012630	ENSG00000106144	91	0.0477	86	0.1958	ortholog_one2one	*CASP2*
ENSMMUG00000012634	ENSG00000188342	97	0.0215	97	0.0746	ortholog_one2one	*GTF2F2*
ENSMMUG00000012846	ENSG00000120438	100	0.0016	100	0.0579	ortholog_one2many	*TCP1*
ENSMMUG00000012884	ENSG00000183684	99	0.0037	99	0.0688	ortholog_one2one	*THOC4*
ENSMMUG00000012914	ENSG00000160299	89	0.0702	88	0.1676	ortholog_one2one	*PCNT*
ENSMMUG00000013125	ENSG00000177181	99	0.0025	99	0.0555	ortholog_one2one	*RIMKLA*
ENSMMUG00000013138	ENSG00000102606	95	0.0212	94	0.1023	ortholog_one2one	*ARHGEF7*
ENSMMUG00000013158	ENSG00000125386	88	0.0881	81	0.2399	ortholog_one2one	*FAM193A*
ENSMMUG00000013189	ENSG00000112941	100	0.0017	100	0.0498	ortholog_one2one	*PAPD7*

ENSMMUG00000013211	ENSG00000025772	91	0.0621	90	0.113	ortholog_one2one	*TOMM34*
ENSMMUG00000013235	ENSG00000080573	35	0.8771	31	45.0375	ortholog_many2many	
ENSMMUG00000013272	ENSG00000146013	96	0.0147	97	0.0726	ortholog_one2one	*GFRA3*
ENSMMUG00000013276	ENSG00000006453	97	0.0108	84	0.0894	ortholog_one2one	*BAIAP2L1*
ENSMMUG00000013442	ENSG00000166508	98	0.0076	98	0.1113	ortholog_one2one	*MCM7*
ENSMMUG00000013535	ENSG00000176974	98	0.0095	98	0.0671	ortholog_one2one	*SHMT1*
ENSMMUG00000013574	ENSG00000152086	99	0.0062	98	0.181	ortholog_many2many	
ENSMMUG00000013824	ENSG00000198931	94	0.027	94	0.1023	ortholog_one2one	*APRT*
ENSMMUG00000013884	ENSG00000116679	100	0.0022	100	0.0423	ortholog_one2one	*IVNS1ABP*
ENSMMUG00000013888	ENSG00000101361	99	0.0053	87	0.0403	ortholog_one2one	*NOP56*
ENSMMUG00000013892	ENSG00000120647	86	0.099	86	0.1702	ortholog_one2one	*CCDC77*
ENSMMUG00000013893	ENSG00000204576	74	0.0053	99	0.0405	ortholog_one2one	*PRR3*
ENSMMUG00000013897	ENSG00000171840	85	0.0302	94	0.1103	ortholog_one2one	*NINJ2*
ENSMMUG00000013940	ENSG00000090889	97	0.0189	96	0.0601	ortholog_one2one	*KIF4A*
ENSMMUG00000014142	ENSG00000112218	99	0.0058	99	0.0521	ortholog_one2one	*GPR63*
ENSMMUG00000014216	ENSG00000173876	96	0.0173	97	0.2535	ortholog_many2many	
ENSMMUG00000014228	ENSG00000144354	98	0.0074	98	0.0442	ortholog_one2one	*CDCA7*
ENSMMUG00000014340	ENSG00000134690	97	0.0133	97	0.0359	ortholog_one2one	*CDCA8*
ENSMMUG00000014454	ENSG00000124766	100	0	100	0.1467	ortholog_one2one	*SOX4*
ENSMMUG00000014514	ENSG00000135245	97	0.0144	97	0.1162	ortholog_one2one	*HILPDA*
ENSMMUG00000014647	ENSG00000062194	98	0.0155	98	0.1219	ortholog_one2one	*GPBP1*
ENSMMUG00000014673	ENSG00000129351	100	0.0015	99	0.0708	ortholog_one2one	*ILF3*
ENSMMUG00000014740	ENSG00000071539	99	0.0083	99	0.105	ortholog_one2one	*TRIP13*
ENSMMUG00000014753	ENSG00000178397	89	0.0567	89	0.0501	ortholog_one2one	*FAM220A*
ENSMMUG00000014784	ENSG00000096696	99	0.0057	99	0.0593	ortholog_one2one	*DSP*
ENSMMUG00000014796	ENSG00000166037	97	0.0135	97	0.0567	ortholog_one2one	*CEP57*
ENSMMUG00000014809	ENSG00000092199	100	0	100	0.0153	ortholog_one2one	*HNRNPC*
ENSMMUG00000014887	ENSG00000137807	99	0.0039	88	0.0349	ortholog_one2one	*KIF23*
ENSMMUG00000015010	ENSG00000175390	96	0.0155	97	0.094	ortholog_one2one	*EIF3F*
ENSMMUG00000015038	ENSG00000137766	97	0.0176	52	0.0984	ortholog_one2one	*UNC13C*
ENSMMUG00000015093	ENSG00000147852	99	0.0076	98	0.0478	ortholog_one2one	*VLDLR*
ENSMMUG00000015135	ENSG00000119285	95	0.0277	95	0.0811	ortholog_one2one	*HEATR1*

ENSMMUG00000015272	ENSG00000146731	96	0.0286	97	0.1258	ortholog_one2one	*CCT6A*
ENSMMUG00000015330	ENSG00000152492	95	0.0231	95	0.0455	ortholog_one2one	*CCDC50*
ENSMMUG00000015347	ENSG00000253729	98	0.0134	98	0.0693	ortholog_one2one	*PRKDC*
ENSMMUG00000015362	ENSG00000008083	99	0.0026	82	0.0723	ortholog_one2one	*JARID2*
ENSMMUG00000015375	ENSG00000143126	97	0.0181	60	0.1339	ortholog_one2one	*CELSR2*
ENSMMUG00000015403	ENSG00000153898	97	0.0124	93	0.0236	ortholog_one2one	*MCOLN2*
ENSMMUG00000015583	ENSG00000187109	99	0.0012	100	0.0353	ortholog_one2many	
ENSMMUG00000015689	ENSG00000205336	95	0.0212	96	0.1386	ortholog_one2one	*GPR56*
ENSMMUG00000015714	ENSG00000107758	100	0.0018	100	0.022	ortholog_one2one	*PPP3CB*
ENSMMUG00000015774	ENSG00000107833	99	0.0055	99	0.0602	ortholog_one2one	*NPM3*
ENSMMUG00000015906	ENSG00000112039	95	0.0273	95	0.0717	ortholog_one2one	*FANCE*
ENSMMUG00000015976	ENSG00000134253	97	0.0178	97	0.0664	ortholog_one2one	*TRIM45*
ENSMMUG00000016006	ENSG00000123892	100	0.0021	100	0.0727	ortholog_one2one	*RAB38*
ENSMMUG00000016277	ENSG00000143924	94	0.0292	94	0.0788	ortholog_one2one	*EML4*
ENSMMUG00000016292	ENSG00000087495	98	0.0085	98	0.054	ortholog_one2one	*PHACTR3*
ENSMMUG00000016450	ENSG00000011304	95	0.0384	94	0.2381	ortholog_one2one	*PTBP1*
ENSMMUG00000016530	ENSG00000136098	93	0.0187	96	0.0711	ortholog_one2one	*NEK3*
ENSMMUG00000016605	ENSG00000137075	100	0	100	0.0114	ortholog_one2one	*RNF38*
ENSMMUG00000016609	ENSG00000083635	94	0.0297	31	0.109	ortholog_one2one	*NUFIP1*
ENSMMUG00000016654	ENSG00000189159	98	0.0078	98	0.061	ortholog_one2one	*HN1*
ENSMMUG00000016685	ENSG00000182481	96	0.0181	96	0.0729	ortholog_one2many	
ENSMMUG00000016686	ENSG00000156970	93	0.0402	93	0.0673	ortholog_one2one	*BUB1B*
ENSMMUG00000016689	ENSG00000107443	100	0	73	0.0432	ortholog_one2one	*CCNJ*
ENSMMUG00000016702	ENSG00000146250	95	0.0286	94	0.0832	ortholog_one2one	*PRSS35*
ENSMMUG00000016703	ENSG00000065609	96	0.0228	86	0.0775	ortholog_one2one	*SNAP91*
ENSMMUG00000016706	ENSG00000167670	89	0.0653	88	0.2243	ortholog_one2one	*CHAF1A*
ENSMMUG00000016748	ENSG00000005189	96	0.0206	96	0.039	ortholog_one2one	
ENSMMUG00000016777	ENSG00000126733	98	0.0079	98	0.0632	ortholog_one2one	*DACH2*
ENSMMUG00000016808	ENSG00000143033	99	0.0022	99	0.0147	ortholog_one2one	*MTF2*
ENSMMUG00000017039	ENSG00000146282	98	0.0072	98	0.0461	ortholog_one2one	*RARS2*
ENSMMUG00000017045	ENSG00000136261	100	0	100	0.0272	ortholog_one2one	*BZW2*
ENSMMUG00000017199	ENSG00000174482	100	0.0016	100	0.0598	ortholog_one2one	*LINGO2*

ENSMMUG00000017255	ENSG00000119969	94	0.0049	99	0.0591	ortholog_one2one	*HELLS*
ENSMMUG00000017291	ENSG00000113810	98	0.007	98	0.0395	ortholog_one2one	*SMC4*
ENSMMUG00000017306	ENSG00000166851	100	0.0007	100	0.0651	ortholog_one2one	*PLK1*
ENSMMUG00000017345	ENSG00000135925	100	0	100	0.1127	ortholog_one2one	*WNT10A*
ENSMMUG00000017492	ENSG00000165512	99	0.0037	99	0.0336	ortholog_one2one	*ZNF22*
ENSMMUG00000017519	ENSG00000167721	97	0.0143	97	0.0716	ortholog_one2one	*TSR1*
ENSMMUG00000017570	ENSG00000091073	96	0.0162	96	0.1465	ortholog_one2one	*DTX2*
ENSMMUG00000017810	ENSG00000149136	100	0.0012	100	0.092	ortholog_one2one	*SSRP1*
ENSMMUG00000017843	ENSG00000111752	99	0.0051	99	0.0545	ortholog_one2one	*PHC1*
ENSMMUG00000017970	ENSG00000160633	99	0.004	99	0.055	ortholog_one2one	*SAFB*
ENSMMUG00000018001	ENSG00000124479	100	0	100	0.0755	ortholog_one2one	*NDP*
ENSMMUG00000018051	ENSG00000083857	96	0.0189	96	0.1354	ortholog_one2one	*FAT1*
ENSMMUG00000018187	ENSG00000213988	59	0.1938	53	0.3025	ortholog_many2many	
ENSMMUG00000018256	ENSG00000182481	99	0.0028	99	0.0733	ortholog_one2many	*KPNA2*
ENSMMUG00000018372	ENSG00000082556	93	0.0464	93	0.1764	ortholog_one2one	*OPRK1*
ENSMMUG00000018405	ENSG00000066923	95	0.0259	95	0.0827	ortholog_one2one	*STAG3*
ENSMMUG00000018549	ENSG00000197362	91	0.0418	91	0.1148	ortholog_one2one	*ZNF786*
ENSMMUG00000018725	ENSG00000111554	95	0.0213	96	0.047	ortholog_one2one	*MDM1*
ENSMMUG00000018737	ENSG00000051596	100	0.0013	100	0.0627	ortholog_one2one	*THOC3*
ENSMMUG00000018790	ENSG00000114739	100	0	97	0.0406	ortholog_one2one	*ACVR2B*
ENSMMUG00000018805	ENSG00000134323	98	0.0075	98	0.0882	ortholog_one2one	*MYCN*
ENSMMUG00000018806	ENSG00000099783	93	0.0385	93	0.1122	ortholog_one2one	*HNRNPM*
ENSMMUG00000018813	ENSG00000179172	89	0.0515	89	0.0925	ortholog_one2one	*HNRNPCL1*
ENSMMUG00000018816	ENSG00000185664	92	0.0248	94	0.0514	ortholog_one2one	*PMEL*
ENSMMUG00000018871	ENSG00000165119	100	0	100	0.0232	ortholog_one2one	*HNRNPK*
ENSMMUG00000018888	ENSG00000173876	96	0.0201	92	0.2471	ortholog_many2many	
ENSMMUG00000018929	ENSG00000088448	99	0.0046	99	0.0539	ortholog_one2one	*ANKRD10*
ENSMMUG00000019154	ENSG00000116560	91	0.0636	81	0.1444	ortholog_one2one	*SFPQ*
ENSMMUG00000019224	ENSG00000141562	87	0.0201	96	0.1035	ortholog_one2one	*NARF*
ENSMMUG00000019339	ENSG00000034053	98	0.0095	98	0.187	ortholog_one2one	*APBA2*
ENSMMUG00000019421	ENSG00000150776	97	0.0133	87	0.0575	ortholog_one2one	*C11ORF57*
ENSMMUG00000019474	ENSG00000088305	94	0.0414	93	0.1204	ortholog_one2one	*DNMT3B*

ENSMMUG00000019485	ENSG00000138587	98	0.0109	98	0.0574	ortholog_one2one	*MNS1*
ENSMMUG00000019529	ENSG00000137309	38	0.2877	64	0.504	ortholog_one2many	*HMGA1*
ENSMMUG00000019542	ENSG00000120438	92	0.0387	92	0.0763	ortholog_one2many	
ENSMMUG00000019576	ENSG00000257529	95	0.0407	78	0.0918	ortholog_many2many	
ENSMMUG00000019610	ENSG00000165724	100	0	100	0.0777	ortholog_one2one	*ZMYND19*
ENSMMUG00000019636	ENSG00000124207	100	0	100	0.0345	ortholog_one2one	*CSE1L*
ENSMMUG00000019637	ENSG00000089280	95	0.0128	96	0.0543	ortholog_one2one	*FUS*
ENSMMUG00000019674	ENSG00000180210	95	0.025	84	0.1102	ortholog_one2one	*F2*
ENSMMUG00000019785	ENSG00000100129	90	0.0253	96	0.1446	ortholog_one2one	*EIF3L*
ENSMMUG00000019854	ENSG00000144868	95	0.0279	95	0.0582	ortholog_one2one	*TMEM108*
ENSMMUG00000019876	ENSG00000170312	98	0.0015	100	0.0332	ortholog_one2one	*CDK1*
ENSMMUG00000020088	ENSG00000127952	63	0.2985	64	0.5758	ortholog_one2one	*STYXL1*
ENSMMUG00000020202	ENSG00000154545	98	0.0087	98	0.0631	ortholog_one2many	*MAGED4B*
ENSMMUG00000020218	ENSG00000154027	98	0.0062	99	0.0487	ortholog_one2one	*AK5*
ENSMMUG00000020349	ENSG00000091490	98	0.0096	86	0.0405	ortholog_one2one	*SEL1L3*
ENSMMUG00000020363	ENSG00000173933	66	0.135	66	0.2742	ortholog_many2man	*RBM4*
ENSMMUG00000020467	ENSG00000156802	96	0.016	96	0.0562	ortholog_one2one	*ATAD2*
ENSMMUG00000020508	ENSG00000273439	95	0.022	79	0.0905	ortholog_one2many	*ZNF8*
ENSMMUG00000020513	ENSG00000044446	99	0.0031	99	0.0639	ortholog_one2one	*PHKA2*
ENSMMUG00000020580	ENSG00000103494	97	0.0167	91	0.0419	ortholog_one2one	*RPGRIP1L*
ENSMMUG00000020643	ENSG00000077514	97	0.012	97	0.0353	ortholog_one2one	*POLD3*
ENSMMUG00000020668	ENSG00000105173	98	0.0085	98	0.0337	ortholog_one2one	*CCNE1*
ENSMMUG00000020799	ENSG00000015475	78	0.1322	68	0.3948	ortholog_one2one	*BID*
ENSMMUG00000020928	ENSG00000155438	94	0.0324	94	0.0662	ortholog_one2one	*MKI67IP*
ENSMMUG00000020982	ENSG00000169813	100	0	100	0.0445	ortholog_one2one	*HNRNPF*
ENSMMUG00000021023	ENSG00000175063	98	0.0051	99	0.1123	ortholog_one2one	*UBE2C*
ENSMMUG00000021037	ENSG00000143797	99	0.0064	99	0.0303	ortholog_one2one	*MBOAT2*
ENSMMUG00000021054	ENSG00000100297	93	0.0319	93	0.1865	ortholog_one2one	*MCM5*
ENSMMUG00000021070	ENSG00000100592	99	0.0037	99	0.0375	ortholog_one2one	*DAAM1*
ENSMMUG00000021081	ENSG00000123416	100	0	100	0.0445	ortholog_one2one	*TUBA1B*
ENSMMUG00000021173	ENSG00000001167	100	0	100	0.0293	ortholog_one2one	*NFYA*
ENSMMUG00000021221	ENSG00000123485	88	0.0644	87	0.0817	ortholog_one2one	*HJURP*

ENSMMUG00000021354	ENSG00000145708	98	0.0107	98	0.0588	ortholog_one2one	*CRHBP*
ENSMMUG00000021378	ENSG00000100276	41	0.2608	70	1.0423	ortholog_one2one	*RASL10A*
ENSMMUG00000021417	ENSG00000072415	97	0.018	97	0.0948	ortholog_one2one	*MPP5*
ENSMMUG00000021431	ENSG00000169583	98	0.0118	97	0.2377	ortholog_one2one	*CLIC3*
ENSMMUG00000021438	ENSG00000137707	97	0.0116	97	0.0579	ortholog_one2one	*BTG4*
ENSMMUG00000021497	ENSG00000130559	97	0.0167	80	0.1216	ortholog_one2one	*CAMSAP1*
ENSMMUG00000021525	ENSG00000062822	96	0.009	95	0.1697	ortholog_one2one	*POLD1*
ENSMMUG00000021820	ENSG00000143799	98	0.0086	98	0.0709	ortholog_one2one	*PARP1*
ENSMMUG00000021929	ENSG00000142945	98	0.0088	95	0.0558	ortholog_one2one	*KIF2C*
ENSMMUG00000021996	ENSG00000149256	99	0.0036	49	0.1086	ortholog_one2many	*ODZ4*
ENSMMUG00000022221	ENSG00000170004	98	0.0091	94	0.0867	ortholog_one2one	*CHD3*
ENSMMUG00000022356	ENSG00000006042	100	0.0001	90	0.0572	ortholog_one2one	*TMEM98*
ENSMMUG00000022364	ENSG00000165752	87	0.0672	59	0.6248	ortholog_one2one	*STK32C*
ENSMMUG00000022376	ENSG00000122952	96	0.0237	96	0.0774	ortholog_one2one	*ZWINT*
ENSMMUG00000022458	ENSG00000188994	98	0.0094	93	0.0481	ortholog_one2one	*ZNF292*
ENSMMUG00000022517	ENSG00000095627	96	0.0176	96	0.0606	ortholog_one2one	*TDRD1*
ENSMMUG00000022725	ENSG00000105202	96	0.0234	95	0.1055	ortholog_one2one	*FBL*
ENSMMUG00000022767	ENSG00000167658	100	0.0005	100	0.2929	ortholog_one2one	*EEF2*
ENSMMUG00000022791	ENSG00000160207	97	0.0162	97	0.1008	ortholog_one2one	*HSF2BP*
ENSMMUG00000022831	ENSG00000144524	80	0.1444	83	0.2374	ortholog_one2one	*COPS7B*
ENSMMUG00000022919	ENSG00000165209	100	0.0007	100	0.0293	ortholog_one2one	*STRBP*
ENSMMUG00000023126	ENSG00000170860	100	0	100	0.0608	ortholog_one2one	*LSM3*
ENSMMUG00000023199	ENSG00000183431	87	0.0996	88	0.1908	ortholog_one2one	*SF3A3*
ENSMMUG00000023352	ENSG00000181163	96	0.0119	96	0.0483	ortholog_one2one	*NPM1*
ENSMMUG00000023431	ENSG00000132780	92	0.0537	92	0.0785	ortholog_one2one	*NASP*
ENSMMUG00000023442	ENSG00000100099	89	0.0465	91	0.0952	ortholog_one2one	*HPS4*
ENSMMUG00000023463	ENSG00000143194	98	0.0076	98	0.043	ortholog_one2one	*MAEL*
ENSMMUG00000023490	ENSG00000109881	95	0.0205	94	0.0824	ortholog_one2one	*CCDC34*
ENSMMUG00000023496	ENSG00000069482	92	0.037	72	0.0834	ortholog_one2one	*GAL*
ENSMMUG00000023575	ENSG00000174718	93	0.0335	93	0.0799	ortholog_one2one	*KIAA1551*
ENSMMUG00000023684	ENSG00000196230	100	0	100	0.086	ortholog_one2one	*TUBB*
ENSMMUG00000023721	ENSG00000124610	91	0.0524	91	0.1234	ortholog_one2one	*HIST1H1A*

ENSMMUG00000024365	ENSG00000229686	99		99		ortholog_one2one	*SNORD56*
ENSMMUG00000025943	ENSG00000207392	95		95		ortholog_one2one	*SNORA20*
ENSMMUG00000026454	ENSG00000271798	99		99		ortholog_one2one	*SNORA51*
ENSMMUG00000026455	ENSG00000206630	98		98		ortholog_one2one	*SNORD60*
ENSMMUG00000027100	ENSG00000207547	100		100		ortholog_one2one	*MIR25*
ENSMMUG00000027425	ENSG00000199551	80		83		ortholog_many2man	*U6*
ENSMMUG00000028785	ENSG00000167635	100	0.0014	100	0.1057	ortholog_one2one	*ZNF146*
ENSMMUG00000028923	ENSG00000156795	97	0.014	97	0.0551	ortholog_one2one	*WDYHV1*
ENSMMUG00000029950	ENSG00000165502	97	0.0133	97	0.6418	ortholog_one2many	
ENSMMUG00000030131	ENSG00000106355	96	0.0206	96	0.0751	ortholog_one2one	*LSM5*
ENSMMUG00000031431	ENSG00000163923	98	0.0122	98	0.0323	ortholog_one2one	*RPL39L*
ENSMMUG00000031442	ENSG00000143621	96	0.0173	89	0.0375	ortholog_one2many	
ENSMMUG00000031717	ENSG00000165502	99	0.0042	99	0.6064	ortholog_one2many	
ENSMMUG00000032760	ENSG00000212607	98		98		ortholog_one2one	*SNORA3*
ENSMMUG00000032819	ENSG00000212498	100		100		ortholog_one2one	*SNORD86*
ENSMMUG00000033381	ENSG00000216133	98		99		ortholog_one2one	*MIR939*
ENSMMUG00000021098	ENSG00000153187	99	0.0079	91	0.0474	ortholog_one2one	*HNRNPU*
ENSMMUG00000009055	ENSG00000165973	95	0.0064	99	0.0414	ortholog_one2one	*NELL1*
ENSMMUG00000022862	ENSG00000050165	97	0.0131	97	0.1343	ortholog_one2one	*DKK3*
ENSMMUG00000015777	ENSG00000152795	99	0.0045	99	0.0369	ortholog_one2one	*HNRPDL*
ENSMMUG00000004005	ENSG00000073584	100	0	100	0.0271	ortholog_one2one	*SMARCE1*
ENSMMUG00000005575	ENSG00000124496	94	0.0449	94	0.1038	ortholog_one2one	*TRERF1*
ENSMMUG00000022926	ENSG00000185787	99	0.0026	99	0.0452	ortholog_one2one	*MORF4L1*

*dN, the ratio of Nonsynonymous substitutions per non-synonymous site; #dS, the ratio of synonymous substitutions per synonymous site; Red, upregulated in old ovaries; Green, downregulated in old ovaries. Co-down indicates the genes downregulated in old monkey ovaries compare to young and middle-aged monkey ovaries.
